# Targeting patient recovery priorities in degenerative cervical myelopathy: design and rationale for the RECEDE-Myelopathy trial—study protocol

**DOI:** 10.1136/bmjopen-2022-061294

**Published:** 2023-03-07

**Authors:** Benjamin Davies, Oliver D Mowforth, Stefan Yordanov, Daniel Alvarez-Berdugo, Simon Bond, Marianna Nodale, Paula Kareclas, Lynne Whitehead, Jon Bishop, Siddharthan Chandran, Sarah Lamb, Mark Bacon, Marios C Papadopoulos, Michelle Starkey, Iwan Sadler, Lara Smith, Sukhvinder Kalsi-Ryan, Adrian Carpenter, Rikin A Trivedi, Martin Wilby, David Choi, Ian B Wilkinson, Michael G Fehlings, Peter John Hutchinson, Mark R N Kotter

**Affiliations:** 1 Department of Neurosurgery, Cambridge University, Cambridge, UK; 2 Cambridge Clinical Trials Unit, Cambridge University Hospital, Cambridge, UK; 3 Pharmacy Department, Cambridge University Hospitals NHS Foundation Trust, Cambridge, UK; 4 Medical Statistician, NIHR Surgical Reconstruction and Microbiology Research Centre, Birmingham, UK; 5 Edinburgh Medical School & Centre for Clinical Brain Sciences, University of Edinburgh, Edinburgh, UK; 6 Institute of Health Research, University of Exeter, Exeter, UK; 7 International Spinal Research Trust, London, UK; 8 Department of Neurosurgery, St George's Hospital, London, UK; 9 Myelopathy.org, Cambridge, UK; 10 Toronto Rehabilitation Institute, Toronto, Ontario, Canada; 11 Department of Clinical Neurosciences, University of Cambridge, Cambridge, UK; 12 Department of Neurosurgery, The Walton Centre NHS Foundation Trust, Liverpool, UK; 13 Department of Neurosurgery, National Hospital for Neurology and Neurosurgery, London, UK; 14 Department of Surgery, Toronto Western Hospital and University of Toronto, Toronto, Ontario, Canada

**Keywords:** neurosurgery, spine

## Abstract

**Introduction:**

Degenerative cervical myelopathy (DCM) is a common and disabling condition of symptomatic cervical spinal cord compression secondary to degenerative changes in spinal structures leading to a mechanical stress injury of the spinal cord. RECEDE-Myelopathy aims to test the disease-modulating activity of the phosphodiesterase 3/phosphodiesterase 4 inhibitor Ibudilast as an adjuvant to surgical decompression in DCM.

**Methods and analysis:**

RECEDE-Myelopathy is a multicentre, double-blind, randomised, placebo-controlled trial. Participants will be randomised to receive either 60–100 mg Ibudilast or placebo starting within 10 weeks prior to surgery and continuing for 24 weeks after surgery for a maximum of 34 weeks. Adults with DCM, who have a modified Japanese Orthopaedic Association (mJOA) score 8–14 inclusive and are scheduled for their first decompressive surgery are eligible for inclusion. The coprimary endpoints are pain measured on a visual analogue scale and physical function measured by the mJOA score at 6 months after surgery. Clinical assessments will be undertaken preoperatively, postoperatively and 3, 6 and 12 months after surgery. We hypothesise that adjuvant therapy with Ibudilast leads to a meaningful and additional improvement in either pain or function, as compared with standard routine care.

**Study design:**

Clinical trial protocol V.2.2 October 2020.

**Ethics and dissemination:**

Ethical approval has been obtained from HRA—Wales. The results will be presented at an international and national scientific conferences and in a peer-reviewed journals.

**Trial registration number:**

ISRCTN Number: ISRCTN16682024.

**Strengths and limitations of this study**

- Significant patient and public involvement in trial design and outcomes planning.
- A pragmatic approach to patient inclusion criteria was utilised—all patients with modified Japanese Orthopaedic Association score between 8 and 14 and MRI findings of degenerative cervical myelopathy (DCM) who are scheduled for their first surgery for DCM regardless of approach are able to be included.
- We will explore and compare both clinical and objective findings and validated questionnaire and multiple patient-reported outcomes.
- A limitation is the need of close patient follow-up and rigorous screening with additional blood tests to comply with drug monitoring and assessments needed.

## Introduction

Here we present the study rationale and design of **Re**generation in **Ce**rvical **De**generative Myelopathy (RECEDE-Myelopathy), the first regenerative medicine trial for degenerative cervical myelopathy (DCM), which aims to test disease-modulating activity of the phosphodiesterase (PDE)3/PDE4 inhibitor Ibudilast as an adjuvant to surgical decompression.

### DCM is a common and progressive condition with devastating impact on quality of life

DCM is the most common cause of spinal cord impairment worldwide,^1 2^ with some estimates of the prevalence as high as 2% of adults.^2–4^ It arises when arthritic or developmental changes in the cervical spine compress the spinal cord, causing a progressive slow-motion spinal cord injury.^5^ As a degenerative pathology the incidence is expected to rise in an ageing population.^6 7^

The consequences of DCM are numerous, varied and often progressive. Symptoms include pain, loss of dexterity, imbalance and frequent falls, incontinence and in extreme circumstances paralysis.^1 8–11^ A recent comparative study found sufferers have among the worst quality of life scores of all chronic disease,^12 13^ and this is likely to also negatively impact on their supporters.^14^ The cost of DCM to society has not been measured yet, but it is likely to be significant. Consequently, improving recovery after surgery is a significant unmet need and there is strong evidence that surgical treatment for DCM is cost-effective.^15^

### Surgery is the only evidence-based treatment for DCM

At present, the only effective treatment for DCM is surgery. While surgery can stop disease progression, the existing damage does not fully recover^16^ and people with DCM retain life-long disabilities with severe impact on quality of life.^12 13^ Many remain unable to return to full time work and reliant on others for day-to-day activities.^17^ Given the severe long-term consequences of DCM, treatment alternatives that promote recovery are desperately needed.

### PDE3 inhibition promotes functional recovery in preclinical DCM

The mitogen-activated protein kinases (MAPKs) play a vital role in intracellular signalling.^18^ In response to extracellular stimuli, such as neurotransmitters, inflammatory factors or stress conditions, this family of interconnected serine/threonine kinases coordinates a diverse range of intracellular processes, including cell differentiation, proliferation and apoptosis, inflammation and stress responses.^19^ This signalling pathway and its modulation have therefore been linked to many diseases including cancer, asthma, stroke, multiple sclerosis (MS) and Alzheimer’s dementia. More recently, preclinical studies, including our own, have demonstrated that its modulation via inhibition of a class of enzymes called PDEs can improve functional recovery and reduce the perception of pain following damage to the central nervous system (CNS).^20–22^

PDEs hydrolyse the intracellular messenger cyclic AMP (cAMP).^20 23^ This results in modulation of MAPK signalling.^24 25^ Inhibition of PDE3 is particularly attractive in DCM as treatment with the selective PDE3 inhibitor cilostozol resulted in improved functional recovery in a rat model of DCM,^26^ likely by improving latent ischaemia.

### Improvements following surgery are associated with axon sprouting, remyelination and immunomodulation

In DCM, tethering and compression of the spinal cord initiates a cascade of secondary injury events, including ischaemia, inflammation and apoptosis that ultimately cause increased neurological deficits.^5 27 28^ The partial reversal of symptoms after surgery highlights an inherent, although attenuated, regenerative capacity of the spinal cord.^16 29^ This is echoed by postmortem studies and our preclinical data, which indicate that neurological recovery following decompression is associated with axonal plasticity, remyelination and modulation of the immune response.^29–31^ Enhancing axonal plasticity and remyelination is therefore key to improving outcomes after DCM.^32^

### PDE4 inhibition can promote functional recovery and modulate pain in preclinical models

PDE4 is another isoform of PDE inhibitors, which has demonstrated preclinical benefits on axon outgrowth^20^ and remyelination.^22^ The best characterised application of PDE4 inhibitors involves preclinical models of traumatic spinal cord injury using a drug called rolipram.^20 21^ Unanimously, these have demonstrated that modulation of the PDE4 cascade is able to benefit recovery. In addition, our own work demonstrated that inhibition of PDE4 is able to stimulate the regenerative response of a CNS stem cell population termed oligodendrocyte progenitor cells and engage in remyelination,^22^ a process that has been observed in postmortem spinal cords affected by DCM.^30^

PDE4 inhibition also has a role in modulating the perception of pain. Central to the development and maintenance of chronic pain syndromes is glial activation within the CNS, which enhances pain sensitivity via neuronal–glial interactions.^33^ Modulation of MAPK via PDE4 inhibition has demonstrated a reduction in pain in several preclinical models.^34–37^ Bao *et al* (2011) found that PDE4 inhibition improved not just motor recovery but also resulted in a reduction in neuropathic pain in a rat model of spinal cord injury.^38^ PDE4 inhibition also has an anti-inflammatory effect, increasing cAMP production in leucocytes and therefore reducing the release of tumour necrosis factor-alpha, a potent inflammatory mediator and peripheral pain stimulus.^39^

### Ibudilast is a potent PDE4 inhibitor with an excellent human safety profile

The majority of preclinical studies described have used rolipram for PDE4 inhibition. While rolipram is a potent and selective PDE4 inhibitor, experience from translational trials, most recently in MS,^40^ has demonstrated poor tolerability in humans due to significant nausea and vomiting. The MS trial had to be terminated due to a lack of efficacy and poor tolerability. Additionally, preclinical evidence has demonstrated a narrow therapeutic window, with potentially adverse neurological sequalae if missed.

An alternative is Ibudilast (MN-166).^23^ Ibudilast is a potent PDE4 inhibitor, with additional PDE3 and PDE5 receptor activity. Modulation of PDE3 is also attractive in DCM as it led to improved function in a preclinical model of DCM.^26^ Another attractive feature of Ibudilast is that it has been in clinical use for over 20 years for the treatment of asthma and poststroke dizziness, without tolerability issues.^41^

Ibudilast is currently under investigation for a number of other neurological conditions, including alcohol (NCT03489850) and methamphetamine (NCT01860807) addiction, glioblastoma (03782415), amyotrophic lateral sclerosis (ALS)^42^ and MS^43^ in a series of double-blind, placebo randomised controlled trials.

For ALS, a single Phase I/II trial has been completed. Two ALS cohorts of early stage disease and advanced stage disease requiring ventilation were randomised 1:1 to receive Ibudilast or placebo. Overall, the primary endpoint of safety and tolerability was met. In the early stage disease takers, Ibudilast was associated with a significant increase in survival, and delayed requirement for ventilation.^44^ Treatment effects were linked to per-protocol adherence to therapy.^45^ A Phase III trial is now planned.

For MS, two phase II trials have been completed. The first one evaluated relapsing remitting MS; while it did not prevent the development of new brain lesions, it slowed the progression of brain atrophy in a dose-dependent fashion. The second one, a follow-up study in progressive MS, found that Ibudilast significantly slowed the progression of brain atrophy.^46^

Of note, typical daily dosing in these trials ranged from 60 to 100 mg, which is greater than the currently licensed dosing of 10–20 mg per day for routine clinical practice. While trials confirmed overall tolerability and safety for use of Ibudilast in these doses in humans, findings do indicate a dose-dependent relationship for gastrointestinal side effects, such as nausea, and headaches and, in a minority of cases, this led to discontinuation of therapy by participants.

### RECEDE-Myelopathy

RECEDE-Myelopathy is a multicentre, double-blind, randomised, placebo-controlled trial assessing the efficacy of Ibudilast as an adjuvant treatment to decompressive surgery for DCM. The specific mechanism of action of Ibudilast is highly suited to address both functional outcome and neuropathic pain in DCM. Therefore, prompted by the direct involvement of people with DCM in designing the study, RECEDE-Myelopathy has an infrequently used study design of two coprimary endpoints. It is designed and powered to detect response of patients to Ibudilast with regards to function or pain, independently, as well as a response to both endpoints. We hypothesise that Ibudilast promotes functional outcome and reduces pain in surgically treated DCM.

## Methods

### Study design and objectives

RECEDE-Myelopathy is a multicentre, double-blind, randomised, placebo-controlled trial assessing the efficacy of Ibudilast as an adjuvant treatment to decompressive surgery for DCM. Participants will be randomised to receive either 60–100 mg Ibudilast (interventional arm) or placebo (control arm) starting within 10 weeks prior to surgery and continuing for 24 weeks after surgery for a maximum of 34 weeks of treatment. Preoperative treatment may leverage the effects of inhibition of PDE3, while postoperative treatment aims at regeneration-inducing effects outlined above. The primary objective will be to compare improvement in pain or physical function at 6 months after surgery between the two arms of the trial. We hypothesise that adjuvant therapy with Ibudilast leads to a meaningful and additional improvement in either pain or function, as compared with standard routine care (decompressive surgery). Planned start date for study recruitment is September 2021, with planned end being September 2025.

### Patient and public involvement (PPI)—aligning research with patient priorities

The involvement of public and patients representatives in research is recognised to be of key importance to ensure it delivers meaningful, practice-changing information.^47–50^ As with many fields, this has been a problem for DCM.^51–53^ To address this issue, we founded Myelopathy.org, the first and so far only charity for people with DCM. While in its infancy, the platform has become an international focus for people with DCM, hosting a peer-to-peer support community (Myelopathy Support) of over 2000 users.^54^ This has enabled larger-scale insights into the perspective of individuals with DCM,^17 55 56^ and ultimately led to RECODE-DCM, a James Lind Alliance-led initiative to identify and define the research priorities for DCM^51^ (https://aospine.aofoundation.org/research/recode-dcm)

### Definition of recovery priorities for people with DCM

AO Spine Research Objectives and Common Data Elements for Degenerative Cervical Myelopathy (AO Spine RECODE-DCM) is an international initiative to create a 'Research Toolkit' to help improve and accelerate knowledge gained in DCM and help to improve outcomes. As part of RECODE-DCM, a focus group of people with DCM was created with the objective to develop recovery domains. These were subsequently prioritised via an international, online survey (n=485).^10^ In contrast to the research focus to date,^52 57^

pain emerged as the number one recovery priority, closely followed by hand, and walking function. Consequently, the development of adjuvant treatments for DCM should be most usefully focused on reducing pain and improving limb function.

### Patient screening and eligibility

**Figure 1:**
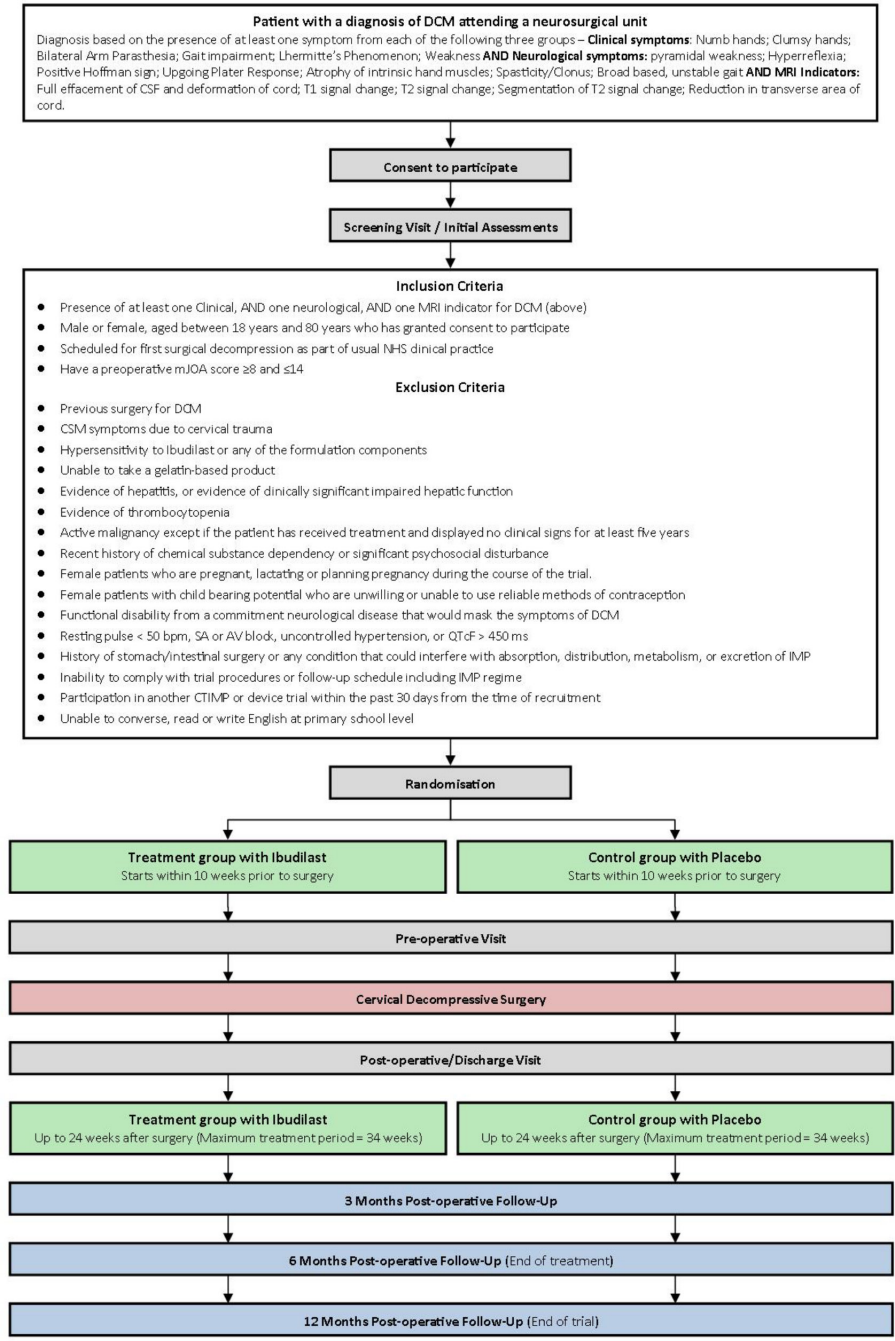
Trial flow chart. Eligible and consenting participants will be randomised to an intervention or control arm and followed up for 12 months after surgery. DCM, degenerative cervical myelopathy; IMP, investigational medicinal product.

A summary of the study flow diagram, including full inclusion and exclusion criteria, is presented in figure 1. In summary, adults (aged 18–80) with a diagnosis of DCM (participants must have at least one MRI indicator, clinical symptom and neurological sign from table 1 to be eligible for inclusion) and a disease severity of modified Japanese Orthopaedic Association (mJOA) score 8–14 inclusive, scheduled for their first decompressive surgery, will be approached to consider participation in RECEDE-Myelopathy.

**Table 1:** Trial criteria for diagnosis of DCM

MRI indicators	Clinical symptoms	Neurological signs
Effacement of cerebrospinal fluid (CSF) and deformation of cord	Numb hands	Pyramidal weakness
T1 signal change	Clumsy hands	Hyper-reflexia
T2 signal change	Bilateral arm paraesthesia	Positive Hoffman sign
Segmentation of T2 signal change	Gait impairment	Upgoing plantar response
Reduction in transverse area of cord	Lhermitte’s phenomenon	Atrophy of intrinsic hand muscles
	Weakness	Spasticity/clonus
		Broad based, unstable gait

Participants must have at least one MRI indicator, clinical symptom and neurological sign to be eligible for inclusion.

DCM, degenerative cervical myelopathy.

Eligibility will be further assessed against exclusion criteria, largely dictated by safety requirements for use of Ibudilast, and in precluding masking of treatment effects. This includes, concomitant lumbar canal stenosis or other neurological condition, presentation with symptoms due to trauma (eg, central cord syndrome), a history of allergy to Ibudilast, any of its formulation or that of the placebo, pregnancy, unwillingness to use reliable contraception, active malignancy, liver impairment or thrombocytopaenia. The latter will be assessed via serum biochemistry and haematological assessment. A full list of exclusion criteria can be found in table 2.

**Table 2:** Exclusion criteria

1	Previous surgery for degenerative cervical myelopathy
2	Degenerative cervical myelopathy symptoms due to cervical trauma, determined at the discretion of the investigator
3	Hypersensitivity to Ibudilast or any of the formulation components
4	Evidence of acute hepatitis, clinically significant chronic hepatitis or evidence of clinically significant impaired hepatic function through clinical and laboratory evaluation (including alkaline phosphatase (ALP) > 1.5 × upper limit of normal (ULN); alanine transaminas (ALT) or aspartate transaminase (AST) > 2 × ULN; gamma-glutamyl transferase (GGT) > 3 × ULN)
5	Evidence of thrombocytopaenia at screening through laboratory evaluation including platelet count <5000
6	Active malignancy defined as a history of invasive malignancy, except if the patient has received treatment and displayed no clinical signs and symptoms for ≥5 years
7	Recent history (≤3 years) of chemical substance dependency or significant psychosocial disturbance that may impact the outcome or trial participation
8	Female patients with childbearing potential who are unwilling or unable to use reliable methods of contraception
9	Female patients who are pregnant, lactating or planning pregnancy during the course of the trial
10	Inability to comply with trial procedures or follow-up schedule including investigational medicinal product (IMP) regime
11	Unable to take gelatin-based product
12	Participation in another clinical trial of an investigational medicinal product (CTIMP) or device trial ≤30 days before the time of recruitment
13	Functional disability from a concomitant neurological disease that would mask the symptoms of degenerative cervical myelopathy, determined at the discretion of the investigator. Including but not limited to stroke with a residual disability, cerebellar ataxia, Parkinson’s disease, symptomatic lumbar stenosis and multiple sclerosis
14	Resting pulse <50 bpm, sinoatrial or atrioventricular block, uncontrolled hypertension or corrected QT interval (QTcF) >450 ms
15	History of stomach or intestinal surgery or any other condition that could interfere with, or is judged by the investigator to interfere, with absorption, distribution, metabolism or excretion of IMP
16	Unable to converse, read or write English

IMP, investigational medicinal product.

### Enrolment and randomisation

Those patients who satisfy the screening criteria and agree to study participation are enrolled and randomised at 1:1 to one of the two treatment arms. A web-based randomisation system (sealed envelope) performing stratified blocked randomisation will be used stratifying by baseline mJOA (<12 vs ≥12), age (<60 years vs ≥60 years) and time to onset of the disease (>6 months vs ≤6 months); random block size will be used. Throughout randomisation and follow-up, the subjects, physicians and data collectors remain blinded to group allocation.

### Treatment description and dosage modification

The investigational medicinal product (IMP) is a 24–34-week course of Ibudilast or matched placebo in an escalating dosage regimen up to a maximum of 100 mg daily if tolerated. The escalating dosage regimen is to minimise gastrointestinal side effects. Ibudilast is available in 10 mg capsules, and therefore the IMP will be provided as such. The placebo is identical in shape, size and colour to the Ibudilast capsule, and participants will be provided with the same instructions.

Participants will start treatment within 10 weeks prior to surgical decompression and will continue taking drug for up to 24 weeks postsurgery. The excretion half-life of Ibudilast is approximately 20 hours. The IMP will be taken in divided doses, twice daily, morning and evening, for a maximum of 34 weeks. Because this is the first surgical trial with Ibudilast, and to mitigate any potential interference on the coagulation system, treatment will be halted 5 days prior to surgery and resumed at the previous maximum dose right after operation.

Ibudilast is associated with gastrointestinal side effects, such as nausea and dyspepsia. Alongside dose escalation, participants will be instructed to take trial medication with food or within an hour of eating to improve gastrointestinal tolerability. In the event of minor gastrointestinal complaints, participants will be offered symptomatic treatment in the first instance, in conjugation with ongoing IMP therapy. If this is unsuccessful, or not agreeable to participants, the trial therapy will be decreased in decrements of 20 mg every 5 days, until a tolerable dosing level is achieved, or the drug is stopped. If a participant cannot tolerate a minimum daily dosage of 60 mg despite additional supportive measures, treatment within the trial will be stopped.

### Surgery

There are a number of different approaches used to decompress the spinal cord in DCM. No surgical approach has been shown to be superior, and the consensus is that the approach needs to be tailored to the specific anatomy. The surgical care of participants will therefore be at the discretion of the treating clinician and not protocolised.

### Outcome measures and follow-up

#### Two patient-informed coprimary endpoints: pain and function

Inhibiting PDE3 and PDE4 with Ibudilast has the potential to benefit both pain and functional recovery by promoting repair mechanisms in the spinal cord as well as exerting neuroprotective effects. This provides a unique opportunity to address the most important recovery priorities identified by individuals with myelopathy. Therefore, RECEDE-Myelopathy has two outcome targets: pain and physical function.^10^ These coprimary endpoints will be assessed at 6 months after surgery, a time point when the majority of recovery will have been achieved.^57^

The study is thus powered to detect meaningful changes with regards to the coprimary endpoints independently from each other, that is, it is designed to establish whether Ibudilast has beneficial effects on function or pain alone or whether it beneficially modulates both end points.

##### Coprimary endpoint 1

The international standard, and most validated measure for assessment of function in DCM, is the mJOA score.^16 58 59^ The mJOA is a composite score of upper and lower limb muscular function, upper limb sensory function and bladder function.

##### Coprimary endpoint 2

Pain has been identified as the recovery priority of patients with DCM. The most common form is neck pain,^9^ with a neuropathic component that is responsive to neuroprotective treatments.^60 61^

While numerous tools have been developed for the measurement of pain,^62^ the Initiative on Methods, Measurements and Pain Assessment in Clinical Trials agree that pain intensity scales provide the most relevant outcome measure for demonstrating efficacy. In DCM the visual analogue scale (VAS) is the most popular example of this.^63^ Although not exclusively validated for DCM, the psychometric properties of VAS neck and VAS arm pain have been evaluated in degenerative disease of the cervical spine,^64 65^ with VAS neck pain having better repeatability.

This design will address the most important priorities of people with DCM.^10^ It leverages the mechanism of action of Ibudilast to maximise the chances of demonstrating the benefit of the studied intervention. It will increase the knowledge that can be gained through the study and demonstrate whether the proposed mechanisms of neuroprotection and regeneration can be applied to promote function and/or reduce pain. Finally, the dual end-point design will make the study more efficient than conducting two independent trials. The chosen two endpoint design will hence increase the value of the study.

### Secondary and exploratory endpoints

Clinical assessments will additionally be undertaken preoperatively, postoperatively and 3, 6 and 12 months after surgery. The disability reported in the context of DCM is wide ranging. In the absence of a consensus dataset,^51^ an issue that we are currently attending to as part of RECODE-DCM, a variety of clinician administered and patient-reported outcome measures will be used to provide a comprehensive assessment. A full list of assessments and their time points is presented in table 3.

**Table 3:** Schedule of assessments

Assessments	Screening visit and initial assessments	Randomisation	Start of IMP (within 2–3 months prior to surgery)	Preoperative assessments (within 21 days prior to surgery)	Surgery	Postoperatively/ discharge (within 14 days postsurgery)	3 Months postoperatively (±21 days)	6 Months post operatively (±21 days)	12 Months post operatively (±21 days)
Informed consent	**X**								
Eligibility assessment	**X**								
Demographics	**X**								
Medical history and DCM characteristics	**X**								
Concomitant medication	**X**			**X**			**X**	**X**	**X**
Blood tests (FBC, LFT, E/U/C, TFTs)	**X**			**X**			**X**	**X**	**X**
ECG	**X**								
Urine analysis	**X**								
Pregnancy test	**X**								
Randomisation		**X**							
Neurological examination	**X**			**X**		**X**	**X**	**X**	**X**
mJOA	**X**			**X**			**X**	**X**	**X**
30 m Walk test	**X**			**X**			**X**	**X**	**X**
GRASSP-cervical myelopathy	**O**			**O**			**O**	**O**	**O**
SCIMv3	**O**			**O**				**O**	
WHO performance status				**X**					
Neck disability index	**O**			**O**		**O**	**O**	**O**	**O**
VAS pain	**X**			**X**		**X**	**X**	**X**	**X**
SF-36	**X**			**X**			**X**	**X**	**X**
EQ5D/health resource usage	**X**			**X**			**X**	**X**	**X**
Quick-DASH	**O**			**O**			**O**	**O**	**O**
Carer QoL (substudy)	**X**			**X**			**X**	**X**	**X**
Review of AEs			**X**	**X**		**X**	**X**	**X**	**X**
Dosing diary	**X**								
Dispensing of IMP			**X**			**X**	**X**		
Serum sample for PK studies	**X**			**X**	**X**		**X**	**X**	**X**
Compliance assessment				**X**		**X**	**X**	**X**	
IMP review				**X**		**X**	**X**	**X**	
Respiratory physiology and muscle function				**X**				**X**	
MRI				**X**				**X**	
Gait lab (substudy)				**X**			**O**	**X**	
Surgery details					**X**				
Surgery complications						**X**	**X**	**X**	**X**
Hospital discharge						**X**			
CSF sample					**O**				

**X: mandatory assessment; O: optional assessment**

DCM, degenerative cervical myelopathy; IMP, investigational medicinal product; mJOA, modified Japanese Orthopaedic Association; VAS, visual analogue scale.

Not all assessments will be conducted at every time point, or be mandated, to reduce participant and investigator burden. Assessment is also extended to carers of participants. Building on our preliminary finding of reduced quality of life among DCM carers,^14^ the Care Quality of Life instrument (CarerQol) will be used to evaluate this.^66^

### Adaptive sample size design

The minimum clinically important difference (MCID) for the mJOA is estimated to be between 1 and 2 points.^67^ Although not exclusively validated for DCM, the MCID for VAS neck and VAS arm pain has been calculated for degenerative disease of the cervical spine with values ranging from 8 to 26 mm.^64 65^ Both VAS pain and mJOA improve more than the MCID with surgery alone,^57^ and the amount of change is linked to the pre-operative baseline.^67^ Consequently, in consensus with patients we have determined the MCID of the VAS pain score as being 1 cm and for the mJOA 1 point. This has been modelled to ensure statistical power across all baseline scenarios.

On this basis, a total sample size of 362 participants under equal randomisation will provide 85% power to detect a difference of 1 between treatment arms on the mJOA scale (assuming a SD of 2.89), using a two-sided t-test at a 2.5% significance level to adjust for multiple comparisons.^68^ The trial is also powered to detect a similar difference on the VAS neck pain scale (assuming a difference of 1 and a SD of 2.88).

A blinded interim analysis will be conducted to refine the power calculation. The aim will be to reassess the sample size in time to allow any potential extension and increase in sample size to be put into effect. Reduction in sample size will not be permitted. Any sample size increase will be based on checking the assumption regarding the SD, and will not estimate any treatment effect, hence no subsequent adjustment to future analyses is needed.

Under such a framework, the theoretical optimal time to schedule such an interim analysis would be just as the last patient is recruited under the original sample size (n=362) following which a decision could be taken to either halt or extend recruitment. However, for reasons of practicality a window for the interim analysis will be up to a period of 4 months before reaching the total sample size.

The SD and correlation of both endpoints will be reassessed using data pooled across the arms. The three possible statistically significant conclusions of the formal hypothesis testing (VAS; mJOA; both) will be provided with revised target sample sizes needed to achieve 85% power under the same MCID values, but with revised estimates for the SD values and correlation. A recommended revised sample size will be the smallest of the three new target sample sizes or the original sample size if this is larger; hence the recommended sample size will never be a reduction from the original.

The next step of the interim analysis will be to calculate the conditional power of the three possible positive outcomes based on, the estimated unblinded treatment effects from the current data, plus, the distribution of future data from the revised sample size under the corresponding combinations of true treatment effects (MCID or zero), and SD and correlation estimates from the first step. If all three conditional power values are <30% then the recommendation would be to halt the study.

### Trial monitoring

All data collected during the trial will be recorded into a Case Report Form (CRF), which will be labelled using a participant’s unique trial ID and date of birth. CRFs will be completed by the local research team and copies will be sent to trial coordination centre, where it will be entered into a central digital database. Safety assessments will be conducted by local investigators and reported and handled according to a predefined trial protocol. This includes a mechanism to capture surgical complications.^69^ The Trial Steering Committee (TSC) will provide overall supervision with respect to the conduct of the trial. The TSC will consist of an independent Chairperson (Prof Michael Fehlings), a PPI representative (Mr Iwan Sadler), independent clinical and science experts (Prof Marios Papadopoulos and Dr Mark Bacon), clinical pharmacology and neurosurgery experts (Prof Ian Wilkinson and Prof Peter Hutchinson), the Chief Investigator and members of the Trial Management Group (eg, trial statistician, trial manager). The ethical and safety aspects of the trial will be overseen by an independent Data Monitoring Committee (DMC) who will meet once a year and their meetings will be timed so that reports can be fed into the TSC meetings. Safety assessment will be performed for every participant since consent and until end of their participation in the trial. To date, there are no known expected serious adverse reactions (SARs) for Ibudilast, and thus any reported SAR will be considered a suspected unexpected serious adverse reaction. Furthermore, surgical complications will be followed up as events of special interest to be reviewed by the DMC.

### Statistical methods

The primary endpoint and key secondary endpoints are all measured on a continuous scale. A comparison of mean values between treatment arms, adjusting for baseline covariates, will be provided using linear regression. Estimates, standard errors, 95% CIs and p values will be provided.

For formal hypothesis testing, a closed testing approach will be used to deal with multiple endpoints.^70^ Initially either of the coprimary endpoints (mJOA or VAS neck pain) may test a null hypothesis of 0 mean difference at a two-sided 2.5% significance level,^71^ with the remaining primary endpoint tested at 5% significance level. This will enable us to determine whether the study drug is effective on pain or function independently.

Subsequently a gate-keeping approach will be used where an endpoint below the primary endpoint in the prespecified ordering is only tested if all the preceding endpoints reject the null hypothesis, using the nominal p value. If an endpoint does not reject the null, then all endpoints below it have the same conclusion, not rejecting the null, regardless of their nominal p value. The ordering is, after primary endpoints, Short Form Survey (SF-36) Physical Component Summary (PCS) and then SF-36 Mental Component Summary (MCS).

Secondary endpoints will be compared between treatment arms using approach regression techniques: linear regression for continuous endpoints, logistic regression for binary endpoints, and Cox regression for time-to-event.

The following baseline covariates, in addition to the baseline value of the endpoint, will be used to adjust all comparisons

- Time to onset
- Smoking status (yes/no)
- Age
- Psychiatric comorbidities (yes/no)
- Impaired gait (yes/no)

A detailed statistical analysis plan will be produced before the final database lock.

## Discussion

This is the first regenerative medicine trial for DCM. It is also the first trial to target all the recovery priorities for people with DCM, namely pain and upper and lower limb function as primary endpoints.^10^ This is significant, as in the recent evaluation of Riluzole as a perioperative neuroprotective therapy in DCM, while the primary endpoint (1-point change in mJOA) was not met, VAS neck pain, a secondary endpoint, improved significantly.^58^ However, as a secondary endpoint the causal link can only be tentative.

### RECEDE-Myelopathy addresses 5 of the 10 top priorities identified by RECODE-DCM

Priority 1—raising awareness^1 72^:

1. RECEDE-Myelopathy is the first regenerative medicine trial for DCM. It is the second powered DCM CTIMP worldwide. We will seek to leverage this fact to attract attention to DCM by optimising communication before, during and after the trial, aiming at maximising our audience, to include patient organisations, a wide range of healthcare providers and the scientific community. We also aim to break into non-specialist mainstream media.

Priority 2—assessment and monitoring:

1. RECEDE-Myelopathy will help to standardise assessment and monitoring across study centres, and thus promote the implementation of the recent international guidelines.^16^ Additionally, a number of new secondary endpoints are included for the first time in a clinical trial of DCM, including gait^73^ and respiratory physiology.^74^

Priority 5—developing a better understanding of the pathophysiology of DCM^75^:

1. RECEDE-Myelopathy tests the hypothesis that MAPK signalling mediated by PDE3/4 can promote recovery after DCM. It will serve as a platform for substudies, including imaging studies, and molecular biology studies on blood draws and CSF.

Priority 6—rehabilitation:

1. There are no evidence-based measures to promote rehabilitation in DCM.^76^ RECEDE-Myelopathy will investigate a drug that has the possibility of improving functional outcomes in DCM.

Priority 7—novel therapies:

1. At present, surgery is the only possible treatment for DCM. If successful, RECEDE-Myelopathy will pave the way for the first evidence-based non-surgical adjuvant treatment.

### Neuropathic origins of neck pain in DCM

Pain has been identified as the recovery priority of people with DCM.^10^ Where assessed, previous trials have focused on neck (or axial) pain and arm pain.^58 61 77^ Our findings in a survey of 230 patients found that neck pain was the most commonly reported first symptom of DCM (13%), and with respect to pain, twice as common as limb pain (7%). Moreover, overall neck pain was experienced more often (80%) than arm pain (70%).^56^ In addition, individuals can be affected by atypical pain syndromes such as headache.^8 11 78^

Counter to the prevalent belief that neck pain is mainly caused by arthritic changes to the spine, an emerging literature points to a neuropathic origin. First, arthritic changes are omnipresent with progressive age, causing increasing levels of cord compression.^3 9^ In many instances this does not lead to neck pain, even in the context of DCM.

A neuropathic component of chronic neck pain has long been postulated. For example, a psychophysical study measuring responses to electrocutaneous stimulation in subjects with chronic neck pain found evidence of secondary hyperalgesia which, in turn, implies central sensitisation of nociceptive pathways.^79^ The results were compatible with studies which identify potential anatomical origins of chronic neck pain but provide evidence that central sensitisation may be the relevant mechanism of pain production.

A single-centre study investigated the relation between pain provoking cervical segments identified by diagnostic dorsal root blockades and elevation of quantitative sensory testing of the cervical dermatomes using Semmes-Weinstein monofilaments in patients suffering from neck pain but not radiculopathy. This revealed a systematic elevation of detection thresholds, an adaptation in contrast with, but not contradictory to, central sensitisation of high-threshold neurons in chronic pain.^80^

More recently, a study of non-specific neck pain investigating neuropathic components, and in particular neck pain-associated functional abnormalities related to sensory and sympathetic innervation demonstrated signs of functional impairment of innervation. These were reflected in changes in tactile sensitivity and vasoactive sympathetic function and may be based on both central and peripheral mechanisms.^81^ Of note, osteoarthritic pain does not change sensory or pain thresholds in individuals with neck pain.^82^

Another striking piece of evidence in support of a neuropathic component underlying neck pain is the findings of the CSM-Protect trial, the first adequately powered double-blind randomised controlled drug trial for DCM.^58^ Riluzole is an approved neuroprotective drug in clinical use for ALS. It has been linked to reducing glutamatergic excitotoxicity in neurons via a number of mechanisms.^83^ Although Riluzole treatment did not alter functional outcome in DCM, significant improvements in neck pain were detected.^58^

A neuropathic pain component in DCM is further supported by recent preclinical findings which echoed the findings of the clinical trial.^59^ Finally, it must not be overlooked that DCM is a form of spinal cord injury. The importance of neuropathic pain in Spinal Cord Injury (SCI) is well established.^84^

### Outcome assessment in DCM is a challenge for translational research and will be further evaluated

As outlined, the selection of VAS neck pain, and the mJOA is based on the current best available assessments. While the mJOA is a robust, and fully validated measure, this scale does not capture pain and has a reduced sensitivity to change in milder disease.^59^ Presently, there is no combined assessment tool of function and pain validated for DCM,^85^ with pain typically captured using VAS.^61 78^ RECODE-DCM, a parallel international consensus initiative is underway to determine the most suitable outcome measurements for DCM.^51^

This has led to two important considerations in the design of this trial: the selection of the inclusion criteria and of the trial endpoints.

The eligibility criteria were designed to ensure the most cost-efficient design and likelihood of success.^86^ The surgical treatment of mild DCM is controversial,^16^ and therefore risks under-representation in this trial if included. Additionally, surgical treatment alone is likely to return a maximum mJOA score in mild disease.^86^ Alongside the recognised plateau effect of higher mJOA scores, this therefore risks masking a treatment effect. To prevent these effects, only moderate/severe scores in the mJOA are included in the trial. Similarly, this is the concern for neurological comorbidities or previously treated myelopathy. The mJOA is a measure of functional disability and therefore neurological comorbidities may instead be measured.^85^ This is why other neurological comorbidities that could mask the symptoms of DCM are excluded from the trial. Based on experience from traumatic spinal cord injury,^87^ it is anticipated that the biological recovery capacity is altered in patients with previously treated myelopathy. Additionally, this subgroup has received relatively little research,^77^ and the data informing the surgical response and MCID are based on series which excluded repeat surgery.^57 88^ Previously treated myelopathy is under-researched, but the preclinical regenerative capacity is anticipated to be different, as are the surgical response and appropriate MCIDs. Patients who underwent surgery for DCM in the past are thus excluded.

In addition, a broad range of secondary endpoints have been included. These assessments have been selected to capture the far-ranging disability experienced by people with DCM. It includes the evaluation of promising objective, quantitative measures, such as microstructural MRI,^89^ respiratory physiology,^74 90^ GRASSP-Myelopathy (adapted from GRASSP^91^ and gait-laboratory analysis^92 93^). It also includes an assessment of carer quality of life for the first time in a DCM trial.^14^ It is recognised that these additional assessments increase the time requirements on participants and investigators, and therefore only a fraction are defined as per protocol. The identification and establishment of improved assessment measures would be of value to future trials and clinical practice.

## Summary

RECEDE-Myelopathy will evaluate the efficacy of Ibudilast, as adjuvant treatment, to improve recovery after surgical decompression in DCM. It is the first regenerative medicine trial in DCM, and the first DCM trial to directly target all the recovery priorities identified by sufferers.

### Ethical approval and dissemination

The RECEDE-Myelopathy trial protocol V.2, 11 March 2020, informed consent forms and all other relevant trial documents have been approved by Central London Research and Ethics Committee (REC), reference 20/LO/0185 (IRAS No: 213009). HRA approval from HRACW was received on 1 July 2020. Annual reports will be submitted to the REC in accordance with local national requirements. Trial will be performed following Good Clinical Practice (GCP) from the International Council for Harmonisation of Technical Requirements for Pharmaceuticals for Human Use (ICH) guidelines and the letter of the Declaration of Helsinki, as well as any other local regulatory requirements and laws.

All enrolled subjects will have the capacity to consent for the trial and can withdraw from the study at any point. Consent will be obtained by the research team and confirmation of consent to continue partaking in the study will be done on every trial visit.

### Dissemination of outcomes and findings from the study with patient involvement

We intend to involve patients with DCM in the dissemination of research output, both in the production of scientific and lay material, and its communication. Finally, we are currently evaluating the use of PPI representatives to communicate findings to professional audiences.

The results of the study will also be presented at international scientific conferences and in peer-reviewed journals regardless of the trial outcome.

Ownership of the data arising from this trial resides with the trial team. On completion of the trial the data will be analysed and tabulated and a Final Trial Report prepared.

We intend to disseminate the findings via peer-reviewed journals and presentations at national and international meetings. In addition to meetings orientated around neurosurgery, we will target conferences organised for the different health professionals who care for patients with DCM, including Neurology, Primary Care, Geriatrics and Rehabilitation medicine. We will publish the results of the trial on the EudraCT website.

Research findings will be disseminated to relevant service user groups and charities (including Myelopathy.org) through newsletters, website posts and public presentations. The dedicated trial website will also include dedicated pages for members of the public. We will present the trial in open days organised by hospitals participating in the trial where members of the public are invited to find out about on-going research.

Participants will be able to view global trial results on the trial website.

The trial partners, funders and sponsor will be acknowledged in the publication. Any scientific paper, presentation or communication concerning the trial shall be submitted to each relevant party following their guidelines.

We do not intend to distribute deidentified patient data at this point of time.

## Supplementary Material

Author's manuscript
