# Supplementary material for: Targeting patient recovery priorities in degenerative cervical myelopathy: design and rationale for the RECEDE-Myelopathy trial—study protocol
Source: BMJ Open. 2023 Mar 7;13(3):e061294. doi: 10.1136/bmjopen-2022-061294 (PMC10008337; doi:10.1136/bmjopen-2022-061294)
Supplement: Author's manuscript [file bmjopen-2022-061294.draft_revisions.pdf]

BMJ Open is committed to open peer review. As part of this commitment we make the peer review history of every article we publish publicly available.

When an article is published we post the peer reviewers' comments and the authors' responses online. We also post the versions of the paper that were used during peer review. These are the versions that the peer review comments apply to.

The versions of the paper that follow are the versions that were submitted during the peer review process. They are not the versions of record or the final published versions. They should not be cited or distributed as the published version of this manuscript.

BMJ Open is an open access journal and the full, final, typeset and author-corrected version of record of the manuscript is available on our site with no access controls, subscription charges or pay-per-view fees (http://bmjopen.bmj.com).

If you have any questions on BMJ Open's open peer review process please email info.bmjopen@bmj.com

BMJ Open

Targeting patient recovery priorities in degenerative cervical myelopathy: design and rationale for the RECEDE-Myelopathy trial

Journal:	*BMJ Open*
Manuscript ID	bmjopen-2022-061294
Article Type:	Protocol
Date Submitted by the Author:	10-Feb-2022
Complete List of Authors:	Davies, Benjamin; University of Cambridge, Department of Clinical Neurosurgery; University of Cambridge<br>Mowforth, Oliver; University of Cambridge, Department of Academic Neurosurgery<br>Yordanov, Stefan; Addenbrooke's Hospital, Neurosurgery<br>Alvarez-Berdugo, Daniel; Cambridge University Hospitals NHS Foundation Trust<br>Bond, Simon; Cambridge University Hospitals NHS Foundation Trust, Cambridge clinical trials unit<br>Nodale, Marianna; Cambridge University Hospitals NHS Foundation Trust<br>Kareclas, Paula; Cambridge University Hospitals NHS Foundation Trust, Cambridge Clinical Trials Unit<br>Whitehead, Lynne; Cambridge University Hospitals NHS Foundation Trust<br>Bishop, Jon; NIHR Surgical Reconstruction and Microbiology Research Centre, Medical Statistician<br>Chandran, Siddharthan; University of Cambridge, Clinical Neurosciences<br>Lamb, Sarah; University of Oxford<br>Bacon, Mark; International Spinal Research Trust<br>Papadopoulos, Marios; St George's Hospital, Department of Neurosurgery London, United Kingdom, Department of Neurosurgery<br>Starkey, Michelle; Myelopathy.org<br>Sadler, Iwan; Myelopathy.org<br>Smith, Lara; Myelopathy.org<br>Kalsi-Ryan, Sukhvinder; Toronto Rehabilitation Institute<br>Carpenter, Adrian; University of Cambridge, Department of Clinical Neurosciences<br>Trivedi, Rikin A.; Cambridge Univ Hosp Fdn Trust<br>Wilby, Martin; The Walton Centre NHS Foundation Trust, Neurosurgery<br>Choi, David; National Hospital for Neurology and Neurosurgery, Department of Neurosurgery<br>Wilkinson, Ian; Division of Experimental Medicine and Immunotherapeutics, Department of Medicine, University of Cambridge; Cambridge Clinical Trials Unit,<br>Fehlings, Michael G.; Toronto Western Hospital<br>Hutchinson, Peter; University of Cambridge, Academic Neurosurgery<br>Kotter, Mark R.N.; University of Cambridge, Department of Clinical Neurosurgery; University of Cambridge
Keywords:	NEUROSURGERY, Spine < ORTHOPAEDIC & TRAUMA SURGERY,

	Neurosurgery < SURGERY

SCHOLARONE™
Manuscripts

Targeting patient recovery priorities in degenerative cervical myelopathy: design and rationale for the RECEDE-Myelopathy trial

Benjamin M. Davies[1], Oliver D. Mowforth[1], Stefan Yordanov[1], Daniel Alvarez-Berdugo[2], Simon Bond[2], Marianna Nodale[2], Paula Kareclas[2], Lynne Whitehead[3], Jonathan Bishop[4], Siddharthan Chandran[5], Sarah E Lamb[6], Mark Bacon[7], Marios C. Papadopoulos[8], Michelle Starkey[9], Iwan Sadler[9], Lara Smith[9], Sukhivinder Kalsi-Ryan[10], Adrian Carpenter[11], Rikin Trivedi[1], Martin Wilby[12], David Choi[13], Ian Wilkinson[2,14], Michael G Fehlings[15], Peter Hutchinson[1], Mark R.N. Kotter[1,16]

1) Academic Neurosurgery Unit, Department of Clinical Neurosurgery, University of Cambridge, Cambridge, UK
2) Cambridge Clinical Trials Unit, Cambridge University Hospitals NHS Foundation Trust, Cambridge, UK
3) Pharmacy Department, Cambridge University Hospitals NHS Foundation Trust, Cambridge, UK
4) Birmingham Clinical Trials Unit, Institute of Applied Health Research, University of Birmingham, Birmingham, UK
5) Centre for Clinical Brain Sciences, University of Edinburgh, Edinburgh, UK
6) College of Medicine and Health, University of Exeter, UK
7) International Spinal Research Trust, London, UK
8) Academic Neurosurgery Unit, St. George's University of London, London, UK
9) Myelopathy.org, Cambridge, UK
10) Department of Physical Therapy, Krembil Neuroscience Spine Program, Toronto Western Hospital UHN, Toronto, Canada
11) Wolfson Brain Imaging Centre, Department of Clinical Neurosciences, University of Cambridge, Cambridge, UK
12) Department of Neurosurgery, The Walton Centre for Neurology and Neurosurgery NHS Trust, Liverpool, UK
13) Department of Neurosurgery, National Hospital for Neurology and Neurosurgery, University College London Hospitals NHS Foundation, London, UK
14) Division of Experimental Medicine and Immunotherapeutics, University of Cambridge, Cambridge, UK
15) Division of Neurosurgery, Department of Surgery, Toronto Western Hospital, Toronto, Canada
16) WT MRC Cambridge Stem Cell Institute, Anne McLaren Laboratory, University of Cambridge, UK

Key words: cervical; myelopathy; spondylosis; spondylotic; stenosis; disc herniation; ossification posterior longitudinal ligament; degeneration; disability; recovery; questionnaire

Word Count:

Acknowledgements: Research in the senior author's laboratory is supported by the Cambridge NIHR Brain Injury MedTech Cooperative. MRNK is supported by a NIHR Clinician Scientist Award.

For peer review only

Disclaimer: This report is independent research arising from a Clinician Scientist Award, CS-2015-15-023, supported by the National Institute for Health Research. The views expressed in this publication are those of the authors and not necessarily those of the NHS, the National Institute for Health Research or the Department of Health and Social Care.

Correspondence:
Dr. Mark R.N. Kotter
Department of Clinical Neurosciences
University of Cambridge
Mrk25@medschl.cam.ac.uk

For peer review only

Abstract

Study Design:

Clinical trial protocol

Objectives:

Degenerative cervical myelopathy (DCM) is a common and disabling condition of symptomatic cervical spinal cord compression secondary to degenerative changes in spinal structures leading to a mechanical stress injury of the spinal cord. RECEDE-Myelopathy aims to test the disease-modulating activity of the PDE3/4 inhibitor Ibudilast as an adjuvant to surgical decompression in DCM.

Methods

RECEDE-Myelopathy is a multi-centre, double-blind, randomised, placebo-controlled trial. Participants will be randomized to receive either 60-100mg Ibudilast or placebo starting within 10 weeks prior to surgery and continuing for 24 weeks after surgery for a maximum of 34 weeks. Adults with DCM, who have a modified Japanese Orthopaedic Association score (mJOA) 8-14 inclusive and are scheduled for their first decompressive surgery are eligible for inclusion. The co-primary endpoints are pain measured on a visual analogue scale and physical function measured by the mJOA score at 6 months after surgery. Clinical assessments will be undertaken pre-operatively, post-operatively and 3, 6 and 12 months after surgery. We hypothesize that adjuvant therapy with Ibudilast leads to a meaningful and additional improvement in either pain or function, as compared to standard routine care.

Conclusions

At present, surgery is the only effective treatment for DCM, existing neurological damage does not fully recover and people with DCM retain life-long disabilities with severe impact on quality of life. Novel treatments that promote recovery are desperately needed. RECEDE-Myelopathy is the first regenerative medicine trial for DCM and the first trial to target all the recovery priorities of people with DCM, including pain and limb function as primary endpoints.

For peer review only

Strengths and Limitations:

- **First Regenerative medicine trial for DCM assessing Ibudilast as an adjuvant to surgical decompression.**
- **Significant patient and public involvement in trial design and outcomes planning.**
- **The specific mechanism of action of Ibudilast is highly suited to address both functional outcome and neuropathic pain in DCM.**
- **Limitation is need of increased patient follow up and monitoring due to drug monitoring and assessments needed.**

Introduction

Here we present the study rationale and design of RECEDE Myelopathy (**Re**generation in **Ce**rvical **De**generative Myelopathy), the first regenerative medicine trial for degenerative cervical myelopathy (DCM), which aims to test disease-modulating activity of the PDE3/4 inhibitor Ibudilast as an adjuvant to surgical decompression.

DCM is a common and progressive condition with devastating impact on quality of life

DCM is the most common cause of spinal cord impairment world-wide,[1,2] with some estimates of the prevalence as high as 2% of adults.[2,3,4] It arises when arthritic or developmental changes in the cervical spine compress the spinal cord, causing a progressive slow-motion spinal cord injury.[5] As a degenerative pathology the incidence is expected to rise in an ageing population.[6,7]

The consequences of DCM are numerous, varied, and often progressive. Symptoms include pain, loss of dexterity, imbalance and frequent falls, incontinence and in extreme circumstances paralysis.[1,8,9,10,11] A recent comparative study found sufferers have amongst the worst quality of life scores of all chronic disease,[12,13] and this is likely to also negatively impact on their supporters.[14] The cost of DCM to society has not been measured yet, but it is likely to be significant. Consequently, improving recovery after surgery is a significant unmet need and there is strong evidence that surgical treatment for DCM is cost-effective.[15]

For peer review only

Surgery is the only evidence-based treatment for DCM

At present, the only effective treatment for DCM is surgery. Whilst surgery can stop disease progression, the existing damage does not fully recover[16] and people with DCM retain life-long disabilities with severe impact on quality of life.[12,13] Many remain unable to return to full time work and reliant on others for day-to-day activities.[17] Given the severe long-term consequences of DCM, treatment alternatives that promote recovery are desperately needed.

Phosphodiesterase 3 inhibition promotes functional recovery in preclinical DCM

The Mitogen-Activated Protein Kinases (MAPK) play a vital role in intracellular signalling.[18] In response to extracellular stimuli, such as neurotransmitters, inflammatory factors or stress conditions, this family of interconnected serine/threonine kinases coordinates a diverse range of intracellular processes, including cell differentiation, proliferation and apoptosis, inflammation and stress responses.[19] This signalling pathway and its modulation have therefore been linked to many diseases including cancer, asthma, stroke, multiple sclerosis and Alzheimer's dementia. More recently, preclinical studies, including our own, have demonstrated that its modulation via inhibition of a class of enzymes called phosphodiesterases (PDE), can improve functional recovery and reduce the perception of pain following damage to the central nervous system.[20,21,22]

PDEs hydrolyse the intracellular messenger cyclic AMP (cAMP).[20,23] This results in modulation of MAPK signalling.[24,25] Inhibition of PDE3 is particularly attractive in DCM as treatment with the selective PDE3 inhibitor cilostozol resulted in improved functional recovery in a rat model of DCM,[26] likely by improving latent ischemia.

Improvements following surgery are associated with axon sprouting, re-myelination, and immunomodulation

In DCM, tethering and compression of the spinal cord initiates a cascade of secondary injury events, including ischemia, inflammation and apoptosis that ultimately cause increased neurological deficits.[5,27,28] The partial reversal of symptoms after surgery highlights an inherent, albeit attenuated, regenerative capacity of the spinal cord.[16,29] This is echoed by post-mortem studies and our preclinical data, which indicate that neurological recovery following decompression is associated with axonal plasticity, re-myelination, and modulation of the immune response.[29,30,31] Enhancing axonal plasticity and re-myelination is therefore key to improving outcomes after DCM.[32]

For peer review only

Phosphodiesterase 4 inhibition can promote functional recovery and modulate pain in preclinical models

PDE4 is another isoform of phosphodiesterase inhibitors, which has demonstrated preclinical benefits on axon outgrowth[20] and remyelination.[22] The best characterised application of PDE4 inhibitors involves preclinical models of traumatic spinal cord injury using a drug called rolipram.[20,21] Unanimously, these have demonstrated that modulation of the PDE4 cascade is able to benefit recovery. In addition, our own work demonstrated that inhibition of PDE4 is able to stimulate the regenerative response of a CNS stem cell population termed oligodendrocyte progenitor cells and engage in re-myelination,[22] a process that has been observed in post-mortem spinal cords affected by DCM.[30]

PDE4 inhibition also has a role in modulating the perception of pain. Central to the development and maintenance of chronic pain syndromes is glial activation within the central nervous system, which enhances pain sensitivity via neuronal-glial interactions.[33] Modulation of MAPK via PDE4 inhibition has demonstrated a reduction in pain in several preclinical models.[34,35,36,37] Bao *et al*. (2011) found that PDE4 inhibition improved not just motor recovery but also resulted in a reduction in neuropathic pain in a rat model of spinal cord injury.[38] PDE4 inhibition also has an anti-inflammatory effect, increasing cAMP production in leukocytes and therefore reducing the release of tumour necrosis factor-alpha, a potent inflammatory mediator and peripheral pain stimulus.[39]

Ibudilast is a potent PDE4 inhibitor with an excellent human safety profile

The majority of preclinical studies described have used rolipram for PDE4 inhibition. Whilst rolipram is a potent and selective PDE4 inhibitor, experience from translational trials, most recently in multiple sclerosis (MS),[40] have demonstrated poor tolerability in humans due to significant nausea and vomiting. The MS trial had to be terminated due to a lack of efficacy and poor tolerability. Additionally, preclinical evidence has demonstrated a narrow therapeutic window, with potentially adverse neurological sequalae if missed.

An alternative is Ibudilast (MN-166).[23] Ibudilast is a potent PDE4 inhibitor, with additional PDE3 and 5 receptor activity. Modulation of PDE3 is also attractive in DCM as it led to improved function in a preclinical model of DCM.[26] Another attractive feature of Ibudilast is that it has been in clinical use for over 20 years for the treatment of asthma and post-stroke

For peer review only

dizziness, without tolerability issues.[41]

Ibudilast is currently under investigation for a number of other neurological conditions, including alcohol [NCT03489850] and methamphetamine [NCT01860807] addiction, glioblastoma [03782415], amyotrophic lateral sclerosis (ALS)[42] and multiple sclerosis[43] in a series of double blind, placebo randomised controlled trials.

For ALS, a single Phase I/II trial has been completed. Two ALS cohorts of early stage disease and advanced stage disease requiring ventilation, were randomised 1:1 to receive Ibudilast or placebo. Overall, the primary endpoint of safety and tolerability was met. In the early stage disease takers, Ibudilast was associated with a significant increase in survival, and delayed requirement for ventilation.[44] Treatment effects were linked to per-protocol adherence to therapy.[45] A Phase III trial is now planned.
For MS, two phase II trials have been completed. The first one evaluated relapsing remitting MS; whilst it did not prevent the development of new brain lesions, it slowed the progression of brain atrophy in a dose dependent fashion. The second one, a follow-up study in progressive MS, found that Ibudilast significantly slowed the progression of brain atrophy.[46]

Of note, typical daily dosing in these trials ranged from 60-100mg, which is greater than the currently licensed dosing of 10-20mg per day for routine clinical practice. Whilst trials confirmed overall tolerability and safety for use of Ibudilast in these doses in humans, findings do indicate a dose dependent relationship for gastro-intestinal side effects, such as nausea, and headaches and, in a minority of cases, this led to discontinuation of therapy by participants.

RECEDE-Myelopathy (Regeneration in Cervical Degenerative Myelopathy)
RECEDE-Myelopathy is a multi-centre, double-blind, randomised, placebo-controlled trial assessing the efficacy of Ibudilast as an adjuvant treatment to decompressive surgery for degenerative cervical myelopathy. The specific mechanism of action of Ibudilast is highly suited to address both functional outcome and neuropathic pain in DCM. Therefore, prompted by the direct involvement of people with DCM in designing the study, RECEDE Myelopathy has an infrequently used study design of two co-primary endpoints. It is designed and powered to detect response of patients to Ibudilast with regards to function or

pain, independently, as well as a response to both endpoints**.** We hypothesise that Ibudilast promotes functional outcome and reduces pain in surgically treated DCM.

Methods

Study design and objectives

RECEDE-Myelopathy (**Re**generation in **Ce**rvical **De**generative **Myelopathy)** is a multi-centre, double-blind, randomised, placebo-controlled trial assessing the efficacy of Ibudilast as an adjuvant treatment to decompressive surgery for degenerative cervical myelopathy. Participants will be randomized to receive either 60-100mg Ibudilast (interventional arm) or placebo (control arm) starting within 10 weeks prior to surgery and continuing for 24 weeks after surgery for a maximum of 34 weeks of treatment. Pre-operative treatment may leverage the effects of inhibition of PDE3, whilst post-operative treatment aims at regeneration-inducing effects outlined above. The primary objective will be to compare improvement in pain or physical function at 6 months after surgery between the two arms of the trial. We hypothesize that adjuvant therapy with Ibudilast leads to a meaningful and additional improvement in either pain or function, as compared to standard routine care (decompressive surgery).

***Patient and Public involvement (PPI)* - aligning research with patient priorities**

The involvement of public and patients representatives in research is recognised to be of key importance to ensure it delivers meaningful, practice-changing information.[63,64,65,66] As with many fields, this has been a problem for DCM.[56,67,68] To address this issue, we founded Myelopathy.org, the first and so far only charity for people with DCM. Whilst in its infancy, the platform has become an international focus for people with DCM, hosting a peer-to-peer

For peer review only - http://bmjopen.bmj.com/site/about/guidelines.xhtml

support community (Myelopathy Support) of over 2000 users.[69] This has enabled larger scale insights into the perspective of individuals with DCM[17,70,71,] and ultimately led to RECODE-DCM, a James Lind Alliance-led initiative to identify and define the research priorities for DCM.[56] (https://aospine.aofoundation.org/research/recode-dcm)

Definition of recovery priorities for people with DCM

As part of RECODE DCM, a focus group of people with DCM was created with the objective to develop recovery domains. These were subsequently prioritised via an international, online survey (n = 485).[10] In contrast to the research focus to date,[67,53] pain emerged as the number one recovery priority, closely followed by hand, and walking function. Consequently, the development of adjuvant treatments for DCM should be most usefully focussed on reducing pain and improving limb function.

Dissemination of outcomes and findings from the study with patient involvement

We intend to involve Patients with DCM in the dissemination of research output, both in the production of scientific and lay material, and its communication. Finally, we are currently evaluating the use of PPI representatives to communicate findings to professional audiences.

Patient screening and eligibility

A summary of the study flow diagram, including full inclusion and exclusion criteria, is presented in Figure 1. In summary, adults (age 18-80) with a diagnosis of DCM (participants must have at least one MRI indicator, clinical symptom, and neurological sign from Table 1 to be eligible for inclusion) and a disease severity of modified Japanese Orthopaedic Association scale (mJOA) 8-14 inclusive, scheduled for their first decompressive surgery, will be approached to consider participation in RECEDE-Myelopathy.

Table 1: Trial criteria for diagnosis of DCM. Participants must have at least one MRI indicator, clinical Symptom, and neurological sign to be eligible for inclusion.

MRI Indicators	Clinical Symptoms	Neurological Signs
Effacement of CSF and deformation of cord	Numb hands	Pyramidal weakness
T1 signal change	Clumsy hands	Hyperreflexia
T2 Signal change	Bilateral arm paraesthesia	Positive Hoffman sign
Segmentation of T2 signal change	Gait impairment	Upgoing plantar response

Reduction in transverse area of cord	Lhermitte's phenomenon	Atrophy of intrinsic hand muscles
	Weakness	Spasticity/clonus
		Broad based, unstable gait

Eligibility will be further assessed against exclusion criteria, largely dictated by safety requirements for use of Ibudilast, and in precluding masking of treatment effects. This includes, concomitant lumbar canal stenosis or other neurological condition, presentation with symptoms due to trauma (e.g., central cord syndrome), a history of allergy to Ibudilast, any of its formulation or that of the placebo, pregnancy, unwillingness to use reliable contraception, active malignancy, liver impairment or thrombocytopaenia. The latter will be assessed via serum biochemistry and haematological assessment.

Enrolment and randomisation

Those patients who satisfy the screening criteria and agree to study participation are enrolled and randomized at 1:1 to one of the two treatment arms. A web-based randomisation system (Sealed envelope) performing stratified blocked randomisation will be used stratifying by baseline mJOA (<12 vs. >=12), age (<60 years vs. >=60 years) and time to onset of the disease (>6 months vs. <=6 months); random block size will be used. Throughout randomisation and follow-up, the subjects, physicians, and data collectors remain blinded to group allocation.

Treatment description and dosage modification

The investigational medicinal product (IMP) is a 24-34 week course of Ibudilast or matched placebo in an escalating dosage regimen up to a maximum of 100mg daily if tolerated. The escalating dosage regimen is to minimise gastrointestinal side effects. Ibudilast is available in 10mg capsules, and therefore the IMP will be provided as such. The placebo is identical in shape, size, and color to the Ibudilast capsule, and participants will be provided with the same instructions.

Participants will start treatment within 10 weeks prior to surgical decompression and will continue taking drug for up to 24 weeks post-surgery. The excretion half-life of Ibudilast is approximately 20 hours. The IMP will be taken in divided doses, twice daily, morning and

For peer review only

evening, for a maximum of 34 weeks. Because this is the first surgical trial with Ibudilast, and to mitigate any potential interference on the coagulation system, treatment will be halted 5 days prior to surgery and resumed at the previous maximum dose right after operation.

Ibudilast is associated with gastro-intestinal side effects, such as nausea and dyspepsia. Alongside dose escalation, participants will be instructed to take trial medication with food or within an hour of eating to improve gastrointestinal tolerability. In the event of minor gastrointestinal complaints, participants will be offered symptomatic treatment in the first instance, in conjugation with ongoing IMP therapy. If this is unsuccessful, or not agreeable to participants, the trial therapy will be decreased in decrements of 20mg every 5 days, until a tolerable dosing level is achieved, or the drug is stopped. If a participant cannot tolerate a minimum daily dosage of 60mg despite additional supportive measures, treatment within the trial will be stopped.

Surgery

There are a number of different approaches used to decompress the spinal cord in DCM. No surgical approach has been shown to be superior, and the consensus is that the approach needs to be tailored to the specific anatomy. The surgical care of participants will therefore be at the discretion of the treating clinician and not protocolised.

Outcome measures and follow up

Two patient-informed co-primary endpoints: pain and function

Inhibiting phosphodiesterases 3 and 4 with Ibudilast has the potential to benefit both pain and functional recovery by promoting repair mechanisms in the spinal cord as well as exerting neuroprotective effects. This provides a unique opportunity to address the most important recovery priorities identified by individuals with myelopathy. Therefore, RECEDE-Myelopathy has two outcome targets: pain and physical function.[10] These co-primary endpoints will be assessed at 6 months after surgery, a time point when the majority of recovery will have been achieved.[47]

The study is thus powered to detect meaningful changes with regards to the co-primary endpoints independently from each other, i.e., it is designed to establish whether Ibudilast has beneficial effects on function or pain alone or whether it beneficially modulates both end points

For peer review only

Co-primary endpoint 1. The international standard, and most validated measure for assessment of function in DCM, is the mJOA scale.[16,48,49] The mJOA is a composite score of upper and lower limb muscular function, upper limb sensory function and bladder function.

Co-primary endpoint 2. Pain has been identified as the recovery priority of DCM patients. The most common form is neck pain,[9] with a neuropathic component that is responsive to neuroprotective treatments.[50,51]

Whilst numerous tools have been developed for the measurement of pain,[52] the Initiative on Methods, Measurements and Pain Assessment in Clinical Trials (IMMPACT) agree that pain intensity scales provide the most relevant outcome measure for demonstrating efficacy. In DCM the visual analogue scale (VAS) is the most popular example of this.[53] Although not exclusively validated for DCM, the psychometric properties of VAS neck and VAS arm pain have been evaluated in degenerative disease of the cervical spine,[54,55] with VAS neck pain having better repeatability.

This design will address the most important priorities of people with DCM.[10] It leverages the mechanism of action of Ibudilast to maximise the chances of demonstrating the benefit of the studied intervention. It will increase the knowledge that can be gained through the study and demonstrate whether the proposed mechanisms of neuroprotection and regeneration can be applied to promote function and/or reduce pain. Finally, the dual end-point design will make the study more efficient than conducting two independent trials. The chosen two endpoint design will hence increase the value of the study.

Secondary and exploratory endpoints

Clinical assessments will additionally be undertaken pre-operatively, post-operatively and 3, 6 and 12 months after surgery. The disability reported in the context of DCM is wide ranging. In the absence of a consensus dataset,[56] an issue that we are currently attending to as part of RECODE-DCM, a variety of clinician administered and patient reported outcome measures will be used to provide a comprehensive assessment. A full list of assessments and their time-points is presented in **Error! Reference source not found.**.

For peer review only

Table 2: Schedule of Assessments

Assessments	Screening visit and initial assessments	Randomisation	Start of IMP (within 2 -3 month prior to Surgery)	Pre-Operative assessments (within 21 days prior to surgery)	Surgery	Post-operatively/ Discharge (within 14 days post-surgery)	3- months Post Operatively (±21 days)	6-months Post Operatively (±21 days)	12-months Post Operatively (±21 days)
Informed consent	X								
Eligibility Assessment	X								
Demographics	X								
Medical history & DCM characteristics	X								
Concomitant medication	X			X			X	X	X
Blood Tests (FBC, LFT, E/U/C, TFTs)	X			X			X	X	X
ECG	X								
Urine analysis	X								
Pregnancy test	X								
Randomisation		X							
Neurological examination	X			X		X	X	X	X
mJOA	X			X			X	X	X
30m Walk test	X			X			X	X	X
GRASSP-Cervical Myelopathy	O			O			O	O	O
SCIMv3	O			O				O	
WHO performance status				X					
Neck Disability Index	O			O		O	O	O	O
VAS Pain	X			X		X	X	X	X
SF-36	X			X			X	X	X
EQ5D / Health Resource Usage	X			X			X	X	X
Quick-DASH	O			O			O	O	O
Carer QoL (sub-study)	X			X			X	X	X
Review of AEs			X	X		X	X	X	X
Dosing Diary	X								
Dispensing of IMP			X			X	X		
Serum sample for PK studies	X			X	X		X	X	X
Compliance Assessment				X		X	X	X	
IMP review				X		X	X	X	
Respiratory Physiology & muscle function				X				X	
MRI				X				X	
Gait Lab (sub-study)				X			O	X	
Surgery details					X				
Surgery complications						X	X	X	X
Hospital discharge						X			
CSF sample					O				

Not all assessments will be conducted at every time point, or be mandated, to reduce participant and investigator burden. Assessment is also extended to carers of participants. Building on our preliminary finding of reduced quality of life amongst DCM carers,[14] the Care Quality of Life instrument (CarerQol) will be used to evaluate this.[57]

Adaptive sample size design

The minimum clinically important difference (MCID) for the mJOA is estimated to be between 1 and 2 points.[58] Although not exclusively validated for DCM, the MCID for VAS neck and VAS arm pain has been calculated for degenerative disease of the cervical spine with values ranging from 8-26mm.[54,55] Both VAS pain and mJOA improve more than the

MCID with surgery alone,[47] and the amount of change is linked to the pre-operative baseline.[58] Consequently, in consensus with patients we have determined the MCID of the VAS pain score as being 1cm and for the mJOA 1 point. This has been modelled to ensure statistical power across all baseline scenarios.

On this basis, a total sample size of 362 participants under equal randomisation will provide 85% power to detect a difference of 1 between treatment arms on the mJOA scale (assuming a standard deviation (SD) of 2.89), using a two-sided t-test at a 2.5% significance level to adjust for multiple comparisons.[59] The trial is also powered to detect a similar difference on the VAS neck pain scale (assuming a difference of 1 and a SD of 2.88).

A blinded interim analysis will be conducted to refine the power calculation. The aim will be to reassess the sample size in time to allow any potential extension and increase in sample size to be put into effect. Reduction in sample size will not be permitted. Any sample size increase will be based on checking the assumption regarding the SD, and will not estimate any treatment effect, hence no subsequent adjustment to future analyses is needed.
Under such a framework, the theoretical optimal time to schedule such an interim analysis would be just as the last patient is recruited under the original sample size (n=362) following which a decision could be taken to either halt or extend recruitment. However, for reasons of practicality a window for the interim analysis will be up to a period of 4 months before reaching the total sample size.

The SD and correlation of both endpoints will be reassessed using data pooled across the arms. The three possible statistically significant conclusions of the formal hypothesis testing (VAS; mJOA; both) will be provided with revised target sample sizes needed to achieve 85% power under the same MCID values, but with revised estimates for the SD values and correlation. A recommended revised sample size will be the smallest of the three new target sample sizes or the original sample size if this is larger; hence the recommended sample size will never be a reduction from the original.

The next step of the interim analysis will be to calculate the conditional power of the three possible positive outcomes based on, the estimated unblinded treatment effects from the current data, plus, the distribution of future data from the revised sample size under the corresponding combinations of true treatment effects (MCID or zero), and SD and correlation

For peer review only

estimates from the first step. If all three conditional power values are less than 30% then the recommendation would be to halt the study.

Trial monitoring

All data collected during the trial will be recorded into a Case Report Form (CRF), which will be labelled using a participant's unique trial ID and date of birth. CRFs will be completed by the local research team and copies will be sent to trial coordination centre, where it will be entered into a central digital database. Safety assessments will be conducted by local investigators and reported and handled according to a predefined trial protocol. This includes a mechanism to capture surgical complications.[60] The Trial Steering Committee (TSC) will provide overall supervision with respect to the conduct of the trial. The TSC will consist of an independent Chairperson (Prof Michael Fehlings), a PPI representative (Mr Iwan Sadler), independent clinical and science experts (Prof Marios Papadopoulos and Dr Mark Bacon), clinical pharmacology and neurosurgery experts (Prof Ian Wilkinson and Prof Peter Hutchinson), the Chief Investigator and members of the Trial Management Group (e.g., trial statistician, trial manager). The ethical and safety aspects of the trial will be overseen by an independent Data Monitoring Committee (DMC) who will meet once a year and their meetings will be timed so that reports can be fed into the TSC meetings. Safety assessment will be performed for every participant since consent and until end of their participation in the trial. To date, there are no known expected serious adverse reactions (SAR) for Ibudilast, and thus any reported SAR will be considered a suspected unexpected serious adverse reaction (SUSAR). Furthermore, surgical complications will be followed up as events of special interest to be reviewed by the DMC.

Statistical methods

The primary endpoint and key secondary endpoints are all measured on a continuous scale. A comparison of mean values between treatment arms, adjusting for baseline covariates, will be provided using linear regression. Estimates, standard errors, 95% confidence intervals and p-values will be provided.

For formal hypothesis testing, a closed testing approach will be used to deal with multiple endpoints.[61] Initially either of the co-primary endpoints (mJOA or VAS neck pain) may test a null hypothesis of zero mean difference at a 2-sided 2.5% significance level,[62] with the

For peer review only

remaining primary endpoint tested at 5% significance level. This will enable us to determine whether the study drug is effective on pain or function independently.

Subsequently a gate-keeping approach will be used where an endpoint below the primary endpoint in the pre-specified ordering is only tested if all the preceding endpoints reject the null hypothesis, using the nominal p-value. If an endpoint does not reject the null, then all endpoints below it have the same conclusion-not rejecting the null-regardless of their nominal p-value. The ordering is, after primary endpoints, SF-36 PCS and then SF-36 MCS.

Secondary endpoints will be compared between treatment arms using approach regression techniques: linear regression for continuous endpoints, logistic regression for binary endpoints, and Cox regression for time-to-event.

The following baseline covariates, in addition to the baseline value of the endpoint, will be used to adjust all comparisons

- Time to onset
- Smoking status (yes/no)
- Age
- Psychiatric comorbidities (yes/no)
- Impaired gait (yes/no)

A detailed statistical analysis plan will be produced before the final database lock.

Discussion

This is the first regenerative medicine trial for DCM. It is also the first trial to target all the recovery priorities for people with DCM, namely pain and upper and lower limb function as primary endpoints.[10] This is significant, as in the recent evaluation of Riluzole as a perioperative neuroprotective therapy in DCM, whilst the primary end-point (1-point change in mJOA) was not met, VAS neck pain, a secondary end-point, improved significantly.[50] However, as a secondary endpoint the causal link can only be tentative.

RECEDE-Myelopathy addresses 5 of the 10 top priorities identified by RECODE-DCM

Priority 1 - Raising awareness[1,72]:

For peer review only

- RECEDE-Myelopathy is the first regenerative medicine trial for DCM. It is the second powered DCM CTIMP world-wide. We will seek to leverage this fact to attract attention to DCM by optimising communication before, during and after the trial, aiming at maximising our audience, to include patient organisations, a wide range of health care providers and the scientific community. We also aim to break into non-specialist mainstream media.

Priority 2 – Assessment and monitoring:

- RECEDE-Myelopathy will help to standardise assessment and monitoring across study centres, and thus promote the implementation of the recent international guidelines.[16] Additionally, a number of new secondary endpoints are included for the first time in a clinical trial of DCM, including gait[73] and respiratory physiology.[74]

Priority 5 – Developing a better understanding of the pathophysiology of DCM[75]

- RECEDE-Myelopathy tests the hypothesis that MAPK signalling mediated by PDE3/4 can promote recovery after DCM. It will serve as a platform for sub-studies, including imaging studies, and molecular biology studies on blood draws and CSF.

Priority 6 – Rehabilitation:

- There are no evidence-based measures to promote rehabilitation in DCM.[76] RECEDE-Myelopathy will investigate a drug that has the possibility of improving functional outcomes in DCM.

Priority 7 – Novel therapies:

- At present, surgery is the only possible treatment for DCM. If successful, RECEDE-Myelopathy will pave the way for the first evidence-based non-surgical adjuvant treatment.

Neuropathic origins of neck pain in DCM

Pain has been identified as the recovery priority of people with DCM.[10] Where assessed, previous trials have focused on neck (or axial) pain and arm pain.[53,48,77] Our findings in a survey of 230 patients, found neck pain was the most commonly reported first symptom of DCM (13%), and with respect to pain, twice as common as limb pain (7%). Moreover, overall neck pain was experienced more often (80%) than arm pain (70%) (56). In addition, individuals can be affected by atypical pain syndromes such as headache.[8,78,11]

For peer review only

Counter to the prevalent belief that neck pain is mainly caused by arthritic changes to the spine, an emerging literature points to a neuropathic origin. First, arthritic changes are omnipresent with progressive age, causing increasing levels of cord compression.[3,9] In many instances this does not lead to neck pain, even in the context of DCM.

A neuropathic component of chronic neck pain has long been postulated. For example, a psychophysical study measuring responses to electro-cutaneous stimulation in subjects with chronic neck pain found evidence of secondary hyperalgesia which, in turn, implies central sensitisation of nociceptive pathways.[79] The results were compatible with studies which identify potential anatomical origins of chronic neck pain but provide evidence that central sensitisation may be the relevant mechanism of pain production.

A single centre study investigated the relation between pain provoking cervical segments identified by diagnostic dorsal root blockades and elevation of quantitative sensory testing of the cervical dermatomes using Semmes-Weinstein monofilaments in patients suffering from neck pain but not radiculopathy. This revealed a systematic elevation of detection thresholds, an adaptation in contrast with, but not contradictory to, central sensitization of high threshold neurons in chronic pain.[80]

More recently, a study of non-specific neck pain investigating neuropathic components, and in particular neck pain-associated functional abnormalities related to sensory and sympathetic innervation demonstrated signs of functional impairment of innervation. These were reflected in changes in tactile sensitivity and vasoactive sympathetic function and may be based on both central and peripheral mechanisms.[81] Of note, osteoarthritic pain does not change sensory or pain thresholds in individuals with neck pain.[82]

Another striking piece of evidence in support of a neuropathic component underlying neck pain are the findings of the CSM-Protect trial, the first adequately powered double blind randomised controlled drug trial for DCM.[50] Riluzole is an approved neuroprotective drug in clinical use for amyotrophic lateral sclerosis. It has been linked to reducing glutamatergic excitotoxicity in neurons via a number of mechanisms.[83] Although Riluzole treatment did not alter functional outcome in DCM, significant improvements in neck pain were detected.[50]

For peer review only

A neuropathic pain component in DCM is further supported by recent preclinical findings which echoed the findings of the clinical trial.[51] Finally, it must not be overlooked that DCM is a form of spinal cord injury. The importance of neuropathic pain in SCI is well established.[84]

Outcome assessment in DCM is a challenge for translational research and will be further evaluated.

As outlined, the selection of VAS neck pain, and the mJOA is based on the current best available assessments. Whilst the mJOA is a robust, and fully validated measure, this scale does not capture pain and has a reduced sensitivity to change in milder disease.[49] Presently, there is no combined assessment tool of function and pain validated for DCM,[85] with pain typically captured using visual analogue scales.[53,77] RECODE-DCM, a parallel international consensus initiative is underway to determine the most suitable outcome measurements for DCM.[56]

This has led to two important considerations in the design of this trial: the selection of the inclusion criteria and of the trial endpoints.

The eligibility criteria were designed to ensure the most cost-efficient design and likelihood of success.[85] The surgical treatment of mild DCM is controversial,[16] and therefore risks underrepresentation in this trial if included. Additionally, surgical treatment alone is likely to return a maximum mJOA score in mild disease.[86] Alongside the recognised plateau effect of higher mJOA scores, this therefore risks masking a treatment effect. To prevent these effects, only moderate/severe scores in the mJOA are included in the trial. Similarly, this is the concern for neurological comorbidities or previously treated myelopathy. The mJOA is a measure of functional disability and therefore neurological comorbidities may instead be measured.[85] This is why other neurological comorbidities that could mask the symptoms of DCM are excluded from the trial. Based on experience from traumatic spinal cord injury,[87] it is anticipated that the biological recovery capacity is altered in patients with previously treated myelopathy. Additionally, this subgroup has received relatively little research,[77] and the data informing the surgical response and MCID is based on series which excluded repeat surgery.[47,88] Previously treated myelopathy is under-researched, but the pre-clinical regenerative capacity is anticipated to be different, as are the surgical response and appropriate MCIDs. Patients who underwent surgery for DCM in the past are thus excluded.

In addition, a broad range of secondary endpoints have been included. These assessments have been selected to capture the far-ranging disability experienced by people with DCM. It includes the evaluation of promising objective, quantitative measures, such as microstructural MRI,[89] respiratory physiology,[74,90] GRASSP-Myelopathy (adapted from GRASSP[91]) and gait-laboratory analysis.[92,93] It also includes an assessment of carer quality of life for the first time in a DCM trial.[14] It is recognised that these additional assessments increase the time requirements on participants and investigators, and therefore only a fraction are defined as per protocol. The identification and establishment of improved assessment measures would be of value to future trials and clinical practice.

Conclusion

RECEDE-Myelopathy will evaluate the efficacy of Ibudilast, as adjuvant treatment, to improve recovery after surgical decompression in DCM. It is the first regenerative medicine trial in DCM, and the first DCM trial to directly target all the recovery priorities identified by sufferers.

Ethical approval

The RECEDE-Myelopathy trial protocol version 2, 11 March 2020, informed consent forms and all other relevant trial documents have been approved by Central London Research and Ethics Committee (REC), reference 20/LO/0185. Annual reports will be submitted to the REC in accordance with local national requirements. Trial will be performed following GCP from the ICH guidelines and the letter of the Declaration of Helsinki, as well as any other local regulatory requirements and laws.

For peer review only

Footnotes:

Contributors:

The idea and project were conceived by MRK and BD. The initial draft of the manuscript was created by BD, OM, and circulated amongst other authors for critical revisions. The Chief investigator for RECEDE is MRK and co-investigator is BD.

All authors approved the final version of the manuscript.

Competing Interest:

MRK holds a research grant from clinical scientist award and has support for the study from Medicinova. MCP holds research grant award with the NIHR. BD holds research grants with NIHR HTA POLYFIX-DCM, Evelyn Trust (DCM – COINs) and award from National Lottary UK for developing a peer-to-peer support community for Degenerative Cervical myelopathy.BD is also a founder of MoveMed LTD. PJAH holds NIHR research grants.

Funding

Clinician Scientist Award, CS-2015-15-023, supported by the National Institute for Health Research

Ethics Approval:

IRAS No:213009 Ethics Committee : London Central Research Ethics Committee.

For peer review only

References

1. Davies BM, Mowforth OD, Smith EK, Kotter MR. Degenerative cervical myelopathy. *BMJ*. 2018;360:k186. doi:10.1136/bmj.k186

2. Kadanka Z, Adamova B, Kerkovsky M, et al. Predictors of symptomatic myelopathy in degenerative cervical spinal cord compression. *Brain Behav*. 2017;7(9). doi:10.1002/brb3.797

3. Kovalova I, Kerkovsky M, Kadanka Z, et al. Prevalence and Imaging Characteristics of Nonmyelopathic and Myelopathic Spondylotic Cervical Cord Compression. *Spine*. 2016;41(24):1908-1916. doi:10.1097/BRS.0000000000001842

4. Smith SS, Stewart ME, Davies BM, Kotter MRN. The Prevalence of Asymptomatic and Symptomatic Spinal Cord Compression on Magnetic Resonance Imaging: A Systematic Review and Meta-analysis. *Global Spine J*. 2021;11(4):597-607. doi:10.1177/2192568220934496

5. Akter F, Kotter M. Pathobiology of Degenerative Cervical Myelopathy. *Neurosurg Clin N Am*. 2018;29(1):13-19. doi:10.1016/j.nec.2017.09.015

6. Nouri A, Tetreault L, Singh A, Karadimas SK, Fehlings MG. Degenerative Cervical Myelopathy: Epidemiology, Genetics, and Pathogenesis. *Spine*. 2015;40(12):E675-693. doi:10.1097/BRS.0000000000000913

7. Grodzinski B, Durham R, Mowforth O, Stubbs D, Kotter MRN, Davies BM. The effect of ageing on presentation, management and outcomes in degenerative cervical myelopathy: a systematic review. *Age Ageing*. Published online November 19, 2020. doi:10.1093/ageing/afaa236

8. Davies BM, Munro CF, Kotter MR. A Novel Insight Into the Challenges of Diagnosing Degenerative Cervical Myelopathy Using Web-Based Symptom Checkers. *J Med Internet Res*. 2019;21(1):e10868. doi:10.2196/10868

9. Boerger T, Alsouhibani A, Mowforth O, et al. Moving Beyond the Neck and Arm: The Pain Experience of People With Degenerative Cervical Myelopathy Who Have Pain. *Global Spine J*. Published online February 25, 2021:2192568220986143. doi:10.1177/2192568220986143

10. Davies B, Mowforth O, Sadler I, et al. Recovery priorities in degenerative cervical myelopathy: a cross-sectional survey of an international, online community of patients. *BMJ Open*. 2019;9(10):e031486. doi:10.1136/bmjopen-2019-031486

11. Mowforth OD, Davies BM, Kotter MR. “I am not delusional!” Sensory dysaesthesia secondary to degenerative cervical myelopathy. *BMJ Case Rep*. 2019;12(4). doi:10.1136/bcr-2018-229033

12. Oh T, Lafage R, Lafage V, et al. Comparing Quality of Life in Cervical Spondylotic Myelopathy with Other Chronic Debilitating Diseases Using the SF-36 Survey. *World Neurosurg*. Published online January 5, 2017. doi:10.1016/j.wneu.2016.12.124

For peer review only

13. Hirayama Y, Mowforth OD, Davies BM, Kotter MRN. Determinants of quality of life in degenerative cervical myelopathy: a systematic review. *Br J Neurosurg*. Published online November 18, 2021:1-11. doi:10.1080/02688697.2021.1999390

14. Mowforth OD, Davies BM, Kotter MR. Quality of Life Among Informal Caregivers of Patients With Degenerative Cervical Myelopathy: Cross-Sectional Questionnaire Study. *Interact J Med Res*. 2019;8(4):e12381. doi:10.2196/12381

15. Witiw CD, Tetreault LA, Smieliauskas F, Kopjar B, Massicotte EM, Fehlings MG. Surgery for degenerative cervical myelopathy: a patient-centered quality of life and health economic evaluation. *Spine J*. 2017;17(1):15-25. doi:10.1016/j.spinee.2016.10.015

16. Fehlings MG, Tetreault LA, Riew KD, et al. A Clinical Practice Guideline for the Management of Patients With Degenerative Cervical Myelopathy: Recommendations for Patients With Mild, Moderate, and Severe Disease and Nonmyelopathic Patients With Evidence of Cord Compression. *Global Spine Journal*. 2017;7(3_suppl):70S-83S. doi:10.1177/2192568217701914

17. Pope DH, Mowforth OD, Davies BM, Kotter MRN. Diagnostic Delays Lead To Greater Disability In Degenerative Cervical Myelopathy and Represent A Health-Inequality. *Spine*. Published online October 28, 2019. doi:10.1097/BRS.0000000000003305

18. Sun J, Nan G. The Mitogen-Activated Protein Kinase (MAPK) Signaling Pathway as a Discovery Target in Stroke. *J Mol Neurosci*. 2016;59(1):90-98. doi:10.1007/s12031-016-0717-8

19. Lu M, Wang Y, Zhan X. The MAPK Pathway-Based Drug Therapeutic Targets in Pituitary Adenomas. *Front Endocrinol (Lausanne)*. 2019;10:330. doi:10.3389/fendo.2019.00330

20. Nikulina E, Tidwell JL, Dai HN, Bregman BS, Filbin MT. The phosphodiesterase inhibitor rolipram delivered after a spinal cord lesion promotes axonal regeneration and functional recovery. *Proc Natl Acad Sci U S A*. 2004;101(23):8786-8790. doi:10.1073/pnas.0402595101

21. Pearse DD, Pereira FC, Marcillo AE, et al. cAMP and Schwann cells promote axonal growth and functional recovery after spinal cord injury. *Nat Med*. 2004;10(6):610-616. doi:10.1038/nm1056

22. Syed YA, Baer A, Hofer MP, et al. Inhibition of phosphodiesterase-4 promotes oligodendrocyte precursor cell differentiation and enhances CNS remyelination. *EMBO Mol Med*. 2013;5(12):1918-1934. doi:10.1002/emmm.201303123

23. Gibson LCD, Hastings SF, McPhee I, et al. The inhibitory profile of Ibudilast against the human phosphodiesterase enzyme family. *Eur J Pharmacol*. 2006;538(1-3):39-42. doi:10.1016/j.ejphar.2006.02.053

24. Smith FD, Langeberg LK, Cellurale C, et al. AKAP-Lbc enhances cyclic AMP control of the ERK1/2 cascade. *Nat Cell Biol*. 2010;12(12):1242-1249. doi:10.1038/ncb2130

25. Cuadrado A, Nebreda AR. Mechanisms and functions of p38 MAPK signalling. *Biochem J*. 2010;429(3):403-417. doi:10.1042/BJ20100323

For peer review only

26. Yamamoto S, Kurokawa R, Kim P. Cilostazol, a selective Type III phosphodiesterase inhibitor: prevention of cervical myelopathy in a rat chronic compression model. *J Neurosurg Spine*. 2014;20(1):93-101. doi:10.3171/2013.9.SPINE121136

27. Badhiwala JH, Ahuja CS, Akbar MA, et al. Degenerative cervical myelopathy - update and future directions. *Nat Rev Neurol*. 2020;16(2):108-124. doi:10.1038/s41582-019-0303-0

28. Akter F, Yu X, Qin X, et al. The Pathophysiology of Degenerative Cervical Myelopathy and the Physiology of Recovery Following Decompression. *Front Neurosci*. 2020;14:138. doi:10.3389/fnins.2020.00138

29. Dhillon RS, Parker J, Syed YA, et al. Axonal plasticity underpins the functional recovery following surgical decompression in a rat model of cervical spondylotic myelopathy. *Acta Neuropathol Commun*. 2016;4(1):89. doi:10.1186/s40478-016-0359-7

30. Ito T, Oyanagi K, Takahashi H, Takahashi HE, Ikuta F. Cervical spondylotic myelopathy. Clinicopathologic study on the progression pattern and thin myelinated fibers of the lesions of seven patients examined during complete autopsy. *Spine (Phila Pa 1976)*. 1996;21(7):827-833. doi:10.1097/00007632-199604010-00010

31. Karadimas SK, Moon ES, Yu WR, et al. A novel experimental model of cervical spondylotic myelopathy (CSM) to facilitate translational research. *Neurobiol Dis*. 2013;54:43-58. doi:10.1016/j.nbd.2013.02.013

32. Karadimas SK, Gatzounis G, Fehlings MG. Pathobiology of cervical spondylotic myelopathy. *Eur Spine J*. 2015;24 Suppl 2:132-138. doi:10.1007/s00586-014-3264-4

33. Ji RR, Berta T, Nedergaard M. Glia and pain: is chronic pain a gliopathy? *Pain*. 2013;154 Suppl 1:S10-S28. doi:10.1016/j.pain.2013.06.022

34. Kim HK, Hwang SH, Oh E, Abdi S. Rolipram, a Selective Phosphodiesterase 4 Inhibitor, Ameliorates Mechanical Hyperalgesia in a Rat Model of Chemotherapy-Induced Neuropathic Pain through Inhibition of Inflammatory Cytokines in the Dorsal Root Ganglion. *Front Pharmacol*. 2017;8:885. doi:10.3389/fphar.2017.00885

35. Kim HK, Kwon JY, Yoo C, Abdi S. The Analgesic Effect of Rolipram, a Phosphodiesterase 4 Inhibitor, on Chemotherapy-Induced Neuropathic Pain in Rats. *Anesth Analg*. 2015;121(3):822-828. doi:10.1213/ANE.0000000000000853

36. Kostich W, Hamman BD, Li YW, et al. Inhibition of AAK1 Kinase as a Novel Therapeutic Approach to Treat Neuropathic Pain. *J Pharmacol Exp Ther*. 2016;358(3):371-386. doi:10.1124/jpet.116.235333

37. Espírito-Santo RF, Meira CS, Costa RDS, et al. The anti-inflammatory and immunomodulatory potential of braylin: Pharmacological properties and mechanisms by in silico, in vitro and in vivo approaches. *PLoS One*. 2017;12(6):e0179174. doi:10.1371/journal.pone.0179174

38. Bao F, Fleming JC, Golshani R, et al. A selective phosphodiesterase-4 inhibitor reduces leukocyte infiltration, oxidative processes, and tissue damage after spinal cord injury. *J Neurotrauma*. 2011;28(6):1035-1049. doi:10.1089/neu.2010.1575

For peer review only

39. Francischi JN, Yokoro CM, Poole S, Tafuri WL, Cunha FQ, Teixeira MM. Anti-inflammatory and analgesic effects of the phosphodiesterase 4 inhibitor rolipram in a rat model of arthritis. *Eur J Pharmacol*. 2000;399(2-3):243-249. doi:10.1016/s0014-2999(00)00330-7

40. Bielekova B, Richert N, Howard T, et al. Treatment with the phosphodiesterase type-4 inhibitor rolipram fails to inhibit blood--brain barrier disruption in multiple sclerosis. *Mult Scler*. 2009;15(10):1206-1214. doi:10.1177/1352458509345903

41. Rolan P, Hutchinson M, Johnson K. Ibudilast: a review of its pharmacology, efficacy and safety in respiratory and neurological disease. *Expert Opin Pharmacother*. 2009;10(17):2897-2904. doi:10.1517/14656560903426189

42. Brooks B, Bravver E, Sanjak M, et al. A Single-Center, Randomized, Double-Blind, Placebo-Controlled, Six-Month Clinical Trial Followed by an Open-Label Extension to Evaluate the Safety, Tolerability and Clinical Endpoint Responsiveness of the Phosphodiesterase Type 4 (PDE4) Inhibitor Ibudilast (MN-166) in Subjects with Amyotrophic Lateral Sclerosis (ALS) - STEP-IBUDILAST-ALS-DB-OLE-1 (I8-1B). *Neurology*. 2015;84(14 Supplement). Accessed December 2, 2021. https://n.neurology.org/content/84/14_Supplement/I8-1B

43. Fox RJ, Coffey CS, Cudkowicz ME, et al. Design, rationale, and baseline characteristics of the randomized double-blind phase II clinical trial of ibudilast in progressive multiple sclerosis. *Contemp Clin Trials*. 2016;50:166-177. doi:10.1016/j.cct.2016.08.009

44. Brooks B, Bravver EK, Sanjak M, et al. Ibudilast - Phosphodiesterase Type 4 Inhibitor - Bi-Modal Therapy with Riluzole in Early [Not Requiring Non-Invasive Ventilation (NIV)] Cohort (EC) and Advanced [Requiring NIV] (ANC) Amyotrophic Lateral Sclerosis (ALS) Patients - Single-Center Adaptive Design Six-Month Double-Blind (DB) - Placebo-Controlled Phase 1b/2a Epoch Followed by Six-Month Open Label Extension (OLE) Epoch, Washout (WO) and Post-Washout Epoch (PWO) – Final Report and Future Directions (P6.465). *Neurology*. 2018;90(15 Supplement). Accessed December 2, 2021. https://n.neurology.org/content/90/15_Supplement/P6.465

45. Brooks B, Bravver E, Sanjak M, et al. Adaptive Design Single Center Phosphodiesterase Type 4 (PDE4) Inhibitor - Ibudilast (MN-166-ALS-1201) Phase 1b/2a Clinical Trial Double-Blind (DB) with Open Label Extensions (OLE) [NCT02238626] for Amyotrophic Lateral Sclerosis (ALS) Patients [1] Not Requiring Non-Invasive Ventilation (no-NIV) up to 5 Years (Early Cohort-EC) and [2] Requiring Non-Invasive Ventilation (NIV) up to 10 Years(Advanced NIV Cohort-ANC) from Disease Onset - Behavior of Creatinine as a Biomarker in Short Clinical Trials (P5.003). *Neurology*. 2016;86(16 Supplement). Accessed December 2, 2021. https://n.neurology.org/content/86/16_Supplement/P5.003

46. Fox RJ, Coffey CS, Conwit R, et al. Phase 2 Trial of Ibudilast in Progressive Multiple Sclerosis. *New England Journal of Medicine*. 2018;379(9):846-855. doi:10.1056/NEJMoa1803583

47. Fehlings MG, Ibrahim A, Tetreault L, et al. A global perspective on the outcomes of surgical decompression in patients with cervical spondylotic myelopathy: results from the

For peer review only

prospective multicenter AOSpine international study on 479 patients. *Spine*. 2015;40(17):1322-1328. doi:10.1097/BRS.0000000000000988

48. Fehlings MG, Wilson JR, Karadimas SK, Arnold PM, Kopjar B. Clinical evaluation of a neuroprotective drug in patients with cervical spondylotic myelopathy undergoing surgical treatment: design and rationale for the CSM-Protect trial. *Spine*. 2013;38(22 Suppl 1):S68-75. doi:10.1097/BRS.0b013e3182a7e9b0

49. Kopjar B, Tetreault L, Kalsi-Ryan S, Fehlings M. Psychometric properties of the modified Japanese Orthopaedic Association scale in patients with cervical spondylotic myelopathy. *Spine (Phila Pa 1976)*. 2015;40(1):E23-28. doi:10.1097/BRS.0000000000000648

50. Fehlings MG, Badhiwala JH, Ahn H, et al. Safety and efficacy of riluzole in patients undergoing decompressive surgery for degenerative cervical myelopathy (CSM-Protect): a multicentre, double-blind, placebo-controlled, randomised, phase 3 trial. *Lancet Neurol*. 2021;20(2):98-106. doi:10.1016/S1474-4422(20)30407-5

51. Moon ES, Karadimas SK, Yu WR, Austin JW, Fehlings MG. Riluzole attenuates neuropathic pain and enhances functional recovery in a rodent model of cervical spondylotic myelopathy. *Neurobiol Dis*. 2014;62:394-406. doi:10.1016/j.nbd.2013.10.020

52. Segerdahl M. Pain outcome variables--a never ending story? *Pain*. 2011;152(5):961-962. doi:10.1016/j.pain.2011.03.008

53. Davies BM, McHugh M, Elgheriani A, et al. Reported Outcome Measures in Degenerative Cervical Myelopathy: A Systematic Review. *PLoS One*. 2016;11(8). doi:10.1371/journal.pone.0157263

54. MacDowall A, Skeppholm M, Robinson Y, Olerud C. Validation of the visual analog scale in the cervical spine. *J Neurosurg Spine*. 2018;28(3):227-235. doi:10.3171/2017.5.SPINE1732

55. Parker SL, Godil SS, Shau DN, Mendenhall SK, McGirt MJ. Assessment of the minimum clinically important difference in pain, disability, and quality of life after anterior cervical discectomy and fusion: clinical article. *J Neurosurg Spine*. 2013;18(2):154-160. doi:10.3171/2012.10.SPINE12312

56. Davies BM, Khan DZ, Mowforth OD, et al. RE-CODE DCM (REsearch Objectives and Common Data Elements for Degenerative Cervical Myelopathy): A Consensus Process to Improve Research Efficiency in DCM, Through Establishment of a Standardized Dataset for Clinical Research and the Definition of the Research Priorities. *Global Spine J*. 2019;9(1 Suppl):65S-76S. doi:10.1177/2192568219832855

57. Brouwer WBF, van Exel NJA, van Gorp B, Redekop WK. The CarerQol instrument: a new instrument to measure care-related quality of life of informal caregivers for use in economic evaluations. *Qual Life Res*. 2006;15(6):1005-1021. doi:10.1007/s11136-005-5994-6

58. Tetreault L, Nouri A, Kopjar B, Côté P, Fehlings MG. The Minimum Clinically Important Difference of the Modified Japanese Orthopaedic Association Scale in Patients

For peer review only

with Degenerative Cervical Myelopathy. *Spine (Phila Pa 1976)*. 2015;40(21):1653-1659. doi:10.1097/BRS.0000000000001127

59. Tetreault L, Wilson JR, Kotter MRN, et al. Predicting the minimum clinically important difference in patients undergoing surgery for the treatment of degenerative cervical myelopathy. *Neurosurg Focus*. 2016;40(6):E14. doi:10.3171/2016.3.FOCUS1665

60. Tetreault L, Lange SF, Chotai S, et al. A Systematic Review of Definitions for Neurological Complications and Disease Progression in Patients Treated Surgically for Degenerative Cervical Myelopathy. *Spine (Phila Pa 1976)*. 2019;44(18):1318-1331. doi:10.1097/BRS.0000000000003066

61. Bretz F, Maurer W, Brannath W, Posch M. A graphical approach to sequentially rejective multiple test procedures. *Statistics in Medicine*. 2009;28(4):586-604. doi:10.1002/sim.3495

62. Benjamini Y, Hochberg Y. Controlling the False Discovery Rate: A Practical and Powerful Approach to Multiple Testing. *Journal of the Royal Statistical Society: Series B (Methodological)*. 1995;57(1):289-300. doi:10.1111/j.2517-6161.1995.tb02031.x

63. Chalmers I, Bracken MB, Djulbegovic B, et al. How to increase value and reduce waste when research priorities are set. *Lancet*. 2014;383(9912):156-165. doi:10.1016/S0140-6736(13)62229-1

64. Ergina PL, Cook JA, Blazeby JM, et al. Challenges in evaluating surgical innovation. *Lancet*. 2009;374(9695):1097-1104. doi:10.1016/S0140-6736(09)61086-2

65. Davies BM, Munro C, Khan DZ, et al. Outcomes of Degenerative Cervical Myelopathy From The Perspective of Persons Living With the Condition: Findings of a Semistructured Interview Process With Partnered Internet Survey. *Global Spine J*. Published online November 18, 2020:2192568220953811. doi:10.1177/2192568220953811

66. Davies BM, Mowforth OD, Khan DZ, et al. The development of lived experience-centered word clouds to support research uncertainty gathering in degenerative cervical myelopathy: results from an engagement process and protocol for their evaluation, via a nested randomized controlled trial. *Trials*. 2021;22(1):415. doi:10.1186/s13063-021-05349-8

67. Mowforth OD, Davies BM, Goh S, O'Neill CP, Kotter MRN. Research Inefficiency in Degenerative Cervical Myelopathy: Findings of a Systematic Review on Research Activity Over the Past 20 Years. *Global Spine J*. 2020;10(4):476-485. doi:10.1177/2192568219847439

68. Grodzinski B, Bestwick H, Bhatti F, et al. Research activity amongst DCM research priorities. *Acta Neurochir (Wien)*. Published online February 24, 2021. doi:10.1007/s00701-021-04767-6

69. Neurology TL. A focus on patient outcomes in cervical myelopathy. *The Lancet Neurology*. 2019;18(7):615. doi:10.1016/S1474-4422(19)30168-1

For peer review only

70. Mowforth OD, Davies BM, Kotter MR. The Use of Smart Technology in an Online Community of Patients With Degenerative Cervical Myelopathy. *JMIR Form Res*. 2019;3(2):e11364. doi:10.2196/11364

71. Davies B, Kotter M. Lessons From Recruitment to an Internet-Based Survey for Degenerative Cervical Myelopathy: Comparison of Free and Fee-Based Methods. *JMIR Res Protoc*. 2018;7(2):e18. doi:10.2196/resprot.6567

72. Waqar M, Wilcock J, Garner J, Davies B, Kotter M. Quantitative analysis of medical students' and physicians' knowledge of degenerative cervical myelopathy. *BMJ Open*. 2020;10(1):e028455. doi:10.1136/bmjopen-2018-028455

73. Kalsi-Ryan S, Rienmueller AC, Riehm L, et al. Quantitative Assessment of Gait Characteristics in Degenerative Cervical Myelopathy: A Prospective Clinical Study. *J Clin Med*. 2020;9(3):E752. doi:10.3390/jcm9030752

74. Bhagavatula ID, Bhat DI, Sasidharan GM, et al. Subclinical respiratory dysfunction in chronic cervical cord compression: a pulmonary function test correlation. *Neurosurg Focus*. 2016;40(6):E3. doi:10.3171/2016.3.FOCUS1647

75. Pope DH, Davies BM, Mowforth OD, Bowden AR, Kotter MRN. Genetics of Degenerative Cervical Myelopathy: A Systematic Review and Meta-Analysis of Candidate Gene Studies. *J Clin Med*. 2020;9(1). doi:10.3390/jcm9010282

76. Badran A, Davies BM, Bailey HM, Kalsi-Ryan S, Kotter MR. Is there a role for postoperative physiotherapy in degenerative cervical myelopathy? A systematic review. *Clin Rehabil*. Published online April 1, 2018:269215518766229. doi:10.1177/0269215518766229

77. Davies BM, McHugh M, Elgheriani A, et al. The reporting of study and population characteristics in degenerative cervical myelopathy: A systematic review. *PLOS ONE*. 2017;12(3):e0172564. doi:10.1371/journal.pone.0172564

78. Bogduk N, Govind J. Cervicogenic headache: an assessment of the evidence on clinical diagnosis, invasive tests, and treatment. *Lancet Neurol*. 2009;8(10):959-968. doi:10.1016/S1474-4422(09)70209-1

79. Sheather-Reid RB, Cohen ML. Psychophysical evidence for a neuropathic component of chronic neck pain. *Pain*. 1998;75(2-3):341-347. doi:10.1016/s0304-3959(98)00013-x

80. Voerman VF, van Egmond J, Crul BJ. Elevated detection thresholds for mechanical stimuli in chronic pain patients: support for a central mechanism. *Arch Phys Med Rehabil*. 2000;81(4):430-435. doi:10.1053/mr.2000.3777

81. Zaproudina N, Ming† Z, Närhi M. Sensory and sympathetic disorders in chronic non-specific neck pain. *Funct Neurol*. 2015;30(3):165-171. doi:10.11138/FNeur/2015.30.3.165

82. Westermann A, Rönnau AK, Krumova E, et al. Pain-associated mild sensory deficits without hyperalgesia in chronic non-neuropathic pain. *Clin J Pain*. 2011;27(9):782-789. doi:10.1097/AJP.0b013e31821d8fce

For peer review only

83. Blyufer A, Lhamo S, Tam C, Tariq I, Thavornwatanayong T, Mahajan SS. Riluzole: A neuroprotective drug with potential as a novel anti-cancer agent (Review). *Int J Oncol*. 2021;59(5):95. doi:10.3892/ijo.2021.5275

84. Lee S, Zhao X, Hatch M, Chun S, Chang E. Central Neuropathic Pain in Spinal Cord Injury. *Crit Rev Phys Rehabil Med*. 2013;25(3-4):159-172. doi:10.1615/CritRevPhysRehabilMed.2013007944

85. Kalsi-Ryan S, Singh A, Massicotte EM, et al. Ancillary outcome measures for assessment of individuals with cervical spondylotic myelopathy. *Spine (Phila Pa 1976)*. 2013;38(22 Suppl 1):S111-122. doi:10.1097/BRS.0b013e3182a7f499

86. Tetreault LA, Côté P, Kopjar B, Arnold P, Fehlings MG, AOSpine North America and International Clinical Trial Research Network. A clinical prediction model to assess surgical outcome in patients with cervical spondylotic myelopathy: internal and external validations using the prospective multicenter AOSpine North American and international datasets of 743 patients. *Spine J*. 2015;15(3):388-397. doi:10.1016/j.spinee.2014.12.145

87. Ahuja CS, Wilson JR, Nori S, et al. Traumatic spinal cord injury. *Nat Rev Dis Primers*. 2017;3(1):1-21. doi:10.1038/nrdp.2017.18

88. Fehlings MG, Wilson JR, Kopjar B, et al. Efficacy and safety of surgical decompression in patients with cervical spondylotic myelopathy: results of the AOSpine North America prospective multi-center study. *J Bone Joint Surg Am*. 2013;95(18):1651-1658. doi:10.2106/JBJS.L.00589

89. Martin AR, Leener BD, Cohen-Adad J, et al. Can microstructural MRI detect subclinical tissue injury in subjects with asymptomatic cervical spinal cord compression? A prospective cohort study. *BMJ Open*. 2018;8(4):e019809. doi:10.1136/bmjopen-2017-019809

90. Toyoda H, Nakamura H, Konishi S, Terai H, Takaoka K. Does chronic cervical myelopathy affect respiratory function? *J Neurosurg Spine*. 2004;1(2):175-178. doi:10.3171/spi.2004.1.2.0175

91. Kalsi-Ryan S, Beaton D, Ahn H, et al. Responsiveness, Sensitivity, and Minimally Detectable Difference of the Graded and Redefined Assessment of Strength, Sensibility, and Prehension, Version 1.0. *J Neurotrauma*. 2016;33(3):307-314. doi:10.1089/neu.2015.4217

92. McDermott A, Bolger C, Keating L, McEvoy L, Meldrum D. Reliability of three-dimensional gait analysis in cervical spondylotic myelopathy. *Gait Posture*. 2010;32(4):552-558. doi:10.1016/j.gaitpost.2010.07.019

93. Nagai T, Takahashi Y, Endo K, Ikegami R, Ueno R, Yamamoto K. Analysis of spastic gait in cervical myelopathy: Linking compression ratio to spatiotemporal and pedobarographic parameters. *Gait Posture*. 2018;59:152-156. doi:10.1016/j.gaitpost.2017.10.013

For peer review only

Figure Legend.

Figure 1: Trial Flow Chart.
Eligible and consenting participants will be randomised to an intervention or control arm and followed up for 12 months after surgery.

For peer review only

Figure 1. Trial Flow Chart.

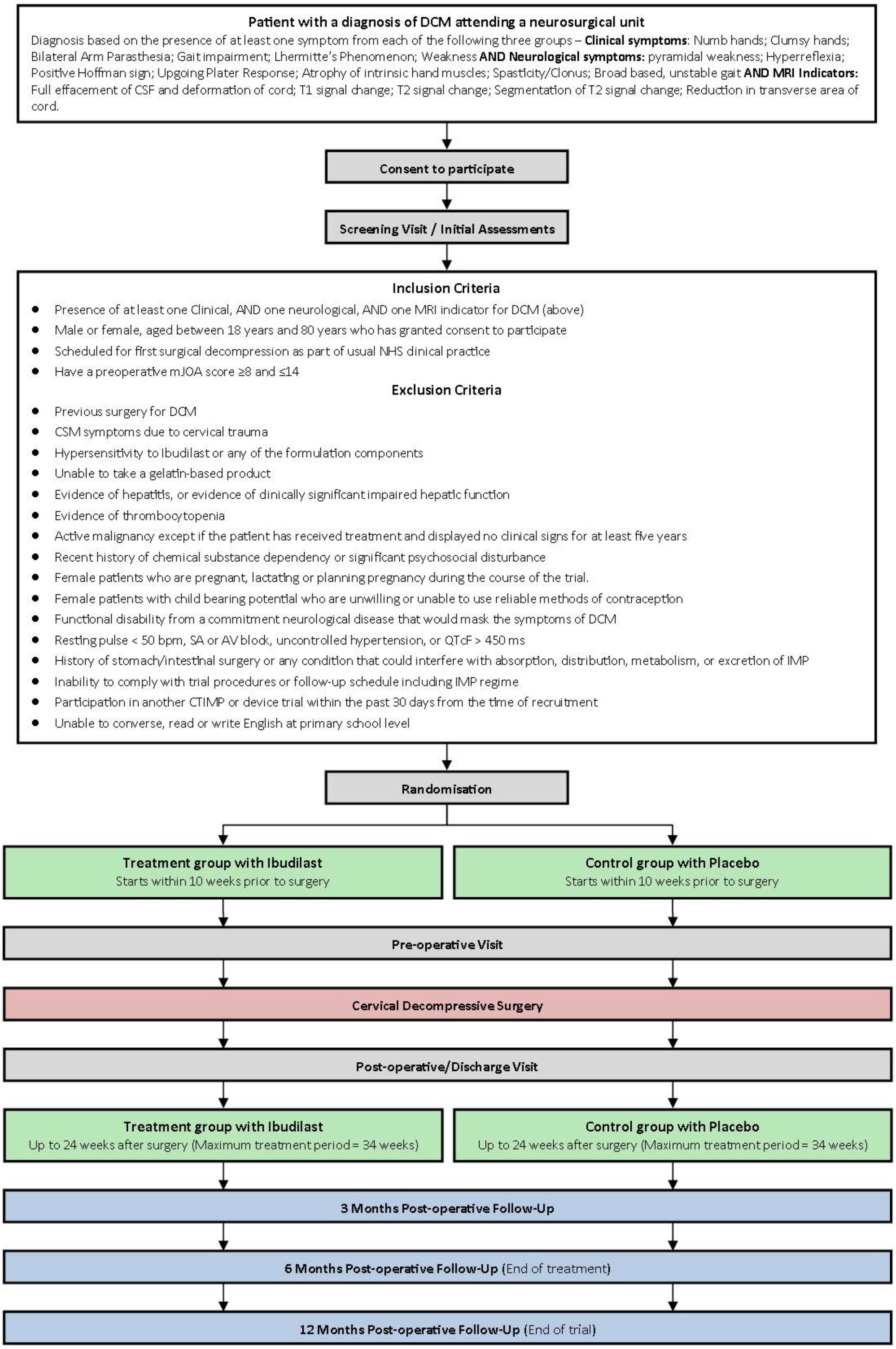

CONSORT 2010 checklist of information to include when reporting a randomised trial*

Section/Topic	Item No	Checklist item	Reported on page No
**Title and abstract**			
	1a	Identification as a randomised trial in the title	Page 1
	1b	Structured summary of trial design, methods, results, and conclusions (for specific guidance see CONSORT for abstracts)	Page 3
**Introduction**			
Background and objectives	2a	Scientific background and explanation of rationale	Page 4-7
	2b	Specific objectives or hypotheses	Page 7
**Methods**			
Trial design	3a	Description of trial design (such as parallel, factorial) including allocation ratio	Page 8
	3b	Important changes to methods after trial commencement (such as eligibility criteria), with reasons	Page 8
Participants	4a	Eligibility criteria for participants	Page 8
	4b	Settings and locations where the data were collected	Page 9
Interventions	5	The interventions for each group with sufficient details to allow replication, including how and when they were actually administered	Page 9
Outcomes	6a	Completely defined pre-specified primary and secondary outcome measures, including how and when they were assessed	Page 10
	6b	Any changes to trial outcomes after the trial commenced, with reasons	n/a
Sample size	7a	How sample size was determined	Page 11
	7b	When applicable, explanation of any interim analyses and stopping guidelines	n/a
Randomisation:			
Sequence generation	8a	Method used to generate the random allocation sequence	Page 13
	8b	Type of randomisation; details of any restriction (such as blocking and block size)	Page 8
Allocation concealment mechanism	9	Mechanism used to implement the random allocation sequence (such as sequentially numbered containers), describing any steps taken to conceal the sequence until interventions were assigned	Page 8/9
Implementation	10	Who generated the random allocation sequence, who enrolled participants, and who assigned participants to interventions	n/a
Blinding	11a	If done, who was blinded after assignment to interventions (for example, participants, care providers, those	n/a

For peer review only - http://bmjopen.bmj.com/site/about/guidelines.xhtml
For peer review only

		assessing outcomes) and how	
	11b	If relevant, description of the similarity of interventions	
Statistical methods	12a	Statistical methods used to compare groups for primary and secondary outcomes	Page 13
	12b	Methods for additional analyses, such as subgroup analyses and adjusted analyses	Page 13-14
**Results**			
Participant flow (a diagram is strongly recommended)	13a	For each group, the numbers of participants who were randomly assigned, received intended treatment, and were analysed for the primary outcome	Page 12
	13b	For each group, losses and exclusions after randomisation, together with reasons	n/a
Recruitment	14a	Dates defining the periods of recruitment and follow-up	n/a
	14b	Why the trial ended or was stopped	n/a
Baseline data	15	A table showing baseline demographic and clinical characteristics for each group	n/a
Numbers analysed	16	For each group, number of participants (denominator) included in each analysis and whether the analysis was by original assigned groups	n/a
Outcomes and estimation	17a	For each primary and secondary outcome, results for each group, and the estimated effect size and its precision (such as 95% confidence interval)	n/a
	17b	For binary outcomes, presentation of both absolute and relative effect sizes is recommended	n/a
Ancillary analyses	18	Results of any other analyses performed, including subgroup analyses and adjusted analyses, distinguishing pre-specified from exploratory	n/a
Harms	19	All important harms or unintended effects in each group (for specific guidance see CONSORT for harms)	n/a
**Discussion**			
Limitations	20	Trial limitations, addressing sources of potential bias, imprecision, and, if relevant, multiplicity of analyses	n/a
Generalisability	21	Generalisability (external validity, applicability) of the trial findings	n/a
Interpretation	22	Interpretation consistent with results, balancing benefits and harms, and considering other relevant evidence	Page 15
**Other information**			
Registration	23	Registration number and name of trial registry	Page 1-2
Protocol	24	Where the full trial protocol can be accessed, if available	n/a
Funding	25	Sources of funding and other support (such as supply of drugs), role of funders	Page 1-2

*We strongly recommend reading this statement in conjunction with the CONSORT 2010 Explanation and Elaboration for important clarifications on all the items. If relevant, we also recommend reading CONSORT extensions for cluster randomised trials, non-inferiority and equivalence trials, non-pharmacological treatments, herbal interventions, and pragmatic trials. Additional extensions are forthcoming: for those and for up to date references relevant to this checklist, see www.consort-statement.org.

For peer review only

Targeting patient recovery priorities in degenerative cervical myelopathy: design and rationale for the RECEDE-Myelopathy trial - Study Protocol

Journal:	*BMJ Open*
Manuscript ID	bmjopen-2022-061294.R1
Article Type:	Protocol
Date Submitted by the Author:	16-Aug-2022
Complete List of Authors:	Davies, Benjamin; University of Cambridge, Department of Clinical Neurosurgery; University of Cambridge<br>Mowforth, Oliver; University of Cambridge, Department of Academic Neurosurgery<br>Yordanov, Stefan; Addenbrooke's Hospital, Neurosurgery<br>Alvarez-Berdugo, Daniel; Cambridge University Hospitals NHS Foundation Trust<br>Bond, Simon; Cambridge University Hospitals NHS Foundation Trust, Cambridge clinical trials unit<br>Nodale, Marianna; Cambridge University Hospitals NHS Foundation Trust<br>Kareclas, Paula; Cambridge University Hospitals NHS Foundation Trust, Cambridge Clinical Trials Unit<br>Whitehead, Lynne; Cambridge University Hospitals NHS Foundation Trust<br>Bishop, Jon; NIHR Surgical Reconstruction and Microbiology Research Centre, Medical Statistician<br>Chandran, Siddharthan; University of Cambridge, Clinical Neurosciences<br>Lamb, Sarah; University of Oxford<br>Bacon, Mark; International Spinal Research Trust<br>Papadopoulos, Marios; St George's Hospital, Department of Neurosurgery London, United Kingdom, Department of Neurosurgery<br>Starkey, Michelle; Myelopathy.org<br>Sadler, Iwan; Myelopathy.org<br>Smith, Lara; Myelopathy.org<br>Kalsi-Ryan, Sukhvinder; Toronto Rehabilitation Institute<br>Carpenter, Adrian; University of Cambridge, Department of Clinical Neurosciences<br>Trivedi, Rikin A.; Cambridge Univ Hosp Fdn Trust<br>Wilby, Martin; The Walton Centre NHS Foundation Trust, Neurosurgery<br>Choi, David; National Hospital for Neurology and Neurosurgery, Department of Neurosurgery<br>Wilkinson, Ian; Division of Experimental Medicine and Immunotherapeutics, Department of Medicine, University of Cambridge; Cambridge Clinical Trials Unit,<br>Fehlings, Michael G.; Toronto Western Hospital<br>Hutchinson, Peter; University of Cambridge, Academic Neurosurgery<br>Kotter, Mark R.N.; University of Cambridge, Department of Clinical Neurosurgery; University of Cambridge
Primary Subject	Neurology

Heading:	
Secondary Subject Heading:	Surgery
Keywords:	NEUROSURGERY, Spine < ORTHOPAEDIC & TRAUMA SURGERY, Neurosurgery < SURGERY

Targeting patient recovery priorities in degenerative cervical myelopathy: design and rationale for the RECEDE-Myelopathy trial – Study Protocol

Benjamin M. Davies[1], Oliver D. Mowforth[1], Stefan Yordanov[1], Daniel Alvarez-Berdugo[2], Simon Bond[2], Marianna Nodale[2], Paula Kareclas[2], Lynne Whitehead[3], Jonathan Bishop[4], Siddharthan Chandran[5], Sarah E Lamb[6], Mark Bacon[7], Marios C. Papadopoulos[8], Michelle Starkey[9], Iwan Sadler[9], Lara Smith[9], Sukhivinder Kalsi-Ryan[10], Adrian Carpenter[11], Rikin Trivedi[1], Martin Wilby[12], David Choi[13], Ian Wilkinson[2,14], Michael G Fehlings[15], Peter Hutchinson[1], Mark R.N. Kotter[1,16]

1) Academic Neurosurgery Unit, Department of Clinical Neurosurgery, University of Cambridge, Cambridge, UK
2) Cambridge Clinical Trials Unit, Cambridge University Hospitals NHS Foundation Trust, Cambridge, UK
3) Pharmacy Department, Cambridge University Hospitals NHS Foundation Trust, Cambridge, UK
4) Birmingham Clinical Trials Unit, Institute of Applied Health Research, University of Birmingham, Birmingham, UK
5) Centre for Clinical Brain Sciences, University of Edinburgh, Edinburgh, UK
6) College of Medicine and Health, University of Exeter, UK
7) International Spinal Research Trust, London, UK
8) Academic Neurosurgery Unit, St. George's University of London, London, UK
9) Myelopathy.org, Cambridge, UK
10) Department of Physical Therapy, Krembil Neuroscience Spine Program, Toronto Western Hospital UHN, Toronto, Canada
11) Wolfson Brain Imaging Centre, Department of Clinical Neurosciences, University of Cambridge, Cambridge, UK
12) Department of Neurosurgery, The Walton Centre for Neurology and Neurosurgery NHS Trust, Liverpool, UK
13) Department of Neurosurgery, National Hospital for Neurology and Neurosurgery, University College London Hospitals NHS Foundation, London, UK
14) Division of Experimental Medicine and Immunotherapeutics, University of Cambridge, Cambridge, UK
15) Division of Neurosurgery, Department of Surgery, Toronto Western Hospital, Toronto, Canada
16) WT MRC Cambridge Stem Cell Institute, Anne McLaren Laboratory, University of Cambridge, UK

Key words: cervical; myelopathy; spondylosis; spondylotic; stenosis; disc herniation; ossification posterior longitudinal ligament; degeneration; disability; recovery; questionnaire

Word Count:

Acknowledgements: Research in the senior author's laboratory is supported by the Cambridge NIHR Brain Injury MedTech Cooperative. MRNK is supported by a NIHR Clinician Scientist Award.

For peer review only - http://bmjopen.bmj.com/site/about/guidelines.xhtml

Disclaimer: This report is independent research arising from a Clinician Scientist Award, CS-2015-15-023, supported by the National Institute for Health Research. The views expressed in this publication are those of the authors and not necessarily those of the NHS, the National Institute for Health Research or the Department of Health and Social Care.

Correspondence:
Dr. Mark R.N. Kotter
Department of Clinical Neurosciences
University of Cambridge
Mrk25@medschl.cam.ac.uk

For peer review only

Abstract

Study Design:

Clinical trial protocol v2.2 Oct 2020

Introduction:

Degenerative cervical myelopathy (DCM) is a common and disabling condition of symptomatic cervical spinal cord compression secondary to degenerative changes in spinal structures leading to a mechanical stress injury of the spinal cord. RECEDE-Myelopathy aims to test the disease-modulating activity of the PDE3/4 inhibitor Ibudilast as an adjuvant to surgical decompression in DCM.

Methods and Analysis:

RECEDE-Myelopathy is a multi-centre, double-blind, randomised, placebo-controlled trial. Participants will be randomized to receive either 60-100mg Ibudilast or placebo starting within 10 weeks prior to surgery and continuing for 24 weeks after surgery for a maximum of 34 weeks. Adults with DCM, who have a modified Japanese Orthopaedic Association score (mJOA) 8-14 inclusive and are scheduled for their first decompressive surgery are eligible for inclusion. The co-primary endpoints are pain measured on a visual analogue scale and physical function measured by the mJOA score at 6 months after surgery. Clinical assessments will be undertaken pre-operatively, post-operatively and 3, 6 and 12 months after surgery. We hypothesize that adjuvant therapy with Ibudilast leads to a meaningful and additional improvement in either pain or function, as compared to standard routine care.

Ethics and Dissemination:

Ethical approval has been obtained from HRA – Wales .The results will be presented at an international and national scientific conferences and in a peer-reviewed journals.

ISRCTN Number: ISRCTN16682024

Strengths and Limitations:

- **Significant patient and public involvement in trial design and outcomes planning.**

For peer review only

- **A pragmatic approach to patient inclusion criteria was utilised – all patient with mJOA between 8-14 and MRI findings of DCM who are scheduled for their first surgery for DCM regardless of approach are able to be included.**
- **We will explore and compare both clinical and objective findings and validated questionnaire and multiple patient reported outcomes.**
- **A limitation is the need of close patient follow-up and rigorous screening with additional blood tests to comply with drug monitoring and assessments needed.**

For peer review only

Introduction

Here we present the study rationale and design of RECEDE Myelopathy (**Re**generation in **Ce**rvical **De**generative Myelopathy), the first regenerative medicine trial for degenerative cervical myelopathy (DCM), which aims to test disease-modulating activity of the PDE3/4 inhibitor Ibudilast as an adjuvant to surgical decompression.

DCM is a common and progressive condition with devastating impact on quality of life

DCM is the most common cause of spinal cord impairment world-wide,[1,2] with some estimates of the prevalence as high as 2% of adults.[2,3,4] It arises when arthritic or developmental changes in the cervical spine compress the spinal cord, causing a progressive slow-motion spinal cord injury.[5] As a degenerative pathology the incidence is expected to rise in an ageing population.[6,7]

The consequences of DCM are numerous, varied, and often progressive. Symptoms include pain, loss of dexterity, imbalance and frequent falls, incontinence and in extreme circumstances paralysis.[1,8,9,10,11] A recent comparative study found sufferers have amongst the worst quality of life scores of all chronic disease,[12,13] and this is likely to also negatively impact on their supporters.[14] The cost of DCM to society has not been measured yet, but it is likely to be significant. Consequently, improving recovery after surgery is a significant unmet need and there is strong evidence that surgical treatment for DCM is cost-effective.[15]

Surgery is the only evidence-based treatment for DCM

At present, the only effective treatment for DCM is surgery. Whilst surgery can stop disease progression, the existing damage does not fully recover[16] and people with DCM retain life-

long disabilities with severe impact on quality of life.[12,13] Many remain unable to return to full time work and reliant on others for day-to-day activities.[17] Given the severe long-term consequences of DCM, treatment alternatives that promote recovery are desperately needed.

Phosphodiesterase 3 inhibition promotes functional recovery in preclinical DCM

The Mitogen-Activated Protein Kinases (MAPK) play a vital role in intracellular signalling.[18] In response to extracellular stimuli, such as neurotransmitters, inflammatory factors or stress conditions, this family of interconnected serine/threonine kinases coordinates a diverse range of intracellular processes, including cell differentiation, proliferation and apoptosis, inflammation and stress responses.[19] This signalling pathway and its modulation have therefore been linked to many diseases including cancer, asthma, stroke, multiple sclerosis and Alzheimer's dementia. More recently, preclinical studies, including our own, have demonstrated that its modulation via inhibition of a class of enzymes called phosphodiesterases (PDE), can improve functional recovery and reduce the perception of pain following damage to the central nervous system.[20,21,22]

PDEs hydrolyse the intracellular messenger cyclic AMP (cAMP).[20,23] This results in modulation of MAPK signalling.[24,25] Inhibition of PDE3 is particularly attractive in DCM as treatment with the selective PDE3 inhibitor cilostozol resulted in improved functional recovery in a rat model of DCM,[26] likely by improving latent ischemia.

Improvements following surgery are associated with axon sprouting, re-myelination, and immunomodulation

In DCM, tethering and compression of the spinal cord initiates a cascade of secondary injury events, including ischemia, inflammation and apoptosis that ultimately cause increased neurological deficits.[5,27,28] The partial reversal of symptoms after surgery highlights an inherent, albeit attenuated, regenerative capacity of the spinal cord.[16,29] This is echoed by post-mortem studies and our preclinical data, which indicate that neurological recovery following decompression is associated with axonal plasticity, re-myelination, and modulation of the immune response.[29,30,31] Enhancing axonal plasticity and re-myelination is therefore key to improving outcomes after DCM.[32]

Phosphodiesterase 4 inhibition can promote functional recovery and modulate pain in preclinical models

For peer review only - http://bmjopen.bmj.com/site/about/guidelines.xhtml

PDE4 is another isoform of phosphodiesterase inhibitors, which has demonstrated preclinical benefits on axon outgrowth[20] and remyelination.[22] The best characterised application of PDE4 inhibitors involves preclinical models of traumatic spinal cord injury using a drug called rolipram.[20,21] Unanimously, these have demonstrated that modulation of the PDE4 cascade is able to benefit recovery. In addition, our own work demonstrated that inhibition of PDE4 is able to stimulate the regenerative response of a CNS stem cell population termed oligodendrocyte progenitor cells and engage in re-myelination,[22] a process that has been observed in post-mortem spinal cords affected by DCM.[30]

PDE4 inhibition also has a role in modulating the perception of pain. Central to the development and maintenance of chronic pain syndromes is glial activation within the central nervous system, which enhances pain sensitivity via neuronal-glial interactions.[33] Modulation of MAPK via PDE4 inhibition has demonstrated a reduction in pain in several preclinical models.[34,35,36,37] Bao *et al*. (2011) found that PDE4 inhibition improved not just motor recovery but also resulted in a reduction in neuropathic pain in a rat model of spinal cord injury.[38] PDE4 inhibition also has an anti-inflammatory effect, increasing cAMP production in leukocytes and therefore reducing the release of tumour necrosis factor-alpha, a potent inflammatory mediator and peripheral pain stimulus.[39]

Ibudilast is a potent PDE4 inhibitor with an excellent human safety profile

The majority of preclinical studies described have used rolipram for PDE4 inhibition. Whilst rolipram is a potent and selective PDE4 inhibitor, experience from translational trials, most recently in multiple sclerosis (MS),[40] have demonstrated poor tolerability in humans due to significant nausea and vomiting. The MS trial had to be terminated due to a lack of efficacy and poor tolerability. Additionally, preclinical evidence has demonstrated a narrow therapeutic window, with potentially adverse neurological sequalae if missed.

An alternative is Ibudilast (MN-166).[23] Ibudilast is a potent PDE4 inhibitor, with additional PDE3 and 5 receptor activity. Modulation of PDE3 is also attractive in DCM as it led to improved function in a preclinical model of DCM.[26] Another attractive feature of Ibudilast is that it has been in clinical use for over 20 years for the treatment of asthma and post-stroke dizziness, without tolerability issues.[41]

Ibudilast is currently under investigation for a number of other neurological conditions,

For peer review only - http://bmjopen.bmj.com/site/about/guidelines.xhtml

including alcohol [NCT03489850] and methamphetamine [NCT01860807] addiction, glioblastoma [03782415], amyotrophic lateral sclerosis (ALS)[42] and multiple sclerosis[43] in a series of double blind, placebo randomised controlled trials.

For ALS, a single Phase I/II trial has been completed. Two ALS cohorts of early stage disease and advanced stage disease requiring ventilation, were randomised 1:1 to receive Ibudilast or placebo. Overall, the primary endpoint of safety and tolerability was met. In the early stage disease takers, Ibudilast was associated with a significant increase in survival, and delayed requirement for ventilation.[44] Treatment effects were linked to per-protocol adherence to therapy.[45] A Phase III trial is now planned.

For MS, two phase II trials have been completed. The first one evaluated relapsing remitting MS; whilst it did not prevent the development of new brain lesions, it slowed the progression of brain atrophy in a dose dependent fashion. The second one, a follow-up study in progressive MS, found that Ibudilast significantly slowed the progression of brain atrophy.[46]

Of note, typical daily dosing in these trials ranged from 60-100mg, which is greater than the currently licensed dosing of 10-20mg per day for routine clinical practice. Whilst trials confirmed overall tolerability and safety for use of Ibudilast in these doses in humans, findings do indicate a dose dependent relationship for gastro-intestinal side effects, such as nausea, and headaches and, in a minority of cases, this led to discontinuation of therapy by participants.

RECEDE-Myelopathy (Regeneration in Cervical Degenerative Myelopathy)

RECEDE-Myelopathy is a multi-centre, double-blind, randomised, placebo-controlled trial assessing the efficacy of Ibudilast as an adjuvant treatment to decompressive surgery for degenerative cervical myelopathy. The specific mechanism of action of Ibudilast is highly suited to address both functional outcome and neuropathic pain in DCM. Therefore, prompted by the direct involvement of people with DCM in designing the study, RECEDE Myelopathy has an infrequently used study design of two co-primary endpoints. It is designed and powered to detect response of patients to Ibudilast with regards to function or pain, independently, as well as a response to both endpoints. We hypothesise that Ibudilast promotes functional outcome and reduces pain in surgically treated DCM.

For peer review only - http://bmjopen.bmj.com/site/about/guidelines.xhtml

For peer review only

Methods

Study design and objectives

RECEDE-Myelopathy (**Re**generation in **Ce**rvical **De**generative **Myelopathy)** is a multi-centre, double-blind, randomised, placebo-controlled trial assessing the efficacy of Ibudilast as an adjuvant treatment to decompressive surgery for degenerative cervical myelopathy. Participants will be randomized to receive either 60-100mg Ibudilast (interventional arm) or placebo (control arm) starting within 10 weeks prior to surgery and continuing for 24 weeks after surgery for a maximum of 34 weeks of treatment. Pre-operative treatment may leverage the effects of inhibition of PDE3, whilst post-operative treatment aims at regeneration-inducing effects outlined above. The primary objective will be to compare improvement in pain or physical function at 6 months after surgery between the two arms of the trial. We hypothesize that adjuvant therapy with Ibudilast leads to a meaningful and additional improvement in either pain or function, as compared to standard routine care (decompressive surgery). Planned start date for study recruitment is September 2021, with planned end being September 2025.

Patient and Public involvement (PPI) - aligning research with patient priorities

The involvement of public and patients representatives in research is recognised to be of key importance to ensure it delivers meaningful, practice-changing information. [47,48, 49, 50] As with many fields, this has been a problem for DCM. [51, 52, 53] To address this issue, we founded Myelopathy.org, the first and so far only charity for people with DCM. Whilst in its infancy, the platform has become an international focus for people with DCM, hosting a peer-to-peer support community (Myelopathy Support) of over 2000 users.[54] This has enabled larger scale insights into the perspective of individuals with DCM[17, 55, 56,] and ultimately led to RECODE-

DCM, a James Lind Alliance-led initiative to identify and define the research priorities for DCM.[51] (https://aospine.aofoundation.org/research/recode-dcm)

Definition of recovery priorities for people with DCM

AO Spine Research Objectives and Common Data Elements for Degenerative Cervical Myelopathy (AO Spine RECODE-DCM) is an international initiative to create a 'Research Toolkit' to help improve and accelerate knowledge gained in DCM and help to improve outcomes. As part of RECODE DCM, a focus group of people with DCM was created with the objective to develop recovery domains. These were subsequently prioritised via an international, online survey (n = 485).[10] In contrast to the research focus to date, [52,, 57] pain emerged as the number one recovery priority, closely followed by hand, and walking function. Consequently, the development of adjuvant treatments for DCM should be most usefully focused on reducing pain and improving limb function.

Patient screening and eligibility

A summary of the study flow diagram, including full inclusion and exclusion criteria, is presented in Figure 1. In summary, adults (age 18-80) with a diagnosis of DCM (participants must have at least one MRI indicator, clinical symptom, and neurological sign from Table 1 to be eligible for inclusion) and a disease severity of modified Japanese Orthopaedic Association scale (mJOA) 8-14 inclusive, scheduled for their first decompressive surgery, will be approached to consider participation in RECEDE-Myelopathy.

Table 1: Trial criteria for diagnosis of DCM. Participants must have at least one MRI indicator, clinical Symptom, and neurological sign to be eligible for inclusion.

MRI Indicators	Clinical Symptoms	Neurological Signs
Effacement of CSF and deformation of cord	Numb hands	Pyramidal weakness
T1 signal change	Clumsy hands	Hyperreflexia
T2 Signal change	Bilateral arm paraesthesia	Positive Hoffman sign
Segmentation of T2 signal change	Gait impairment	Upgoing plantar response
Reduction in transverse area of cord	Lhermitte's phenomenon	Atrophy of intrinsic hand muscles
	Weakness	Spasticity/clonus
		Broad based, unstable gait

For peer review only - http://bmjopen.bmj.com/site/about/guidelines.xhtml

Eligibility will be further assessed against exclusion criteria, largely dictated by safety requirements for use of Ibudilast, and in precluding masking of treatment effects. This includes, concomitant lumbar canal stenosis or other neurological condition, presentation with symptoms due to trauma (e.g., central cord syndrome), a history of allergy to Ibudilast, any of its formulation or that of the placebo, pregnancy, unwillingness to use reliable contraception, active malignancy, liver impairment or thrombocytopaenia. The latter will be assessed via serum biochemistry and haematological assessment. A full list of exclusion criteria can be find in table 2.

For peer review only

Table 2. Exclusion Criteria

1	Previous surgery for degenerative cervical myelopathy
2	Degenerative cervical myelopathy symptoms due to cervical trauma, determined at the discretion of the investigator
3	Hypersensitivity to Ibudilast or any of the formulation components
4	Evidence of acute hepatitis, clinically significant chronic hepatitis, or evidence of clinically significant impaired hepatic function through clinical and laboratory evaluation (including ALP >1.5x ULN; ALT or AST >2x ULN; GGT >3x ULN)
5	Evidence of thrombocytopenia at screening through laboratory evaluation including platelet count <5000
6	Active malignancy defined as a history of invasive malignancy, except if the patient has received treatment and displayed no clinical signs and symptoms for ≥5 years
7	Recent history (≤3 years) of chemical substance dependency or significant psychosocial disturbance that may impact the outcome or trial participation
8	Female patients with childbearing potential who are unwilling or unable to use reliable methods of contraception
9	Female patients who are pregnant, lactating or planning pregnancy during the course of the trial
10	Inability to comply with trial procedures or follow-up schedule including IMP regime
11	Unable to take gelatin-based product
12	Participation in another CTIMP or device trial ≤30 days before the time of recruitment
13	Functional disability from a concomitant neurological disease that would mask the symptoms of degenerative cervical myelopathy, determined at the discretion of the investigator. Including but not limited to stroke with a residual disability, cerebellar ataxia, Parkinson's disease, symptomatic lumbar stenosis, and multiple sclerosis.
14	Resting pulse < 50 bpm, sinoatrial or atrioventricular block, uncontrolled hypertension, or corrected QT interval (QTcF) >450 ms

For peer review only - http://bmjopen.bmj.com/site/about/guidelines.xhtml

15	History of stomach or intestinal surgery or any other condition that could interfere with, or is judged by the investigator to interfere, with absorption, distribution, metabolism, or excretion of IMP
16	Unable to converse, read, or write English

Enrolment and randomisation

Those patients who satisfy the screening criteria and agree to study participation are enrolled and randomized at 1:1 to one of the two treatment arms. A web-based randomisation system (Sealed envelope) performing stratified blocked randomisation will be used stratifying by baseline mJOA (<12 vs. >=12), age (<60 years vs. >=60 years) and time to onset of the disease (>6 months vs. <=6 months); random block size will be used. Throughout randomisation and follow-up, the subjects, physicians, and data collectors remain blinded to group allocation.

Treatment description and dosage modification

The investigational medicinal product (IMP) is a 24-34 week course of Ibudilast or matched placebo in an escalating dosage regimen up to a maximum of 100mg daily if tolerated. The escalating dosage regimen is to minimise gastrointestinal side effects. Ibudilast is available in 10mg capsules, and therefore the IMP will be provided as such. The placebo is identical in shape, size, and color to the Ibudilast capsule, and participants will be provided with the same instructions.

Participants will start treatment within 10 weeks prior to surgical decompression and will continue taking drug for up to 24 weeks post-surgery. The excretion half-life of Ibudilast is approximately 20 hours. The IMP will be taken in divided doses, twice daily, morning and evening, for a maximum of 34 weeks. Because this is the first surgical trial with Ibudilast, and to mitigate any potential interference on the coagulation system, treatment will be halted 5 days prior to surgery and resumed at the previous maximum dose right after operation.

Ibudilast is associated with gastro-intestinal side effects, such as nausea and dyspepsia. Alongside dose escalation, participants will be instructed to take trial medication with food or within an hour of eating to improve gastrointestinal tolerability. In the event of minor gastrointestinal complaints, participants will be offered symptomatic treatment in the first instance, in conjugation with ongoing IMP therapy. If this is unsuccessful, or not agreeable to participants, the trial therapy will be decreased in decrements of 20mg every 5 days, until a

For peer review only

tolerable dosing level is achieved, or the drug is stopped. If a participant cannot tolerate a minimum daily dosage of 60mg despite additional supportive measures, treatment within the trial will be stopped.

Surgery

There are a number of different approaches used to decompress the spinal cord in DCM. No surgical approach has been shown to be superior, and the consensus is that the approach needs to be tailored to the specific anatomy. The surgical care of participants will therefore be at the discretion of the treating clinician and not protocolised.

Outcome measures and follow up

Two patient-informed co-primary endpoints: pain and function

Inhibiting phosphodiesterases 3 and 4 with Ibudilast has the potential to benefit both pain and functional recovery by promoting repair mechanisms in the spinal cord as well as exerting neuroprotective effects. This provides a unique opportunity to address the most important recovery priorities identified by individuals with myelopathy. Therefore, RECEDE-Myelopathy has two outcome targets: pain and physical function.[10] These co-primary endpoints will be assessed at 6 months after surgery, a time point when the majority of recovery will have been achieved. [57]

The study is thus powered to detect meaningful changes with regards to the co-primary endpoints independently from each other, i.e., it is designed to establish whether Ibudilast has beneficial effects on function or pain alone or whether it beneficially modulates both end points

Co-primary endpoint 1. The international standard, and most validated measure for assessment of function in DCM, is the mJOA scale.[16, 58,59] The mJOA is a composite score of upper and lower limb muscular function, upper limb sensory function and bladder function.

Co-primary endpoint 2. Pain has been identified as the recovery priority of DCM patients. The most common form is neck pain,[9] with a neuropathic component that is responsive to neuroprotective treatments. [60, 61]

For peer review only - http://bmjopen.bmj.com/site/about/guidelines.xhtml

Whilst numerous tools have been developed for the measurement of pain,[62] the Initiative on Methods, Measurements and Pain Assessment in Clinical Trials (IMMPACT) agree that pain intensity scales provide the most relevant outcome measure for demonstrating efficacy. In DCM the visual analogue scale (VAS) is the most popular example of this.[63] Although not exclusively validated for DCM, the psychometric properties of VAS neck and VAS arm pain have been evaluated in degenerative disease of the cervical spine,[64,65] with VAS neck pain having better repeatability.

This design will address the most important priorities of people with DCM.[10] It leverages the mechanism of action of Ibudilast to maximise the chances of demonstrating the benefit of the studied intervention. It will increase the knowledge that can be gained through the study and demonstrate whether the proposed mechanisms of neuroprotection and regeneration can be applied to promote function and/or reduce pain. Finally, the dual end-point design will make the study more efficient than conducting two independent trials. The chosen two endpoint design will hence increase the value of the study.

Secondary and exploratory endpoints

Clinical assessments will additionally be undertaken pre-operatively, post-operatively and 3, 6 and 12 months after surgery. The disability reported in the context of DCM is wide ranging. In the absence of a consensus dataset,[51.] an issue that we are currently attending to as part of RECODE-DCM, a variety of clinician administered and patient reported outcome measures will be used to provide a comprehensive assessment. A full list of assessments and their time-points is presented in table 3.

Table 3: Schedule of Assessments

Assessments	Screening visit and initial assessments	Randomisation	Start of IMP (within 2 -3 month prior to Surgery)	Pre-Operative assessments (within 21 days prior to surgery)	Surgery	Post-operatively/ Discharge (within 14 days post-surgery)	3- months Post Operatively (±21 days)	6-months Post Operatively (±21 days)	12-months Post Operatively (±21 days)
Informed consent	X								
Eligibility Assessment	X								
Demographics	X								
Medical history & DCM characteristics	X								

For peer review only - http://bmjopen.bmj.com/site/about/guidelines.xhtml

Concomitant medication	X			X			X	X	X
Blood Tests (FBC, LFT, E/U/C, TFTs)	X			X			X	X	X
ECG	X								
Urine analysis	X								
Pregnancy test	X								
Randomisation		X							
Neurological examination	X			X		X	X	X	X
mJOA	X			X			X	X	X
30m Walk test	X			X			X	X	X
GRASSP-Cervical Myelopathy	O			O			O	O	O
SCIMv3	O			O				O	
WHO performance status				X					
Neck Disability Index	O			O		O	O	O	O
VAS Pain	X			X		X	X	X	X
SF-36	X			X			X	X	X
EQ5D / Health Resource Usage	X			X			X	X	X
Quick-DASH	O			O			O	O	O
Carer QoL (sub-study)	X			X			X	X	X
Review of AEs			X	X		X	X	X	X
Dosing Diary	X								
Dispensing of IMP			X			X	X		
Serum sample for PK studies	X			X	X		X	X	X
Compliance Assessment				X		X	X	X	
IMP review				X		X	X	X	
Respiratory Physiology & muscle function				X				X	
MRI				X				X	
Gait Lab (sub-study)				X			O	X	
Surgery details					X				
Surgery complications						X	X	X	X
Hospital discharge						X			
CSF sample					O				

Not all assessments will be conducted at every time point, or be mandated, to reduce participant and investigator burden. Assessment is also extended to carers of participants. Building on our preliminary finding of reduced quality of life amongst DCM carers,[14] the Care Quality of Life instrument (CarerQol) will be used to evaluate this.[65.]

Adaptive sample size design

The minimum clinically important difference (MCID) for the mJOA is estimated to be between 1 and 2 points.[66] Although not exclusively validated for DCM, the MCID for VAS neck and VAS arm pain has been calculated for degenerative disease of the cervical spine with values ranging from 8-26mm.[64, 65] Both VAS pain and mJOA improve more than the MCID with surgery alone,[57] and the amount of change is linked to the pre-operative baseline.[66] Consequently, in consensus with patients we have determined the MCID of the VAS pain score as being 1cm and for the mJOA 1 point. This has been modelled to ensure statistical power across all baseline scenarios.

On this basis, a total sample size of 362 participants under equal randomisation will provide 85% power to detect a difference of 1 between treatment arms on the mJOA scale (assuming a standard deviation (SD) of 2.89), using a two-sided t-test at a 2.5% significance level to adjust for multiple comparisons.[67] The trial is also powered to detect a similar difference on the VAS neck pain scale (assuming a difference of 1 and a SD of 2.88).

For peer review only - http://bmjopen.bmj.com/site/about/guidelines.xhtml

A blinded interim analysis will be conducted to refine the power calculation. The aim will be to reassess the sample size in time to allow any potential extension and increase in sample size to be put into effect. Reduction in sample size will not be permitted. Any sample size increase will be based on checking the assumption regarding the SD, and will not estimate any treatment effect, hence no subsequent adjustment to future analyses is needed.
Under such a framework, the theoretical optimal time to schedule such an interim analysis would be just as the last patient is recruited under the original sample size (n=362) following which a decision could be taken to either halt or extend recruitment. However, for reasons of practicality a window for the interim analysis will be up to a period of 4 months before reaching the total sample size.

The SD and correlation of both endpoints will be reassessed using data pooled across the arms. The three possible statistically significant conclusions of the formal hypothesis testing (VAS; mJOA; both) will be provided with revised target sample sizes needed to achieve 85% power under the same MCID values, but with revised estimates for the SD values and correlation. A recommended revised sample size will be the smallest of the three new target sample sizes or the original sample size if this is larger; hence the recommended sample size will never be a reduction from the original.

The next step of the interim analysis will be to calculate the conditional power of the three possible positive outcomes based on, the estimated unblinded treatment effects from the current data, plus, the distribution of future data from the revised sample size under the corresponding combinations of true treatment effects (MCID or zero), and SD and correlation estimates from the first step. If all three conditional power values are less than 30% then the recommendation would be to halt the study.

Trial monitoring

All data collected during the trial will be recorded into a Case Report Form (CRF), which will be labelled using a participant's unique trial ID and date of birth. CRFs will be completed by the local research team and copies will be sent to trial coordination centre, where it will be entered into a central digital database. Safety assessments will be conducted by local investigators and reported and handled according to a predefined trial protocol. This includes a mechanism to capture surgical complications.[68] The Trial Steering Committee

For peer review only - http://bmjopen.bmj.com/site/about/guidelines.xhtml

(TSC) will provide overall supervision with respect to the conduct of the trial. The TSC will consist of an independent Chairperson (Prof Michael Fehlings), a PPI representative (Mr Iwan Sadler), independent clinical and science experts (Prof Marios Papadopoulos and Dr Mark Bacon), clinical pharmacology and neurosurgery experts (Prof Ian Wilkinson and Prof Peter Hutchinson), the Chief Investigator and members of the Trial Management Group (e.g., trial statistician, trial manager). The ethical and safety aspects of the trial will be overseen by an independent Data Monitoring Committee (DMC) who will meet once a year and their meetings will be timed so that reports can be fed into the TSC meetings. Safety assessment will be performed for every participant since consent and until end of their participation in the trial. To date, there are no known expected serious adverse reactions (SAR) for Ibudilast, and thus any reported SAR will be considered a suspected unexpected serious adverse reaction (SUSAR). Furthermore, surgical complications will be followed up as events of special interest to be reviewed by the DMC.

Statistical methods

The primary endpoint and key secondary endpoints are all measured on a continuous scale. A comparison of mean values between treatment arms, adjusting for baseline covariates, will be provided using linear regression. Estimates, standard errors, 95% confidence intervals and p-values will be provided.

For formal hypothesis testing, a closed testing approach will be used to deal with multiple endpoints.[69] Initially either of the co-primary endpoints (mJOA or VAS neck pain) may test a null hypothesis of zero mean difference at a 2-sided 2.5% significance level, [70] with the remaining primary endpoint tested at 5% significance level. This will enable us to determine whether the study drug is effective on pain or function independently.

Subsequently a gate-keeping approach will be used where an endpoint below the primary endpoint in the pre-specified ordering is only tested if all the preceding endpoints reject the null hypothesis, using the nominal p-value. If an endpoint does not reject the null, then all endpoints below it have the same conclusion-not rejecting the null-regardless of their nominal p-value. The ordering is, after primary endpoints, SF-36 PCS and then SF-36 MCS.

For peer review only - http://bmjopen.bmj.com/site/about/guidelines.xhtml

Secondary endpoints will be compared between treatment arms using approach regression techniques: linear regression for continuous endpoints, logistic regression for binary endpoints, and Cox regression for time-to-event.

The following baseline covariates, in addition to the baseline value of the endpoint, will be used to adjust all comparisons

- Time to onset
- Smoking status (yes/no)
- Age
- Psychiatric comorbidities (yes/no)
- Impaired gait (yes/no)

A detailed statistical analysis plan will be produced before the final database lock.

Discussion

This is the first regenerative medicine trial for DCM. It is also the first trial to target all the recovery priorities for people with DCM, namely pain and upper and lower limb function as primary endpoints.[10] This is significant, as in the recent evaluation of Riluzole as a perioperative neuroprotective therapy in DCM, whilst the primary end-point (1-point change in mJOA) was not met, VAS neck pain, a secondary end-point, improved significantly. [58] However, as a secondary endpoint the causal link can only be tentative.

RECEDE-Myelopathy addresses 5 of the 10 top priorities identified by RECODE-DCM

Priority 1 - Raising awareness[1,72]:

- RECEDE-Myelopathy is the first regenerative medicine trial for DCM. It is the second powered DCM CTIMP world-wide. We will seek to leverage this fact to attract attention to DCM by optimising communication before, during and after the trial, aiming at maximising our audience, to include patient organisations, a wide range of health care providers and the scientific community. We also aim to break into non-specialist mainstream media.

Priority 2 – Assessment and monitoring:

- RECEDE-Myelopathy will help to standardise assessment and monitoring across study centres, and thus promote the implementation of the recent international

For peer review only - http://bmjopen.bmj.com/site/about/guidelines.xhtml

guidelines.[16] Additionally, a number of new secondary endpoints are included for the first time in a clinical trial of DCM, including gait[73] and respiratory physiology.[74]

Priority 5 – Developing a better understanding of the pathophysiology of DCM[75]

- RECEDE-Myelopathy tests the hypothesis that MAPK signalling mediated by PDE3/4 can promote recovery after DCM. It will serve as a platform for sub-studies, including imaging studies, and molecular biology studies on blood draws and CSF.

Priority 6 – Rehabilitation:

- There are no evidence-based measures to promote rehabilitation in DCM.[76] RECEDE-Myelopathy will investigate a drug that has the possibility of improving functional outcomes in DCM.

Priority 7 – Novel therapies:

- At present, surgery is the only possible treatment for DCM. If successful, RECEDE-Myelopathy will pave the way for the first evidence-based non-surgical adjuvant treatment.

Neuropathic origins of neck pain in DCM

Pain has been identified as the recovery priority of people with DCM.[10] Where assessed, previous trials have focused on neck (or axial) pain and arm pain.[61,58,77] Our findings in a survey of 230 patients, found neck pain was the most commonly reported first symptom of DCM (13%), and with respect to pain, twice as common as limb pain (7%). Moreover, overall neck pain was experienced more often (80%) than arm pain (70%) (56). In addition, individuals can be affected by atypical pain syndromes such as headache.[8,78,11]

Counter to the prevalent belief that neck pain is mainly caused by arthritic changes to the spine, an emerging literature points to a neuropathic origin. First, arthritic changes are omnipresent with progressive age, causing increasing levels of cord compression.[3,9] In many instances this does not lead to neck pain, even in the context of DCM.

A neuropathic component of chronic neck pain has long been postulated. For example, a psychophysical study measuring responses to electro-cutaneous stimulation in subjects with chronic neck pain found evidence of secondary hyperalgesia which, in turn, implies central sensitisation of nociceptive pathways.[79] The results were compatible with studies which identify potential anatomical origins of chronic neck pain but provide evidence that central sensitisation may be the relevant mechanism of pain production.

For peer review only - http://bmjopen.bmj.com/site/about/guidelines.xhtml

A single centre study investigated the relation between pain provoking cervical segments identified by diagnostic dorsal root blockades and elevation of quantitative sensory testing of the cervical dermatomes using Semmes-Weinstein monofilaments in patients suffering from neck pain but not radiculopathy. This revealed a systematic elevation of detection thresholds, an adaptation in contrast with, but not contradictory to, central sensitization of high threshold neurons in chronic pain.[80]

More recently, a study of non-specific neck pain investigating neuropathic components, and in particular neck pain-associated functional abnormalities related to sensory and sympathetic innervation demonstrated signs of functional impairment of innervation. These were reflected in changes in tactile sensitivity and vasoactive sympathetic function and may be based on both central and peripheral mechanisms.[81] Of note, osteoarthritic pain does not change sensory or pain thresholds in individuals with neck pain.[82]

Another striking piece of evidence in support of a neuropathic component underlying neck pain are the findings of the CSM-Protect trial, the first adequately powered double blind randomised controlled drug trial for DCM.[50](58) Riluzole is an approved neuroprotective drug in clinical use for amyotrophic lateral sclerosis. It has been linked to reducing glutamatergic excitotoxicity in neurons via a number of mechanisms.[83] Although Riluzole treatment did not alter functional outcome in DCM, significant improvements in neck pain were detected.[50](58)

A neuropathic pain component in DCM is further supported by recent preclinical findings which echoed the findings of the clinical trial.[51](59) Finally, it must not be overlooked that DCM is a form of spinal cord injury. The importance of neuropathic pain in SCI is well established.[84]

Outcome assessment in DCM is a challenge for translational research and will be further evaluated.

As outlined, the selection of VAS neck pain, and the mJOA is based on the current best available assessments. Whilst the mJOA is a robust, and fully validated measure, this scale does not capture pain and has a reduced sensitivity to change in milder disease.[59] Presently, there is no combined assessment tool of function and pain validated for DCM,[85] with pain

For peer review only - http://bmjopen.bmj.com/site/about/guidelines.xhtml

typically captured using visual analogue scales.[61,,77] RECODE-DCM, a parallel international consensus initiative is underway to determine the most suitable outcome measurements for DCM. [51]

This has led to two important considerations in the design of this trial: the selection of the inclusion criteria and of the trial endpoints.

The eligibility criteria were designed to ensure the most cost-efficient design and likelihood of success.[85] The surgical treatment of mild DCM is controversial,[16] and therefore risks underrepresentation in this trial if included. Additionally, surgical treatment alone is likely to return a maximum mJOA score in mild disease.[86] Alongside the recognised plateau effect of higher mJOA scores, this therefore risks masking a treatment effect. To prevent these effects, only moderate/severe scores in the mJOA are included in the trial. Similarly, this is the concern for neurological comorbidities or previously treated myelopathy. The mJOA is a measure of functional disability and therefore neurological comorbidities may instead be measured.[85] This is why other neurological comorbidities that could mask the symptoms of DCM are excluded from the trial. Based on experience from traumatic spinal cord injury,[87] it is anticipated that the biological recovery capacity is altered in patients with previously treated myelopathy. Additionally, this subgroup has received relatively little research,[77] and the data informing the surgical response and MCID is based on series which excluded repeat surgery.[57,88] Previously treated myelopathy is under-researched, but the pre-clinical regenerative capacity is anticipated to be different, as are the surgical response and appropriate MCIDs. Patients who underwent surgery for DCM in the past are thus excluded.

In addition, a broad range of secondary endpoints have been included. These assessments have been selected to capture the far-ranging disability experienced by people with DCM. It includes the evaluation of promising objective, quantitative measures, such as microstructural MRI,[89] respiratory physiology,[74,90] GRASSP-Myelopathy (adapted from GRASSP[91]) and gait-laboratory analysis.[92,93] It also includes an assessment of carer quality of life for the first time in a DCM trial.[14] It is recognised that these additional assessments increase the time requirements on participants and investigators, and therefore only a fraction are defined as per protocol. The identification and establishment of improved assessment measures would be of value to future trials and clinical practice.

For peer review only - http://bmjopen.bmj.com/site/about/guidelines.xhtml

Summary

RECEDE-Myelopathy will evaluate the efficacy of Ibudilast, as adjuvant treatment, to improve recovery after surgical decompression in DCM. It is the first regenerative medicine trial in DCM, and the first DCM trial to directly target all the recovery priorities identified by sufferers.

Ethical approval and dissemination.

The RECEDE-Myelopathy trial protocol version 2, 11 March 2020, informed consent forms and all other relevant trial documents have been approved by Central London Research and Ethics Committee (REC), reference 20/LO/0185. HRA approval from HRACW was received on 01/07/2020.. Annual reports will be submitted to the REC in accordance with local national requirements. Trial will be performed following GCP from the ICH guidelines and the letter of the Declaration of Helsinki, as well as any other local regulatory requirements and laws.

All enrolled subjects will have the capacity to consent for the trial and can withdraw from the study at any point. Consent will be obtained by the research team and confirmation of consent to continue partaking in the study will be done on every trial visit.

Dissemination of outcomes and findings from the study with patient involvement

We intend to involve Patients with DCM in the dissemination of research output, both in the production of scientific and lay material, and its communication. Finally, we are currently evaluating the use of PPI representatives to communicate findings to professional audiences. The results of the study will also be presented at international scientific conferences and in peer-reviewed journals regardless of the trial outcome.

For peer review only

Footnotes:

Contributors:
The idea and project were conceived by MRK and BD. The initial draft of the manuscript was created by BD, OM, and circulated amongst other authors for critical revisions. Critical revision and protocol manuscript amendments were collated and executed by SY. BD,OM,SY,DA,SB,MN,PK,LW,JB,SC,SL,MB,MCP,MS,IS,LS,SKR,AC,RT,MW,DC,IW,MGF,PJ,MRK have approved the final version of the manuscript and have been involved in the critical revision of the manuscript. The Chief investigator for RECEDE is MRK and co-investigator is BD. As guarantors are acting MRK, BD and SY.

Competing Interest:
MRK holds a research grant from clinical scientist award and has support for the study from Medicinova. MCP holds research grant award with the NIHR. BD holds research grants with NIHR HTA POLYFIX-DCM, Evelyn Trust (DCM – COINs) and award from National Lottary UK for developing a peer-to-peer support community for Degenerative Cervical myelopathy.BD is also a founder of MoveMed LTD. PJAH holds NIHR research grants.

Funding
Clinician Scientist Award, CS-2015-15-023, supported by the National Institute for Health Research

Ethics Approval:
IRAS No:213009 Ethics Committee : London Central Research Ethics Committee.

For peer review only

References

1. Davies BM, Mowforth OD, Smith EK, Kotter MR. Degenerative cervical myelopathy. *BMJ*. 2018;360:k186. doi:10.1136/bmj.k186

2. Kadanka Z, Adamova B, Kerkovsky M, et al. Predictors of symptomatic myelopathy in degenerative cervical spinal cord compression. *Brain Behav*. 2017;7(9). doi:10.1002/brb3.797

3. Kovalova I, Kerkovsky M, Kadanka Z, et al. Prevalence and Imaging Characteristics of Nonmyelopathic and Myelopathic Spondylotic Cervical Cord Compression. *Spine*. 2016;41(24):1908-1916. doi:10.1097/BRS.0000000000001842

4. Smith SS, Stewart ME, Davies BM, Kotter MRN. The Prevalence of Asymptomatic and Symptomatic Spinal Cord Compression on Magnetic Resonance Imaging: A Systematic Review and Meta-analysis. *Global Spine J*. 2021;11(4):597-607. doi:10.1177/2192568220934496

5. Akter F, Kotter M. Pathobiology of Degenerative Cervical Myelopathy. *Neurosurg Clin N Am*. 2018;29(1):13-19. doi:10.1016/j.nec.2017.09.015

6. Nouri A, Tetreault L, Singh A, Karadimas SK, Fehlings MG. Degenerative Cervical Myelopathy: Epidemiology, Genetics, and Pathogenesis. *Spine*. 2015;40(12):E675-693. doi:10.1097/BRS.0000000000000913

7. Grodzinski B, Durham R, Mowforth O, Stubbs D, Kotter MRN, Davies BM. The effect of ageing on presentation, management and outcomes in degenerative cervical myelopathy: a systematic review. *Age Ageing*. Published online November 19, 2020. doi:10.1093/ageing/afaa236

8. Davies BM, Munro CF, Kotter MR. A Novel Insight Into the Challenges of Diagnosing Degenerative Cervical Myelopathy Using Web-Based Symptom Checkers. *J Med Internet Res*. 2019;21(1):e10868. doi:10.2196/10868

9. Boerger T, Alsouhibani A, Mowforth O, et al. Moving Beyond the Neck and Arm: The Pain Experience of People With Degenerative Cervical Myelopathy Who Have Pain. *Global Spine J*. Published online February 25, 2021:2192568220986143. doi:10.1177/2192568220986143

10. Davies B, Mowforth O, Sadler I, et al. Recovery priorities in degenerative cervical myelopathy: a cross-sectional survey of an international, online community of patients. *BMJ Open*. 2019;9(10):e031486. doi:10.1136/bmjopen-2019-031486

11. Mowforth OD, Davies BM, Kotter MR. “I am not delusional!” Sensory dysaesthesia secondary to degenerative cervical myelopathy. *BMJ Case Rep*. 2019;12(4). doi:10.1136/bcr-2018-229033

12. Oh T, Lafage R, Lafage V, et al. Comparing Quality of Life in Cervical Spondylotic Myelopathy with Other Chronic Debilitating Diseases Using the SF-36 Survey. *World Neurosurg*. Published online January 5, 2017. doi:10.1016/j.wneu.2016.12.124

For peer review only - http://bmjopen.bmj.com/site/about/guidelines.xhtml

13. Hirayama Y, Mowforth OD, Davies BM, Kotter MRN. Determinants of quality of life in degenerative cervical myelopathy: a systematic review. *Br J Neurosurg*. Published online November 18, 2021:1-11. doi:10.1080/02688697.2021.1999390

14. Mowforth OD, Davies BM, Kotter MR. Quality of Life Among Informal Caregivers of Patients With Degenerative Cervical Myelopathy: Cross-Sectional Questionnaire Study. *Interact J Med Res*. 2019;8(4):e12381. doi:10.2196/12381

15. Witiw CD, Tetreault LA, Smieliauskas F, Kopjar B, Massicotte EM, Fehlings MG. Surgery for degenerative cervical myelopathy: a patient-centered quality of life and health economic evaluation. *Spine J*. 2017;17(1):15-25. doi:10.1016/j.spinee.2016.10.015

16. Fehlings MG, Tetreault LA, Riew KD, et al. A Clinical Practice Guideline for the Management of Patients With Degenerative Cervical Myelopathy: Recommendations for Patients With Mild, Moderate, and Severe Disease and Nonmyelopathic Patients With Evidence of Cord Compression. *Global Spine Journal*. 2017;7(3_suppl):70S-83S. doi:10.1177/2192568217701914

17. Pope DH, Mowforth OD, Davies BM, Kotter MRN. Diagnostic Delays Lead To Greater Disability In Degenerative Cervical Myelopathy and Represent A Health-Inequality. *Spine*. Published online October 28, 2019. doi:10.1097/BRS.0000000000003305

18. Sun J, Nan G. The Mitogen-Activated Protein Kinase (MAPK) Signaling Pathway as a Discovery Target in Stroke. *J Mol Neurosci*. 2016;59(1):90-98. doi:10.1007/s12031-016-0717-8

19. Lu M, Wang Y, Zhan X. The MAPK Pathway-Based Drug Therapeutic Targets in Pituitary Adenomas. *Front Endocrinol (Lausanne)*. 2019;10:330. doi:10.3389/fendo.2019.00330

20. Nikulina E, Tidwell JL, Dai HN, Bregman BS, Filbin MT. The phosphodiesterase inhibitor rolipram delivered after a spinal cord lesion promotes axonal regeneration and functional recovery. *Proc Natl Acad Sci U S A*. 2004;101(23):8786-8790. doi:10.1073/pnas.0402595101

21. Pearse DD, Pereira FC, Marcillo AE, et al. cAMP and Schwann cells promote axonal growth and functional recovery after spinal cord injury. *Nat Med*. 2004;10(6):610-616. doi:10.1038/nm1056

22. Syed YA, Baer A, Hofer MP, et al. Inhibition of phosphodiesterase-4 promotes oligodendrocyte precursor cell differentiation and enhances CNS remyelination. *EMBO Mol Med*. 2013;5(12):1918-1934. doi:10.1002/emmm.201303123

23. Gibson LCD, Hastings SF, McPhee I, et al. The inhibitory profile of Ibudilast against the human phosphodiesterase enzyme family. *Eur J Pharmacol*. 2006;538(1-3):39-42. doi:10.1016/j.ejphar.2006.02.053

24. Smith FD, Langeberg LK, Cellurale C, et al. AKAP-Lbc enhances cyclic AMP control of the ERK1/2 cascade. *Nat Cell Biol*. 2010;12(12):1242-1249. doi:10.1038/ncb2130

25. Cuadrado A, Nebreda AR. Mechanisms and functions of p38 MAPK signalling. *Biochem J*. 2010;429(3):403-417. doi:10.1042/BJ20100323

For peer review only - http://bmjopen.bmj.com/site/about/guidelines.xhtml

26. Yamamoto S, Kurokawa R, Kim P. Cilostazol, a selective Type III phosphodiesterase inhibitor: prevention of cervical myelopathy in a rat chronic compression model. *J Neurosurg Spine*. 2014;20(1):93-101. doi:10.3171/2013.9.SPINE121136

27. Badhiwala JH, Ahuja CS, Akbar MA, et al. Degenerative cervical myelopathy - update and future directions. *Nat Rev Neurol*. 2020;16(2):108-124. doi:10.1038/s41582-019-0303-0

28. Akter F, Yu X, Qin X, et al. The Pathophysiology of Degenerative Cervical Myelopathy and the Physiology of Recovery Following Decompression. *Front Neurosci*. 2020;14:138. doi:10.3389/fnins.2020.00138

29. Dhillon RS, Parker J, Syed YA, et al. Axonal plasticity underpins the functional recovery following surgical decompression in a rat model of cervical spondylotic myelopathy. *Acta Neuropathol Commun*. 2016;4(1):89. doi:10.1186/s40478-016-0359-7

30. Ito T, Oyanagi K, Takahashi H, Takahashi HE, Ikuta F. Cervical spondylotic myelopathy. Clinicopathologic study on the progression pattern and thin myelinated fibers of the lesions of seven patients examined during complete autopsy. *Spine (Phila Pa 1976)*. 1996;21(7):827-833. doi:10.1097/00007632-199604010-00010

31. Karadimas SK, Moon ES, Yu WR, et al. A novel experimental model of cervical spondylotic myelopathy (CSM) to facilitate translational research. *Neurobiol Dis*. 2013;54:43-58. doi:10.1016/j.nbd.2013.02.013

32. Karadimas SK, Gatzounis G, Fehlings MG. Pathobiology of cervical spondylotic myelopathy. *Eur Spine J*. 2015;24 Suppl 2:132-138. doi:10.1007/s00586-014-3264-4

33. Ji RR, Berta T, Nedergaard M. Glia and pain: is chronic pain a gliopathy? *Pain*. 2013;154 Suppl 1:S10-S28. doi:10.1016/j.pain.2013.06.022

34. Kim HK, Hwang SH, Oh E, Abdi S. Rolipram, a Selective Phosphodiesterase 4 Inhibitor, Ameliorates Mechanical Hyperalgesia in a Rat Model of Chemotherapy-Induced Neuropathic Pain through Inhibition of Inflammatory Cytokines in the Dorsal Root Ganglion. *Front Pharmacol*. 2017;8:885. doi:10.3389/fphar.2017.00885

35. Kim HK, Kwon JY, Yoo C, Abdi S. The Analgesic Effect of Rolipram, a Phosphodiesterase 4 Inhibitor, on Chemotherapy-Induced Neuropathic Pain in Rats. *Anesth Analg*. 2015;121(3):822-828. doi:10.1213/ANE.0000000000000853

36. Kostich W, Hamman BD, Li YW, et al. Inhibition of AAK1 Kinase as a Novel Therapeutic Approach to Treat Neuropathic Pain. *J Pharmacol Exp Ther*. 2016;358(3):371-386. doi:10.1124/jpet.116.235333

37. Espírito-Santo RF, Meira CS, Costa RDS, et al. The anti-inflammatory and immunomodulatory potential of braylin: Pharmacological properties and mechanisms by in silico, in vitro and in vivo approaches. *PLoS One*. 2017;12(6):e0179174. doi:10.1371/journal.pone.0179174

38. Bao F, Fleming JC, Golshani R, et al. A selective phosphodiesterase-4 inhibitor reduces leukocyte infiltration, oxidative processes, and tissue damage after spinal cord injury. *J Neurotrauma*. 2011;28(6):1035-1049. doi:10.1089/neu.2010.1575

For peer review only

39. Francischi JN, Yokoro CM, Poole S, Tafuri WL, Cunha FQ, Teixeira MM. Anti-inflammatory and analgesic effects of the phosphodiesterase 4 inhibitor rolipram in a rat model of arthritis. *Eur J Pharmacol*. 2000;399(2-3):243-249. doi:10.1016/s0014-2999(00)00330-7

40. Bielekova B, Richert N, Howard T, et al. Treatment with the phosphodiesterase type-4 inhibitor rolipram fails to inhibit blood--brain barrier disruption in multiple sclerosis. *Mult Scler*. 2009;15(10):1206-1214. doi:10.1177/1352458509345903

41. Rolan P, Hutchinson M, Johnson K. Ibudilast: a review of its pharmacology, efficacy and safety in respiratory and neurological disease. *Expert Opin Pharmacother*. 2009;10(17):2897-2904. doi:10.1517/14656560903426189

42. Brooks B, Bravver E, Sanjak M, et al. A Single-Center, Randomized, Double-Blind, Placebo-Controlled, Six-Month Clinical Trial Followed by an Open-Label Extension to Evaluate the Safety, Tolerability and Clinical Endpoint Responsiveness of the Phosphodiesterase Type 4 (PDE4) Inhibitor Ibudilast (MN-166) in Subjects with Amyotrophic Lateral Sclerosis (ALS) - STEP-IBUDILAST-ALS-DB-OLE-1 (I8-1B). *Neurology*. 2015;84(14 Supplement). Accessed December 2, 2021. https://n.neurology.org/content/84/14_Supplement/I8-1B

43. Fox RJ, Coffey CS, Cudkowicz ME, et al. Design, rationale, and baseline characteristics of the randomized double-blind phase II clinical trial of ibudilast in progressive multiple sclerosis. *Contemp Clin Trials*. 2016;50:166-177. doi:10.1016/j.cct.2016.08.009

44. Brooks B, Bravver EK, Sanjak M, et al. Ibudilast - Phosphodiesterase Type 4 Inhibitor - Bi-Modal Therapy with Riluzole in Early [Not Requiring Non-Invasive Ventilation (NIV)] Cohort (EC) and Advanced [Requiring NIV] (ANC) Amyotrophic Lateral Sclerosis (ALS) Patients - Single-Center Adaptive Design Six-Month Double-Blind (DB) - Placebo-Controlled Phase 1b/2a Epoch Followed by Six-Month Open Label Extension (OLE) Epoch, Washout (WO) and Post-Washout Epoch (PWO) – Final Report and Future Directions (P6.465). *Neurology*. 2018;90(15 Supplement). Accessed December 2, 2021. https://n.neurology.org/content/90/15_Supplement/P6.465

45. Brooks B, Bravver E, Sanjak M, et al. Adaptive Design Single Center Phosphodiesterase Type 4 (PDE4) Inhibitor - Ibudilast (MN-166-ALS-1201) Phase 1b/2a Clinical Trial Double-Blind (DB) with Open Label Extensions (OLE) [NCT02238626] for Amyotrophic Lateral Sclerosis (ALS) Patients [1] Not Requiring Non-Invasive Ventilation (no-NIV) up to 5 Years (Early Cohort-EC) and [2] Requiring Non-Invasive Ventilation (NIV) up to 10 Years(Advanced NIV Cohort-ANC) from Disease Onset - Behavior of Creatinine as a Biomarker in Short Clinical Trials (P5.003). *Neurology*. 2016;86(16 Supplement). Accessed December 2, 2021. https://n.neurology.org/content/86/16_Supplement/P5.003

46. Fox RJ, Coffey CS, Conwit R, et al. Phase 2 Trial of Ibudilast in Progressive Multiple Sclerosis. *New England Journal of Medicine*. 2018;379(9):846-855. doi:10.1056/NEJMoa1803583

57. Fehlings MG, Ibrahim A, Tetreault L, et al. A global perspective on the outcomes of surgical decompression in patients with cervical spondylotic myelopathy: results from the

For peer review only - http://bmjopen.bmj.com/site/about/guidelines.xhtml

prospective multicenter AOSpine international study on 479 patients. *Spine*. 2015;40(17):1322-1328. doi:10.1097/BRS.0000000000000988

58. Fehlings MG, Wilson JR, Karadimas SK, Arnold PM, Kopjar B. Clinical evaluation of a neuroprotective drug in patients with cervical spondylotic myelopathy undergoing surgical treatment: design and rationale for the CSM-Protect trial. *Spine*. 2013;38(22 Suppl 1):S68-75. doi:10.1097/BRS.0b013e3182a7e9b0

59. Kopjar B, Tetreault L, Kalsi-Ryan S, Fehlings M. Psychometric properties of the modified Japanese Orthopaedic Association scale in patients with cervical spondylotic myelopathy. *Spine (Phila Pa 1976)*. 2015;40(1):E23-28. doi:10.1097/BRS.0000000000000648

60. Fehlings MG, Badhiwala JH, Ahn H, et al. Safety and efficacy of riluzole in patients undergoing decompressive surgery for degenerative cervical myelopathy (CSM-Protect): a multicentre, double-blind, placebo-controlled, randomised, phase 3 trial. *Lancet Neurol*. 2021;20(2):98-106. doi:10.1016/S1474-4422(20)30407-5

61. Moon ES, Karadimas SK, Yu WR, Austin JW, Fehlings MG. Riluzole attenuates neuropathic pain and enhances functional recovery in a rodent model of cervical spondylotic myelopathy. *Neurobiol Dis*. 2014;62:394-406. doi:10.1016/j.nbd.2013.10.020

62. Segerdahl M. Pain outcome variables--a never ending story? *Pain*. 2011;152(5):961-962. doi:10.1016/j.pain.2011.03.008

63. Davies BM, McHugh M, Elgheriani A, et al. Reported Outcome Measures in Degenerative Cervical Myelopathy: A Systematic Review. *PLoS One*. 2016;11(8). doi:10.1371/journal.pone.0157263

64. MacDowall A, Skeppholm M, Robinson Y, Olerud C. Validation of the visual analog scale in the cervical spine. *J Neurosurg Spine*. 2018;28(3):227-235. doi:10.3171/2017.5.SPINE1732

65. Parker SL, Godil SS, Shau DN, Mendenhall SK, McGirt MJ. Assessment of the minimum clinically important difference in pain, disability, and quality of life after anterior cervical discectomy and fusion: clinical article. *J Neurosurg Spine*. 2013;18(2):154-160. doi:10.3171/2012.10.SPINE12312

51. Davies BM, Khan DZ, Mowforth OD, et al. RE-CODE DCM (REsearch Objectives and Common Data Elements for Degenerative Cervical Myelopathy): A Consensus Process to Improve Research Efficiency in DCM, Through Establishment of a Standardized Dataset for Clinical Research and the Definition of the Research Priorities. *Global Spine J*. 2019;9(1 Suppl):65S-76S. doi:10.1177/2192568219832855

65. Brouwer WBF, van Exel NJA, van Gorp B, Redekop WK. The CarerQol instrument: a new instrument to measure care-related quality of life of informal caregivers for use in economic evaluations. *Qual Life Res*. 2006;15(6):1005-1021. doi:10.1007/s11136-005-5994-6

66. Tetreault L, Nouri A, Kopjar B, Côté P, Fehlings MG. The Minimum Clinically Important Difference of the Modified Japanese Orthopaedic Association Scale in Patients

For peer review only - http://bmjopen.bmj.com/site/about/guidelines.xhtml

with Degenerative Cervical Myelopathy. *Spine (Phila Pa 1976)*. 2015;40(21):1653-1659. doi:10.1097/BRS.0000000000001127

67. Tetreault L, Wilson JR, Kotter MRN, et al. Predicting the minimum clinically important difference in patients undergoing surgery for the treatment of degenerative cervical myelopathy. *Neurosurg Focus*. 2016;40(6):E14. doi:10.3171/2016.3.FOCUS1665

68. Tetreault L, Lange SF, Chotai S, et al. A Systematic Review of Definitions for Neurological Complications and Disease Progression in Patients Treated Surgically for Degenerative Cervical Myelopathy. *Spine (Phila Pa 1976)*. 2019;44(18):1318-1331. doi:10.1097/BRS.0000000000003066

69. Bretz F, Maurer W, Brannath W, Posch M. A graphical approach to sequentially rejective multiple test procedures. *Statistics in Medicine*. 2009;28(4):586-604. doi:10.1002/sim.3495

70. Benjamini Y, Hochberg Y. Controlling the False Discovery Rate: A Practical and Powerful Approach to Multiple Testing. *Journal of the Royal Statistical Society: Series B (Methodological)*. 1995;57(1):289-300. doi:10.1111/j.2517-6161.1995.tb02031.x

47. Chalmers I, Bracken MB, Djulbegovic B, et al. How to increase value and reduce waste when research priorities are set. *Lancet*. 2014;383(9912):156-165. doi:10.1016/S0140-6736(13)62229-1

48. Ergina PL, Cook JA, Blazeby JM, et al. Challenges in evaluating surgical innovation. *Lancet*. 2009;374(9695):1097-1104. doi:10.1016/S0140-6736(09)61086-2

49. Davies BM, Munro C, Khan DZ, et al. Outcomes of Degenerative Cervical Myelopathy From The Perspective of Persons Living With the Condition: Findings of a Semistructured Interview Process With Partnered Internet Survey. *Global Spine J*. Published online November 18, 2020:2192568220953811. doi:10.1177/2192568220953811

50. Davies BM, Mowforth OD, Khan DZ, et al. The development of lived experience-centered word clouds to support research uncertainty gathering in degenerative cervical myelopathy: results from an engagement process and protocol for their evaluation, via a nested randomized controlled trial. *Trials*. 2021;22(1):415. doi:10.1186/s13063-021-05349-8

52. Mowforth OD, Davies BM, Goh S, O'Neill CP, Kotter MRN. Research Inefficiency in Degenerative Cervical Myelopathy: Findings of a Systematic Review on Research Activity Over the Past 20 Years. *Global Spine J*. 2020;10(4):476-485. doi:10.1177/2192568219847439

53. Grodzinski B, Bestwick H, Bhatti F, et al. Research activity amongst DCM research priorities. *Acta Neurochir (Wien)*. Published online February 24, 2021. doi:10.1007/s00701-021-04767-6

54. Neurology TL. A focus on patient outcomes in cervical myelopathy. *The Lancet Neurology*. 2019;18(7):615. doi:10.1016/S1474-4422(19)30168-1

For peer review only

55. Mowforth OD, Davies BM, Kotter MR. The Use of Smart Technology in an Online Community of Patients With Degenerative Cervical Myelopathy. *JMIR Form Res*. 2019;3(2):e11364. doi:10.2196/11364

56. Davies B, Kotter M. Lessons From Recruitment to an Internet-Based Survey for Degenerative Cervical Myelopathy: Comparison of Free and Fee-Based Methods. *JMIR Res Protoc*. 2018;7(2):e18. doi:10.2196/resprot.6567

72. Waqar M, Wilcock J, Garner J, Davies B, Kotter M. Quantitative analysis of medical students' and physicians' knowledge of degenerative cervical myelopathy. *BMJ Open*. 2020;10(1):e028455. doi:10.1136/bmjopen-2018-028455

73. Kalsi-Ryan S, Rienmueller AC, Riehm L, et al. Quantitative Assessment of Gait Characteristics in Degenerative Cervical Myelopathy: A Prospective Clinical Study. *J Clin Med*. 2020;9(3):E752. doi:10.3390/jcm9030752

74. Bhagavatula ID, Bhat DI, Sasidharan GM, et al. Subclinical respiratory dysfunction in chronic cervical cord compression: a pulmonary function test correlation. *Neurosurg Focus*. 2016;40(6):E3. doi:10.3171/2016.3.FOCUS1647

75. Pope DH, Davies BM, Mowforth OD, Bowden AR, Kotter MRN. Genetics of Degenerative Cervical Myelopathy: A Systematic Review and Meta-Analysis of Candidate Gene Studies. *J Clin Med*. 2020;9(1). doi:10.3390/jcm9010282

76. Badran A, Davies BM, Bailey HM, Kalsi-Ryan S, Kotter MR. Is there a role for postoperative physiotherapy in degenerative cervical myelopathy? A systematic review. *Clin Rehabil*. Published online April 1, 2018:269215518766229. doi:10.1177/0269215518766229

77. Davies BM, McHugh M, Elgheriani A, et al. The reporting of study and population characteristics in degenerative cervical myelopathy: A systematic review. *PLOS ONE*. 2017;12(3):e0172564. doi:10.1371/journal.pone.0172564

78. Bogduk N, Govind J. Cervicogenic headache: an assessment of the evidence on clinical diagnosis, invasive tests, and treatment. *Lancet Neurol*. 2009;8(10):959-968. doi:10.1016/S1474-4422(09)70209-1

79. Sheather-Reid RB, Cohen ML. Psychophysical evidence for a neuropathic component of chronic neck pain. *Pain*. 1998;75(2-3):341-347. doi:10.1016/s0304-3959(98)00013-x

80. Voerman VF, van Egmond J, Crul BJ. Elevated detection thresholds for mechanical stimuli in chronic pain patients: support for a central mechanism. *Arch Phys Med Rehabil*. 2000;81(4):430-435. doi:10.1053/mr.2000.3777

81. Zaproudina N, Ming† Z, Närhi M. Sensory and sympathetic disorders in chronic non-specific neck pain. *Funct Neurol*. 2015;30(3):165-171. doi:10.11138/FNeur/2015.30.3.165

82. Westermann A, Rönnau AK, Krumova E, et al. Pain-associated mild sensory deficits without hyperalgesia in chronic non-neuropathic pain. *Clin J Pain*. 2011;27(9):782-789. doi:10.1097/AJP.0b013e31821d8fce

For peer review only

83. Blyufer A, Lhamo S, Tam C, Tariq I, Thavornwatanayong T, Mahajan SS. Riluzole: A neuroprotective drug with potential as a novel anti-cancer agent (Review). *Int J Oncol*. 2021;59(5):95. doi:10.3892/ijo.2021.5275

84. Lee S, Zhao X, Hatch M, Chun S, Chang E. Central Neuropathic Pain in Spinal Cord Injury. *Crit Rev Phys Rehabil Med*. 2013;25(3-4):159-172. doi:10.1615/CritRevPhysRehabilMed.2013007944

85. Kalsi-Ryan S, Singh A, Massicotte EM, et al. Ancillary outcome measures for assessment of individuals with cervical spondylotic myelopathy. *Spine (Phila Pa 1976)*. 2013;38(22 Suppl 1):S111-122. doi:10.1097/BRS.0b013e3182a7f499

86. Tetreault LA, Côté P, Kopjar B, Arnold P, Fehlings MG, AOSpine North America and International Clinical Trial Research Network. A clinical prediction model to assess surgical outcome in patients with cervical spondylotic myelopathy: internal and external validations using the prospective multicenter AOSpine North American and international datasets of 743 patients. *Spine J*. 2015;15(3):388-397. doi:10.1016/j.spinee.2014.12.145

87. Ahuja CS, Wilson JR, Nori S, et al. Traumatic spinal cord injury. *Nat Rev Dis Primers*. 2017;3(1):1-21. doi:10.1038/nrdp.2017.18

88. Fehlings MG, Wilson JR, Kopjar B, et al. Efficacy and safety of surgical decompression in patients with cervical spondylotic myelopathy: results of the AOSpine North America prospective multi-center study. *J Bone Joint Surg Am*. 2013;95(18):1651-1658. doi:10.2106/JBJS.L.00589

89. Martin AR, Leener BD, Cohen-Adad J, et al. Can microstructural MRI detect subclinical tissue injury in subjects with asymptomatic cervical spinal cord compression? A prospective cohort study. *BMJ Open*. 2018;8(4):e019809. doi:10.1136/bmjopen-2017-019809

90. Toyoda H, Nakamura H, Konishi S, Terai H, Takaoka K. Does chronic cervical myelopathy affect respiratory function? *J Neurosurg Spine*. 2004;1(2):175-178. doi:10.3171/spi.2004.1.2.0175

91. Kalsi-Ryan S, Beaton D, Ahn H, et al. Responsiveness, Sensitivity, and Minimally Detectable Difference of the Graded and Redefined Assessment of Strength, Sensibility, and Prehension, Version 1.0. *J Neurotrauma*. 2016;33(3):307-314. doi:10.1089/neu.2015.4217

92. McDermott A, Bolger C, Keating L, McEvoy L, Meldrum D. Reliability of three-dimensional gait analysis in cervical spondylotic myelopathy. *Gait Posture*. 2010;32(4):552-558. doi:10.1016/j.gaitpost.2010.07.019

93. Nagai T, Takahashi Y, Endo K, Ikegami R, Ueno R, Yamamoto K. Analysis of spastic gait in cervical myelopathy: Linking compression ratio to spatiotemporal and pedobarographic parameters. *Gait Posture*. 2018;59:152-156. doi:10.1016/j.gaitpost.2017.10.013

For peer review only - http://bmjopen.bmj.com/site/about/guidelines.xhtml

Figure Legend.

Figure 1: Trial Flow Chart.
Eligible and consenting participants will be randomised to an intervention or control arm and followed up for 12 months after surgery.

For peer review only

For peer review only - http://bmjopen.bmj.com/site/about/guidelines.xhtml

Figure 1. Trial Flow Chart.

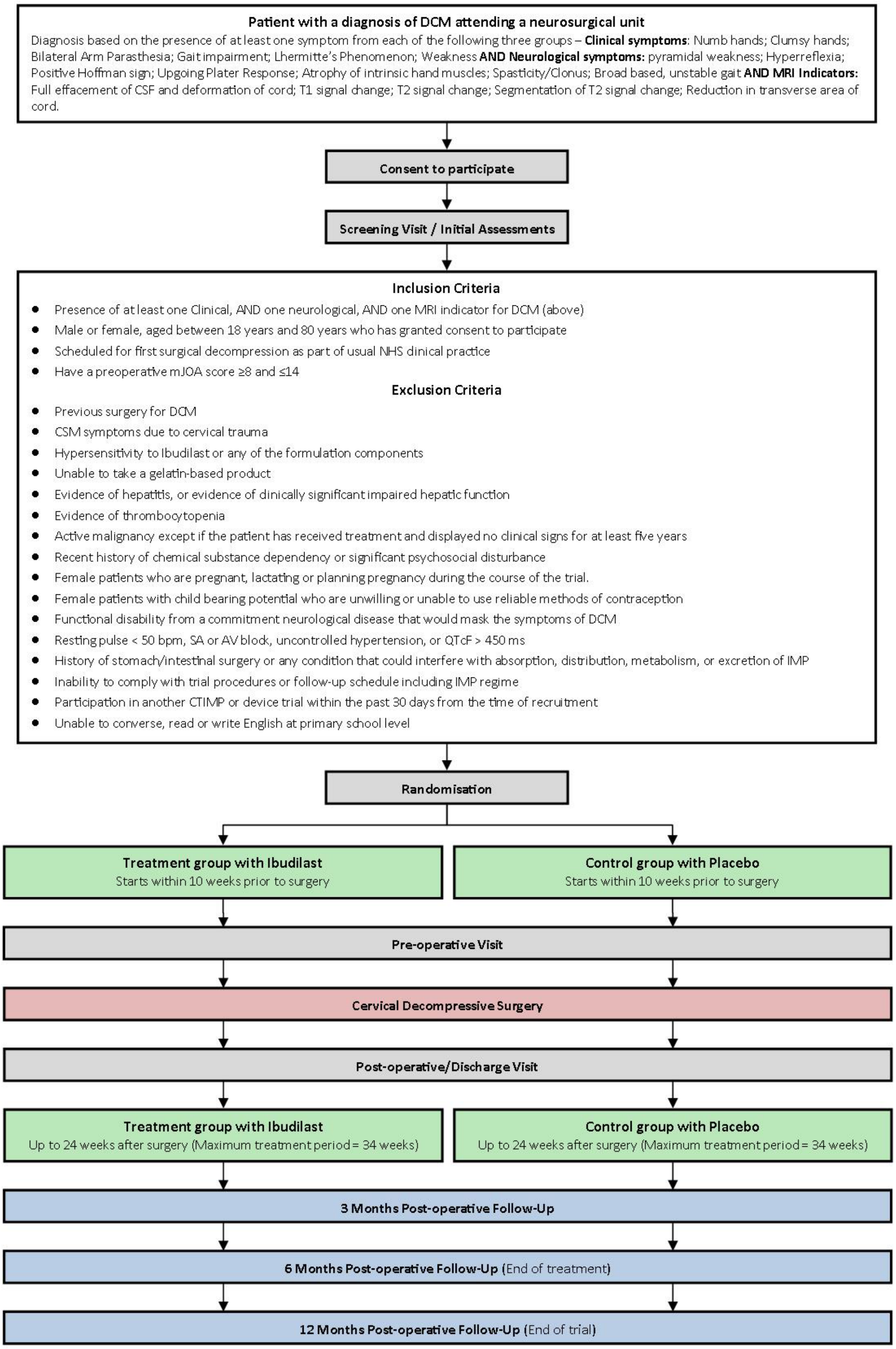

SPIRIT

STANDARD PROTOCOL ITEMS: RECOMMENDATIONS FOR INTERVENTIONAL TRIALS

SPIRIT 2013 Checklist: Recommended items to address in a clinical trial protocol and related documents*

Section/item	Item No	Description
**Administrative information**		
Title	1	Descriptive title identifying the study design, population, interventions, and, if applicable, trial acronym – Present, Page 1
Trial registration	2a	Trial identifier and registry name. If not yet registered, name of intended registry – Present, Page 3 in Ethics and Dissemination Section.
	2b	All items from the World Health Organization Trial Registration Data Set – Present throughout the Manuscript.
Protocol version	3	Date and version identifier – Present, Page 22, Footnotes
Funding	4	Sources and types of financial, material, and other support - Present
Roles and responsibilities	5a	Names, affiliations, and roles of protocol contributors -Present, Page 1
	5b	Name and contact information for the trial sponsor – Present, Present Page 1,2
	5c	Role of study sponsor and funders, if any, in study design; collection, management, analysis, and interpretation of data; writing of the report; and the decision to submit the report for publication, including whether they will have ultimate authority over any of these activities – N/a
	5d	Composition, roles, and responsibilities of the coordinating centre, steering committee, endpoint adjudication committee, data management team, and other individuals or groups overseeing the trial, if applicable (see Item 21a for data monitoring committee) – Present, Page 15 and 16
**Introduction**		
Background and rationale	6a	Description of research question and justification for undertaking the trial, including summary of relevant studies (published and unpublished) examining benefits and harms for each intervention – Present, pages 4-7
	6b	Explanation for choice of comparators – Present, page 12-14
Objectives	7	Specific objectives or hypotheses -Present, Page 12

For peer review only

Trial design	8	Description of trial design including type of trial (eg, parallel group, crossover, factorial, single group), allocation ratio, and framework (eg, superiority, equivalence, noninferiority, exploratory) – Present. Page 8

Methods: Participants, interventions, and outcomes

Study setting	9	Description of study settings (eg, community clinic, academic hospital) and list of countries where data will be collected. Reference to where list of study sites can be obtained – Present, page 8
Eligibility criteria	10	Inclusion and exclusion criteria for participants. If applicable, eligibility criteria for study centres and individuals who will perform the interventions (eg, surgeons, psychotherapists) – Present, page 9/10
Interventions	11a	Interventions for each group with sufficient detail to allow replication, including how and when they will be administered – Present, page 11
	11b	Criteria for discontinuing or modifying allocated interventions for a given trial participant (eg, drug dose change in response to harms, participant request, or improving/worsening disease) – Present, page 11
	11c	Strategies to improve adherence to intervention protocols, and any procedures for monitoring adherence (eg, drug tablet return, laboratory tests) – n/a
	11d	Relevant concomitant care and interventions that are permitted or prohibited during the trial – n/a
Outcomes	12	Primary, secondary, and other outcomes, including the specific measurement variable (eg, systolic blood pressure), analysis metric (eg, change from baseline, final value, time to event), method of aggregation (eg, median, proportion), and time point for each outcome. Explanation of the clinical relevance of chosen efficacy and harm outcomes is strongly recommended – Present , Page 12-14
Participant timeline	13	Time schedule of enrolment, interventions (including any run-ins and washouts), assessments, and visits for participants. A schematic diagram is highly recommended (see Figure) – Present, page 11
Sample size	14	Estimated number of participants needed to achieve study objectives and how it was determined, including clinical and statistical assumptions supporting any sample size calculations – Present, page 14
Recruitment	15	Strategies for achieving adequate participant enrolment to reach target sample size – N/a

Methods: Assignment of interventions (for controlled trials)

Allocation:

For peer review only - http://bmjopen.bmj.com/site/about/guidelines.xhtml

Sequence generation	16a	Method of generating the allocation sequence (eg, computer-generated random numbers), and list of any factors for stratification. To reduce predictability of a random sequence, details of any planned restriction (eg, blocking) should be provided in a separate document that is unavailable to those who enrol participants or assign interventions – Present, page 11, Enrolment and Randomisation Section
Allocation concealment mechanism	16b	Mechanism of implementing the allocation sequence (eg, central telephone; sequentially numbered, opaque, sealed envelopes), describing any steps to conceal the sequence until interventions are assigned – Present, page 11, Enrolment and Randomisation Section
Implementation	16c	Who will generate the allocation sequence, who will enrol participants, and who will assign participants to interventions – Present
Blinding (masking)	17a	Who will be blinded after assignment to interventions (eg, trial participants, care providers, outcome assessors, data analysts), and how – Present, Page 11
	17b	If blinded, circumstances under which unblinding is permissible, and procedure for revealing a participant's allocated intervention during the trial – Present, Page 11
**Methods: Data collection, management, and analysis**		
Data collection methods	18a	Plans for assessment and collection of outcome, baseline, and other trial data, including any related processes to promote data quality (eg, duplicate measurements, training of assessors) and a description of study instruments (eg, questionnaires, laboratory tests) along with their reliability and validity, if known. Reference to where data collection forms can be found, if not in the protocol – Present, 14-17
	18b	Plans to promote participant retention and complete follow-up, including list of any outcome data to be collected for participants who discontinue or deviate from intervention protocols – N/a
Data management	19	Plans for data entry, coding, security, and storage, including any related processes to promote data quality (eg, double data entry; range checks for data values). Reference to where details of data management procedures can be found, if not in the protocol – page 15
Statistical methods	20a	Statistical methods for analysing primary and secondary outcomes. Reference to where other details of the statistical analysis plan can be found, if not in the protocol – 16-17
	20b	Methods for any additional analyses (eg, subgroup and adjusted analyses) – Present, 16-17

For peer review only - http://bmjopen.bmj.com/site/about/guidelines.xhtml

	20c	Definition of analysis population relating to protocol non-adherence (eg, as randomised analysis), and any statistical methods to handle missing data (eg, multiple imputation) – Present , page 16-17
**Methods: Monitoring**		
Data monitoring	21a	Composition of data monitoring committee (DMC); summary of its role and reporting structure; statement of whether it is independent from the sponsor and competing interests; and reference to where further details about its charter can be found, if not in the protocol. Alternatively, an explanation of why a DMC is not needed – Present, page 15-16
	21b	Description of any interim analyses and stopping guidelines, including who will have access to these interim results and make the final decision to terminate the trial – Present, Page 16-17
Harms	22	Plans for collecting, assessing, reporting, and managing solicited and spontaneously reported adverse events and other unintended effects of trial interventions or trial conduct – Present, page 16-17
Auditing	23	Frequency and procedures for auditing trial conduct, if any, and whether the process will be independent from investigators and the sponsor – Present, page 16-17
**Ethics and dissemination**		
Research ethics approval	24	Plans for seeking research ethics committee/institutional review board (REC/IRB) approval – REC Approval gained, page 21
Protocol amendments	25	Plans for communicating important protocol modifications (eg, changes to eligibility criteria, outcomes, analyses) to relevant parties (eg, investigators, REC/IRBs, trial participants, trial registries, journals, regulators) – Present, page 21
Consent or assent	26a	Who will obtain informed consent or assent from potential trial participants or authorised surrogates, and how (see Item 32) – present, page 21
	26b	Additional consent provisions for collection and use of participant data and biological specimens in ancillary studies, if applicable – Present , page 21
Confidentiality	27	How personal information about potential and enrolled participants will be collected, shared, and maintained in order to protect confidentiality before, during, and after the trial – Present, page 21
Declaration of interests	28	Financial and other competing interests for principal investigators for the overall trial and each study site – Present. Page 22

review only

For peer review only - http://bmjopen.bmj.com/site/about/guidelines.xhtml

Access to data	29	Statement of who will have access to the final trial dataset, and disclosure of contractual agreements that limit such access for investigators – Present, page 15
Ancillary and post-trial care	30	Provisions, if any, for ancillary and post-trial care, and for compensation to those who suffer harm from trial participation – n/a
Dissemination policy	31a	Plans for investigators and sponsor to communicate trial results to participants, healthcare professionals, the public, and other relevant groups (eg, via publication, reporting in results databases, or other data sharing arrangements), including any publication restrictions – Present, page 21
	31b	Authorship eligibility guidelines and any intended use of professional writers – Included in the submission
	31c	Plans, if any, for granting public access to the full protocol, participant-level dataset, and statistical code(n/a)
**Appendices**		
Informed consent materials	32	Model consent form and other related documentation given to participants and authorised surrogates – (attached)
Biological specimens	33	Plans for collection, laboratory evaluation, and storage of biological specimens for genetic or molecular analysis in the current trial and for future use in ancillary studies, if applicable – N/a

*It is strongly recommended that this checklist be read in conjunction with the SPIRIT 2013 Explanation & Elaboration for important clarification on the items. Amendments to the protocol should be tracked and dated. The SPIRIT checklist is copyrighted by the SPIRIT Group under the Creative Commons "Attribution-NonCommercial-NoDerivs 3.0 Unported" license.

For peer review only

BMJ Open

Targeting patient recovery priorities in degenerative cervical myelopathy: design and rationale for the RECEDE-Myelopathy trial - Study Protocol

Journal:	*BMJ Open*
Manuscript ID	bmjopen-2022-061294.R2
Article Type:	Protocol
Date Submitted by the Author:	26-Aug-2022
Complete List of Authors:	Davies, Benjamin; University of Cambridge, Department of Clinical Neurosurgery; University of Cambridge<br>Mowforth, Oliver; University of Cambridge, Department of Academic Neurosurgery<br>Yordanov, Stefan; Addenbrooke's Hospital, Neurosurgery<br>Alvarez-Berdugo, Daniel; Cambridge University Hospitals NHS Foundation Trust<br>Bond, Simon; Cambridge University Hospitals NHS Foundation Trust, Cambridge clinical trials unit<br>Nodale, Marianna; Cambridge University Hospitals NHS Foundation Trust<br>Kareclas, Paula; Cambridge University Hospitals NHS Foundation Trust, Cambridge Clinical Trials Unit<br>Whitehead, Lynne; Cambridge University Hospitals NHS Foundation Trust<br>Bishop, Jon; NIHR Surgical Reconstruction and Microbiology Research Centre, Medical Statistician<br>Chandran, Siddharthan; University of Cambridge, Clinical Neurosciences<br>Lamb, Sarah; University of Oxford<br>Bacon, Mark; International Spinal Research Trust<br>Papadopoulos, Marios; St George's Hospital, Department of Neurosurgery London, United Kingdom, Department of Neurosurgery<br>Starkey, Michelle; Myelopathy.org<br>Sadler, Iwan; Myelopathy.org<br>Smith, Lara; Myelopathy.org<br>Kalsi-Ryan, Sukhvinder; Toronto Rehabilitation Institute<br>Carpenter, Adrian; University of Cambridge, Department of Clinical Neurosciences<br>Trivedi, Rikin A.; Cambridge Univ Hosp Fdn Trust<br>Wilby, Martin; The Walton Centre NHS Foundation Trust, Neurosurgery<br>Choi, David; National Hospital for Neurology and Neurosurgery, Department of Neurosurgery<br>Wilkinson, Ian; Division of Experimental Medicine and Immunotherapeutics, Department of Medicine, University of Cambridge; Cambridge Clinical Trials Unit,<br>Fehlings, Michael G.; Toronto Western Hospital<br>Hutchinson, Peter; University of Cambridge, Academic Neurosurgery<br>Kotter, Mark R.N.; University of Cambridge, Department of Clinical Neurosurgery; University of Cambridge
Primary Subject	Neurology

Heading:	
Secondary Subject Heading:	Surgery
Keywords:	NEUROSURGERY, Spine < ORTHOPAEDIC & TRAUMA SURGERY, Neurosurgery < SURGERY

SCHOLARONE™ Manuscripts

Targeting patient recovery priorities in degenerative cervical myelopathy: design and rationale for the RECEDE-Myelopathy trial – Study Protocol

Benjamin M. Davies[1], Oliver D. Mowforth[1], Stefan Yordanov[1], Daniel Alvarez-Berdugo[2], Simon Bond[2], Marianna Nodale[2], Paula Kareclas[2], Lynne Whitehead[3], Jonathan Bishop[4], Siddharthan Chandran[5], Sarah E Lamb[6], Mark Bacon[7], Marios C. Papadopoulos[8], Michelle Starkey[9], Iwan Sadler[9], Lara Smith[9], Sukhivinder Kalsi-Ryan[10], Adrian Carpenter[11], Rikin Trivedi[1], Martin Wilby[12], David Choi[13], Ian Wilkinson[2,14], Michael G Fehlings[15], Peter Hutchinson[1], Mark R.N. Kotter[1,16]

1) Academic Neurosurgery Unit, Department of Clinical Neurosurgery, University of Cambridge, Cambridge, UK
2) Cambridge Clinical Trials Unit, Cambridge University Hospitals NHS Foundation Trust, Cambridge, UK
3) Pharmacy Department, Cambridge University Hospitals NHS Foundation Trust, Cambridge, UK
4) Birmingham Clinical Trials Unit, Institute of Applied Health Research, University of Birmingham, Birmingham, UK
5) Centre for Clinical Brain Sciences, University of Edinburgh, Edinburgh, UK
6) College of Medicine and Health, University of Exeter, UK
7) International Spinal Research Trust, London, UK
8) Academic Neurosurgery Unit, St. George's University of London, London, UK
9) Myelopathy.org, Cambridge, UK
10) Department of Physical Therapy, Krembil Neuroscience Spine Program, Toronto Western Hospital UHN, Toronto, Canada
11) Wolfson Brain Imaging Centre, Department of Clinical Neurosciences, University of Cambridge, Cambridge, UK
12) Department of Neurosurgery, The Walton Centre for Neurology and Neurosurgery NHS Trust, Liverpool, UK
13) Department of Neurosurgery, National Hospital for Neurology and Neurosurgery, University College London Hospitals NHS Foundation, London, UK
14) Division of Experimental Medicine and Immunotherapeutics, University of Cambridge, Cambridge, UK
15) Division of Neurosurgery, Department of Surgery, Toronto Western Hospital, Toronto, Canada
16) WT MRC Cambridge Stem Cell Institute, Anne McLaren Laboratory, University of Cambridge, UK

Key words: cervical; myelopathy; spondylosis; spondylotic; stenosis; disc herniation; ossification posterior longitudinal ligament; degeneration; disability; recovery; questionnaire

Word Count:

Acknowledgements: Research in the senior author's laboratory is supported by the Cambridge NIHR Brain Injury MedTech Cooperative. MRNK is supported by a NIHR Clinician Scientist Award.

For peer review only - http://bmjopen.bmj.com/site/about/guidelines.xhtml

Disclaimer: This report is independent research arising from a Clinician Scientist Award, CS-2015-15-023, supported by the National Institute for Health Research. The views expressed in this publication are those of the authors and not necessarily those of the NHS, the National Institute for Health Research or the Department of Health and Social Care.

Correspondence:
Dr. Mark R.N. Kotter
Department of Clinical Neurosciences
University of Cambridge
Mrk25@medschl.cam.ac.uk

For peer review only

Abstract

Study Design:

Clinical trial protocol v2.2 Oct 2020

Introduction:

Degenerative cervical myelopathy (DCM) is a common and disabling condition of symptomatic cervical spinal cord compression secondary to degenerative changes in spinal structures leading to a mechanical stress injury of the spinal cord. RECEDE-Myelopathy aims to test the disease-modulating activity of the PDE3/4 inhibitor Ibudilast as an adjuvant to surgical decompression in DCM.

Methods and Analysis:

RECEDE-Myelopathy is a multi-centre, double-blind, randomised, placebo-controlled trial. Participants will be randomized to receive either 60-100mg Ibudilast or placebo starting within 10 weeks prior to surgery and continuing for 24 weeks after surgery for a maximum of 34 weeks. Adults with DCM, who have a modified Japanese Orthopaedic Association score (mJOA) 8-14 inclusive and are scheduled for their first decompressive surgery are eligible for inclusion. The co-primary endpoints are pain measured on a visual analogue scale and physical function measured by the mJOA score at 6 months after surgery. Clinical assessments will be undertaken pre-operatively, post-operatively and 3, 6 and 12 months after surgery. We hypothesize that adjuvant therapy with Ibudilast leads to a meaningful and additional improvement in either pain or function, as compared to standard routine care.

Ethics and Dissemination:

Ethical approval has been obtained from HRA – Wales .The results will be presented at an international and national scientific conferences and in a peer-reviewed journals.

ISRCTN Number: ISRCTN16682024

Strengths and Limitations:

- **Significant patient and public involvement in trial design and outcomes planning.**

For peer review only

- **A pragmatic approach to patient inclusion criteria was utilised – all patient with mJOA between 8-14 and MRI findings of DCM who are scheduled for their first surgery for DCM regardless of approach are able to be included.**
- **We will explore and compare both clinical and objective findings and validated questionnaire and multiple patient reported outcomes.**
- **A limitation is the need of close patient follow-up and rigorous screening with additional blood tests to comply with drug monitoring and assessments needed.**

For peer review only

Introduction

Here we present the study rationale and design of RECEDE Myelopathy (**Re**generation in **Ce**rvical **De**generative Myelopathy), the first regenerative medicine trial for degenerative cervical myelopathy (DCM), which aims to test disease-modulating activity of the PDE3/4 inhibitor Ibudilast as an adjuvant to surgical decompression.

DCM is a common and progressive condition with devastating impact on quality of life

DCM is the most common cause of spinal cord impairment world-wide,[1,2] with some estimates of the prevalence as high as 2% of adults.[2,3,4] It arises when arthritic or developmental changes in the cervical spine compress the spinal cord, causing a progressive slow-motion spinal cord injury.[5] As a degenerative pathology the incidence is expected to rise in an ageing population.[6,7]

The consequences of DCM are numerous, varied, and often progressive. Symptoms include pain, loss of dexterity, imbalance and frequent falls, incontinence and in extreme circumstances paralysis.[1,8,9,10,11] A recent comparative study found sufferers have amongst the worst quality of life scores of all chronic disease,[12,13] and this is likely to also negatively impact on their supporters.[14] The cost of DCM to society has not been measured yet, but it is likely to be significant. Consequently, improving recovery after surgery is a significant unmet need and there is strong evidence that surgical treatment for DCM is cost-effective.[15]

Surgery is the only evidence-based treatment for DCM

At present, the only effective treatment for DCM is surgery. Whilst surgery can stop disease progression, the existing damage does not fully recover[16] and people with DCM retain life-

For peer review only - http://bmjopen.bmj.com/site/about/guidelines.xhtml

long disabilities with severe impact on quality of life.[12,13] Many remain unable to return to full time work and reliant on others for day-to-day activities.[17] Given the severe long-term consequences of DCM, treatment alternatives that promote recovery are desperately needed.

Phosphodiesterase 3 inhibition promotes functional recovery in preclinical DCM

The Mitogen-Activated Protein Kinases (MAPK) play a vital role in intracellular signalling.[18] In response to extracellular stimuli, such as neurotransmitters, inflammatory factors or stress conditions, this family of interconnected serine/threonine kinases coordinates a diverse range of intracellular processes, including cell differentiation, proliferation and apoptosis, inflammation and stress responses.[19] This signalling pathway and its modulation have therefore been linked to many diseases including cancer, asthma, stroke, multiple sclerosis and Alzheimer's dementia. More recently, preclinical studies, including our own, have demonstrated that its modulation via inhibition of a class of enzymes called phosphodiesterases (PDE), can improve functional recovery and reduce the perception of pain following damage to the central nervous system.[20,21,22]

PDEs hydrolyse the intracellular messenger cyclic AMP (cAMP).[20,23] This results in modulation of MAPK signalling.[24,25] Inhibition of PDE3 is particularly attractive in DCM as treatment with the selective PDE3 inhibitor cilostozol resulted in improved functional recovery in a rat model of DCM,[26] likely by improving latent ischemia.

Improvements following surgery are associated with axon sprouting, re-myelination, and immunomodulation

In DCM, tethering and compression of the spinal cord initiates a cascade of secondary injury events, including ischemia, inflammation and apoptosis that ultimately cause increased neurological deficits.[5,27,28] The partial reversal of symptoms after surgery highlights an inherent, albeit attenuated, regenerative capacity of the spinal cord.[16,29] This is echoed by post-mortem studies and our preclinical data, which indicate that neurological recovery following decompression is associated with axonal plasticity, re-myelination, and modulation of the immune response.[29,30,31] Enhancing axonal plasticity and re-myelination is therefore key to improving outcomes after DCM.[32]

Phosphodiesterase 4 inhibition can promote functional recovery and modulate pain in preclinical models

For peer review only - http://bmjopen.bmj.com/site/about/guidelines.xhtml

PDE4 is another isoform of phosphodiesterase inhibitors, which has demonstrated preclinical benefits on axon outgrowth[20] and remyelination.[22] The best characterised application of PDE4 inhibitors involves preclinical models of traumatic spinal cord injury using a drug called rolipram.[20,21] Unanimously, these have demonstrated that modulation of the PDE4 cascade is able to benefit recovery. In addition, our own work demonstrated that inhibition of PDE4 is able to stimulate the regenerative response of a CNS stem cell population termed oligodendrocyte progenitor cells and engage in re-myelination,[22] a process that has been observed in post-mortem spinal cords affected by DCM.[30]

PDE4 inhibition also has a role in modulating the perception of pain. Central to the development and maintenance of chronic pain syndromes is glial activation within the central nervous system, which enhances pain sensitivity via neuronal-glial interactions.[33] Modulation of MAPK via PDE4 inhibition has demonstrated a reduction in pain in several preclinical models.[34,35,36,37] Bao *et al*. (2011) found that PDE4 inhibition improved not just motor recovery but also resulted in a reduction in neuropathic pain in a rat model of spinal cord injury.[38] PDE4 inhibition also has an anti-inflammatory effect, increasing cAMP production in leukocytes and therefore reducing the release of tumour necrosis factor-alpha, a potent inflammatory mediator and peripheral pain stimulus.[39]

Ibudilast is a potent PDE4 inhibitor with an excellent human safety profile

The majority of preclinical studies described have used rolipram for PDE4 inhibition. Whilst rolipram is a potent and selective PDE4 inhibitor, experience from translational trials, most recently in multiple sclerosis (MS),[40] have demonstrated poor tolerability in humans due to significant nausea and vomiting. The MS trial had to be terminated due to a lack of efficacy and poor tolerability. Additionally, preclinical evidence has demonstrated a narrow therapeutic window, with potentially adverse neurological sequalae if missed.

An alternative is Ibudilast (MN-166).[23] Ibudilast is a potent PDE4 inhibitor, with additional PDE3 and 5 receptor activity. Modulation of PDE3 is also attractive in DCM as it led to improved function in a preclinical model of DCM.[26] Another attractive feature of Ibudilast is that it has been in clinical use for over 20 years for the treatment of asthma and post-stroke dizziness, without tolerability issues.[41]

Ibudilast is currently under investigation for a number of other neurological conditions,

For peer review only - http://bmjopen.bmj.com/site/about/guidelines.xhtml

including alcohol [NCT03489850] and methamphetamine [NCT01860807] addiction, glioblastoma [03782415], amyotrophic lateral sclerosis (ALS)[42] and multiple sclerosis[43] in a series of double blind, placebo randomised controlled trials.

For ALS, a single Phase I/II trial has been completed. Two ALS cohorts of early stage disease and advanced stage disease requiring ventilation, were randomised 1:1 to receive Ibudilast or placebo. Overall, the primary endpoint of safety and tolerability was met. In the early stage disease takers, Ibudilast was associated with a significant increase in survival, and delayed requirement for ventilation.[44] Treatment effects were linked to per-protocol adherence to therapy.[45] A Phase III trial is now planned.

For MS, two phase II trials have been completed. The first one evaluated relapsing remitting MS; whilst it did not prevent the development of new brain lesions, it slowed the progression of brain atrophy in a dose dependent fashion. The second one, a follow-up study in progressive MS, found that Ibudilast significantly slowed the progression of brain atrophy.[46]

Of note, typical daily dosing in these trials ranged from 60-100mg, which is greater than the currently licensed dosing of 10-20mg per day for routine clinical practice. Whilst trials confirmed overall tolerability and safety for use of Ibudilast in these doses in humans, findings do indicate a dose dependent relationship for gastro-intestinal side effects, such as nausea, and headaches and, in a minority of cases, this led to discontinuation of therapy by participants.

RECEDE-Myelopathy (Regeneration in Cervical Degenerative Myelopathy)

RECEDE-Myelopathy is a multi-centre, double-blind, randomised, placebo-controlled trial assessing the efficacy of Ibudilast as an adjuvant treatment to decompressive surgery for degenerative cervical myelopathy. The specific mechanism of action of Ibudilast is highly suited to address both functional outcome and neuropathic pain in DCM. Therefore, prompted by the direct involvement of people with DCM in designing the study, RECEDE Myelopathy has an infrequently used study design of two co-primary endpoints. It is designed and powered to detect response of patients to Ibudilast with regards to function or pain, independently, as well as a response to both endpoints. We hypothesise that Ibudilast promotes functional outcome and reduces pain in surgically treated DCM.

For peer review only - http://bmjopen.bmj.com/site/about/guidelines.xhtml

Methods

Study design and objectives

RECEDE-Myelopathy (**Re**generation in **Ce**rvical **De**generative **Myelopathy)** is a multi-centre, double-blind, randomised, placebo-controlled trial assessing the efficacy of Ibudilast as an adjuvant treatment to decompressive surgery for degenerative cervical myelopathy. Participants will be randomized to receive either 60-100mg Ibudilast (interventional arm) or placebo (control arm) starting within 10 weeks prior to surgery and continuing for 24 weeks after surgery for a maximum of 34 weeks of treatment. Pre-operative treatment may leverage the effects of inhibition of PDE3, whilst post-operative treatment aims at regeneration-inducing effects outlined above. The primary objective will be to compare improvement in pain or physical function at 6 months after surgery between the two arms of the trial. We hypothesize that adjuvant therapy with Ibudilast leads to a meaningful and additional improvement in either pain or function, as compared to standard routine care (decompressive surgery). Planned start date for study recruitment is September 2021, with planned end being September 2025.

***Patient and Public involvement (PPI)* - aligning research with patient priorities**

The involvement of public and patients representatives in research is recognised to be of key importance to ensure it delivers meaningful, practice-changing information. [47,48, 49, 50] As with many fields, this has been a problem for DCM. [51, 52, 53] To address this issue, we founded Myelopathy.org, the first and so far only charity for people with DCM. Whilst in its infancy, the platform has become an international focus for people with DCM, hosting a peer-to-peer support community (Myelopathy Support) of over 2000 users.[54] This has enabled larger scale insights into the perspective of individuals with DCM[17, 55, 56,] and ultimately led to RECODE-

For peer review only - http://bmjopen.bmj.com/site/about/guidelines.xhtml

DCM, a James Lind Alliance-led initiative to identify and define the research priorities for DCM.[51] (https://aospine.aofoundation.org/research/recode-dcm)

Definition of recovery priorities for people with DCM

AO Spine Research Objectives and Common Data Elements for Degenerative Cervical Myelopathy (AO Spine RECODE-DCM) is an international initiative to create a 'Research Toolkit' to help improve and accelerate knowledge gained in DCM and help to improve outcomes. As part of RECODE DCM, a focus group of people with DCM was created with the objective to develop recovery domains. These were subsequently prioritised via an international, online survey (n = 485).[10] In contrast to the research focus to date, [52,, 57] pain emerged as the number one recovery priority, closely followed by hand, and walking function. Consequently, the development of adjuvant treatments for DCM should be most usefully focused on reducing pain and improving limb function.

Patient screening and eligibility

A summary of the study flow diagram, including full inclusion and exclusion criteria, is presented in Figure 1. In summary, adults (age 18-80) with a diagnosis of DCM (participants must have at least one MRI indicator, clinical symptom, and neurological sign from Table 1 to be eligible for inclusion) and a disease severity of modified Japanese Orthopaedic Association scale (mJOA) 8-14 inclusive, scheduled for their first decompressive surgery, will be approached to consider participation in RECEDE-Myelopathy.

Table 1: Trial criteria for diagnosis of DCM. Participants must have at least one MRI indicator, clinical Symptom, and neurological sign to be eligible for inclusion.

**MRI Indicators**	**Clinical Symptoms**	**Neurological Signs**
Effacement of CSF and deformation of cord	Numb hands	Pyramidal weakness
T1 signal change	Clumsy hands	Hyperreflexia
T2 Signal change	Bilateral arm paraesthesia	Positive Hoffman sign
Segmentation of T2 signal change	Gait impairment	Upgoing plantar response
Reduction in transverse area of cord	Lhermitte's phenomenon	Atrophy of intrinsic hand muscles
	Weakness	Spasticity/clonus
		Broad based, unstable gait

For peer review only

Eligibility will be further assessed against exclusion criteria, largely dictated by safety requirements for use of Ibudilast, and in precluding masking of treatment effects. This includes, concomitant lumbar canal stenosis or other neurological condition, presentation with symptoms due to trauma (e.g., central cord syndrome), a history of allergy to Ibudilast, any of its formulation or that of the placebo, pregnancy, unwillingness to use reliable contraception, active malignancy, liver impairment or thrombocytopaenia. The latter will be assessed via serum biochemistry and haematological assessment. A full list of exclusion criteria can be find in table 2.

For peer review only

Table 2. Exclusion Criteria

1	Previous surgery for degenerative cervical myelopathy
2	Degenerative cervical myelopathy symptoms due to cervical trauma, determined at the discretion of the investigator
3	Hypersensitivity to Ibudilast or any of the formulation components
4	Evidence of acute hepatitis, clinically significant chronic hepatitis, or evidence of clinically significant impaired hepatic function through clinical and laboratory evaluation (including ALP >1.5x ULN; ALT or AST >2x ULN; GGT >3x ULN)
5	Evidence of thrombocytopenia at screening through laboratory evaluation including platelet count <5000
6	Active malignancy defined as a history of invasive malignancy, except if the patient has received treatment and displayed no clinical signs and symptoms for ≥5 years
7	Recent history (≤3 years) of chemical substance dependency or significant psychosocial disturbance that may impact the outcome or trial participation
8	Female patients with childbearing potential who are unwilling or unable to use reliable methods of contraception
9	Female patients who are pregnant, lactating or planning pregnancy during the course of the trial
10	Inability to comply with trial procedures or follow-up schedule including IMP regime
11	Unable to take gelatin-based product
12	Participation in another CTIMP or device trial ≤30 days before the time of recruitment
13	Functional disability from a concomitant neurological disease that would mask the symptoms of degenerative cervical myelopathy, determined at the discretion of the investigator. Including but not limited to stroke with a residual disability, cerebellar ataxia, Parkinson's disease, symptomatic lumbar stenosis, and multiple sclerosis.
14	Resting pulse < 50 bpm, sinoatrial or atrioventricular block, uncontrolled hypertension, or corrected QT interval (QTcF) >450 ms

15	History of stomach or intestinal surgery or any other condition that could interfere with, or is judged by the investigator to interfere, with absorption, distribution, metabolism, or excretion of IMP
16	Unable to converse, read, or write English

Enrolment and randomisation

Those patients who satisfy the screening criteria and agree to study participation are enrolled and randomized at 1:1 to one of the two treatment arms. A web-based randomisation system (Sealed envelope) performing stratified blocked randomisation will be used stratifying by baseline mJOA (<12 vs. >=12), age (<60 years vs. >=60 years) and time to onset of the disease (>6 months vs. <=6 months); random block size will be used. Throughout randomisation and follow-up, the subjects, physicians, and data collectors remain blinded to group allocation.

Treatment description and dosage modification

The investigational medicinal product (IMP) is a 24-34 week course of Ibudilast or matched placebo in an escalating dosage regimen up to a maximum of 100mg daily if tolerated. The escalating dosage regimen is to minimise gastrointestinal side effects. Ibudilast is available in 10mg capsules, and therefore the IMP will be provided as such. The placebo is identical in shape, size, and color to the Ibudilast capsule, and participants will be provided with the same instructions.

Participants will start treatment within 10 weeks prior to surgical decompression and will continue taking drug for up to 24 weeks post-surgery. The excretion half-life of Ibudilast is approximately 20 hours. The IMP will be taken in divided doses, twice daily, morning and evening, for a maximum of 34 weeks. Because this is the first surgical trial with Ibudilast, and to mitigate any potential interference on the coagulation system, treatment will be halted 5 days prior to surgery and resumed at the previous maximum dose right after operation.

Ibudilast is associated with gastro-intestinal side effects, such as nausea and dyspepsia. Alongside dose escalation, participants will be instructed to take trial medication with food or within an hour of eating to improve gastrointestinal tolerability. In the event of minor gastrointestinal complaints, participants will be offered symptomatic treatment in the first instance, in conjugation with ongoing IMP therapy. If this is unsuccessful, or not agreeable to participants, the trial therapy will be decreased in decrements of 20mg every 5 days, until a

For peer review only - http://bmjopen.bmj.com/site/about/guidelines.xhtml

tolerable dosing level is achieved, or the drug is stopped. If a participant cannot tolerate a minimum daily dosage of 60mg despite additional supportive measures, treatment within the trial will be stopped.

Surgery

There are a number of different approaches used to decompress the spinal cord in DCM. No surgical approach has been shown to be superior, and the consensus is that the approach needs to be tailored to the specific anatomy. The surgical care of participants will therefore be at the discretion of the treating clinician and not protocolised.

Outcome measures and follow up

Two patient-informed co-primary endpoints: pain and function

Inhibiting phosphodiesterases 3 and 4 with Ibudilast has the potential to benefit both pain and functional recovery by promoting repair mechanisms in the spinal cord as well as exerting neuroprotective effects. This provides a unique opportunity to address the most important recovery priorities identified by individuals with myelopathy. Therefore, RECEDE-Myelopathy has two outcome targets: pain and physical function.[10] These co-primary endpoints will be assessed at 6 months after surgery, a time point when the majority of recovery will have been achieved. [57]

The study is thus powered to detect meaningful changes with regards to the co-primary endpoints independently from each other, i.e., it is designed to establish whether Ibudilast has beneficial effects on function or pain alone or whether it beneficially modulates both end points

Co-primary endpoint 1. The international standard, and most validated measure for assessment of function in DCM, is the mJOA scale.[16, 58,59] The mJOA is a composite score of upper and lower limb muscular function, upper limb sensory function and bladder function.

Co-primary endpoint 2. Pain has been identified as the recovery priority of DCM patients. The most common form is neck pain,[9] with a neuropathic component that is responsive to neuroprotective treatments. [60, 61]

For peer review only - http://bmjopen.bmj.com/site/about/guidelines.xhtml

Whilst numerous tools have been developed for the measurement of pain,[62] the Initiative on Methods, Measurements and Pain Assessment in Clinical Trials (IMMPACT) agree that pain intensity scales provide the most relevant outcome measure for demonstrating efficacy. In DCM the visual analogue scale (VAS) is the most popular example of this.[63] Although not exclusively validated for DCM, the psychometric properties of VAS neck and VAS arm pain have been evaluated in degenerative disease of the cervical spine,[64,65] with VAS neck pain having better repeatability.

This design will address the most important priorities of people with DCM.[10] It leverages the mechanism of action of Ibudilast to maximise the chances of demonstrating the benefit of the studied intervention. It will increase the knowledge that can be gained through the study and demonstrate whether the proposed mechanisms of neuroprotection and regeneration can be applied to promote function and/or reduce pain. Finally, the dual end-point design will make the study more efficient than conducting two independent trials. The chosen two endpoint design will hence increase the value of the study.

Secondary and exploratory endpoints

Clinical assessments will additionally be undertaken pre-operatively, post-operatively and 3, 6 and 12 months after surgery. The disability reported in the context of DCM is wide ranging. In the absence of a consensus dataset,[51.] an issue that we are currently attending to as part of RECODE-DCM, a variety of clinician administered and patient reported outcome measures will be used to provide a comprehensive assessment. A full list of assessments and their time-points is presented in table 3.

Table 3: Schedule of Assessments

Assessments	Screening visit and initial assessments	Randomisation	Start of IMP (within 2 -3 month prior to Surgery)	Pre-Operative assessments (within 21 days prior to surgery)	Surgery	Post-operatively/ Discharge (within 14 days post-surgery)	3- months Post Operatively (±21 days)	6-months Post Operatively (±21 days)	12-months Post Operatively (±21 days)
Informed consent	X								
Eligibility Assessment	X								
Demographics	X								
Medical history & DCM characteristics	X								

For peer review only - http://bmjopen.bmj.com/site/about/guidelines.xhtml

Concomitant medication	X			X			X	X	X
Blood Tests (FBC, LFT, E/U/C, TFTs)	X			X			X	X	X
ECG	X								
Urine analysis	X								
Pregnancy test	X								
Randomisation		X							
Neurological examination	X			X		X	X	X	X
mJOA	X			X			X	X	X
30m Walk test	X			X			X	X	X
GRASSP-Cervical Myelopathy	O			O			O	O	O
SCIMv3	O			O				O	
WHO performance status				X					
Neck Disability Index	O			O		O	O	O	O
VAS Pain	X			X		X	X	X	X
SF-36	X			X			X	X	X
EQ5D / Health Resource Usage	X			X			X	X	X
Quick-DASH	O			O			O	O	O
Carer QoL (sub-study)	X			X			X	X	X
Review of AEs			X	X		X	X	X	X
Dosing Diary	X								
Dispensing of IMP			X			X	X		
Serum sample for PK studies	X			X	X		X	X	X
Compliance Assessment				X		X	X	X	
IMP review				X		X	X	X	
Respiratory Physiology & muscle function				X				X	
MRI				X				X	
Gait Lab (sub-study)				X			O	X	
Surgery details					X				
Surgery complications						X	X	X	X
Hospital discharge						X			
CSF sample					O				

For peer review only

Not all assessments will be conducted at every time point, or be mandated, to reduce participant and investigator burden. Assessment is also extended to carers of participants. Building on our preliminary finding of reduced quality of life amongst DCM carers,[14] the Care Quality of Life instrument (CarerQol) will be used to evaluate this.[65.]

Adaptive sample size design

The minimum clinically important difference (MCID) for the mJOA is estimated to be between 1 and 2 points.[66] Although not exclusively validated for DCM, the MCID for VAS neck and VAS arm pain has been calculated for degenerative disease of the cervical spine with values ranging from 8-26mm.[64, 65] Both VAS pain and mJOA improve more than the MCID with surgery alone,[57] and the amount of change is linked to the pre-operative baseline.[66] Consequently, in consensus with patients we have determined the MCID of the VAS pain score as being 1cm and for the mJOA 1 point. This has been modelled to ensure statistical power across all baseline scenarios.

On this basis, a total sample size of 362 participants under equal randomisation will provide 85% power to detect a difference of 1 between treatment arms on the mJOA scale (assuming a standard deviation (SD) of 2.89), using a two-sided t-test at a 2.5% significance level to adjust for multiple comparisons.[67] The trial is also powered to detect a similar difference on the VAS neck pain scale (assuming a difference of 1 and a SD of 2.88).

For peer review only - http://bmjopen.bmj.com/site/about/guidelines.xhtml

A blinded interim analysis will be conducted to refine the power calculation. The aim will be to reassess the sample size in time to allow any potential extension and increase in sample size to be put into effect. Reduction in sample size will not be permitted. Any sample size increase will be based on checking the assumption regarding the SD, and will not estimate any treatment effect, hence no subsequent adjustment to future analyses is needed.
Under such a framework, the theoretical optimal time to schedule such an interim analysis would be just as the last patient is recruited under the original sample size (n=362) following which a decision could be taken to either halt or extend recruitment. However, for reasons of practicality a window for the interim analysis will be up to a period of 4 months before reaching the total sample size.

The SD and correlation of both endpoints will be reassessed using data pooled across the arms. The three possible statistically significant conclusions of the formal hypothesis testing (VAS; mJOA; both) will be provided with revised target sample sizes needed to achieve 85% power under the same MCID values, but with revised estimates for the SD values and correlation. A recommended revised sample size will be the smallest of the three new target sample sizes or the original sample size if this is larger; hence the recommended sample size will never be a reduction from the original.

The next step of the interim analysis will be to calculate the conditional power of the three possible positive outcomes based on, the estimated unblinded treatment effects from the current data, plus, the distribution of future data from the revised sample size under the corresponding combinations of true treatment effects (MCID or zero), and SD and correlation estimates from the first step. If all three conditional power values are less than 30% then the recommendation would be to halt the study.

Trial monitoring

All data collected during the trial will be recorded into a Case Report Form (CRF), which will be labelled using a participant's unique trial ID and date of birth. CRFs will be completed by the local research team and copies will be sent to trial coordination centre, where it will be entered into a central digital database. Safety assessments will be conducted by local investigators and reported and handled according to a predefined trial protocol. This includes a mechanism to capture surgical complications.[68] The Trial Steering Committee

For peer review only - http://bmjopen.bmj.com/site/about/guidelines.xhtml

(TSC) will provide overall supervision with respect to the conduct of the trial. The TSC will consist of an independent Chairperson (Prof Michael Fehlings), a PPI representative (Mr Iwan Sadler), independent clinical and science experts (Prof Marios Papadopoulos and Dr Mark Bacon), clinical pharmacology and neurosurgery experts (Prof Ian Wilkinson and Prof Peter Hutchinson), the Chief Investigator and members of the Trial Management Group (e.g., trial statistician, trial manager). The ethical and safety aspects of the trial will be overseen by an independent Data Monitoring Committee (DMC) who will meet once a year and their meetings will be timed so that reports can be fed into the TSC meetings. Safety assessment will be performed for every participant since consent and until end of their participation in the trial. To date, there are no known expected serious adverse reactions (SAR) for Ibudilast, and thus any reported SAR will be considered a suspected unexpected serious adverse reaction (SUSAR). Furthermore, surgical complications will be followed up as events of special interest to be reviewed by the DMC.

Statistical methods

The primary endpoint and key secondary endpoints are all measured on a continuous scale. A comparison of mean values between treatment arms, adjusting for baseline covariates, will be provided using linear regression. Estimates, standard errors, 95% confidence intervals and p-values will be provided.

For formal hypothesis testing, a closed testing approach will be used to deal with multiple endpoints.[69] Initially either of the co-primary endpoints (mJOA or VAS neck pain) may test a null hypothesis of zero mean difference at a 2-sided 2.5% significance level, [70] with the remaining primary endpoint tested at 5% significance level. This will enable us to determine whether the study drug is effective on pain or function independently.

Subsequently a gate-keeping approach will be used where an endpoint below the primary endpoint in the pre-specified ordering is only tested if all the preceding endpoints reject the null hypothesis, using the nominal p-value. If an endpoint does not reject the null, then all endpoints below it have the same conclusion-not rejecting the null-regardless of their nominal p-value. The ordering is, after primary endpoints, SF-36 PCS and then SF-36 MCS.

For peer review only - http://bmjopen.bmj.com/site/about/guidelines.xhtml

Secondary endpoints will be compared between treatment arms using approach regression techniques: linear regression for continuous endpoints, logistic regression for binary endpoints, and Cox regression for time-to-event.

The following baseline covariates, in addition to the baseline value of the endpoint, will be used to adjust all comparisons

- Time to onset
- Smoking status (yes/no)
- Age
- Psychiatric comorbidities (yes/no)
- Impaired gait (yes/no)

A detailed statistical analysis plan will be produced before the final database lock.

Discussion

This is the first regenerative medicine trial for DCM. It is also the first trial to target all the recovery priorities for people with DCM, namely pain and upper and lower limb function as primary endpoints.[10] This is significant, as in the recent evaluation of Riluzole as a perioperative neuroprotective therapy in DCM, whilst the primary end-point (1-point change in mJOA) was not met, VAS neck pain, a secondary end-point, improved significantly. [58] However, as a secondary endpoint the causal link can only be tentative.

RECEDE-Myelopathy addresses 5 of the 10 top priorities identified by RECODE-DCM

Priority 1 - Raising awareness[1,71]:

- RECEDE-Myelopathy is the first regenerative medicine trial for DCM. It is the second powered DCM CTIMP world-wide. We will seek to leverage this fact to attract attention to DCM by optimising communication before, during and after the trial, aiming at maximising our audience, to include patient organisations, a wide range of health care providers and the scientific community. We also aim to break into non-specialist mainstream media.

Priority 2 – Assessment and monitoring:

- RECEDE-Myelopathy will help to standardise assessment and monitoring across study centres, and thus promote the implementation of the recent international

For peer review only - http://bmjopen.bmj.com/site/about/guidelines.xhtml

guidelines.[16] Additionally, a number of new secondary endpoints are included for the first time in a clinical trial of DCM, including gait[72] and respiratory physiology.[73]

Priority 5 – Developing a better understanding of the pathophysiology of DCM[74]

- RECEDE-Myelopathy tests the hypothesis that MAPK signalling mediated by PDE3/4 can promote recovery after DCM. It will serve as a platform for sub-studies, including imaging studies, and molecular biology studies on blood draws and CSF.

Priority 6 – Rehabilitation:

- There are no evidence-based measures to promote rehabilitation in DCM.[75] RECEDE-Myelopathy will investigate a drug that has the possibility of improving functional outcomes in DCM.

Priority 7 – Novel therapies:

- At present, surgery is the only possible treatment for DCM. If successful, RECEDE-Myelopathy will pave the way for the first evidence-based non-surgical adjuvant treatment.

Neuropathic origins of neck pain in DCM

Pain has been identified as the recovery priority of people with DCM.[10] Where assessed, previous trials have focused on neck (or axial) pain and arm pain.[61,58,76] Our findings in a survey of 230 patients, found neck pain was the most commonly reported first symptom of DCM (13%), and with respect to pain, twice as common as limb pain (7%). Moreover, overall neck pain was experienced more often (80%) than arm pain (70%) (56). In addition, individuals can be affected by atypical pain syndromes such as headache.[8,77,11]

Counter to the prevalent belief that neck pain is mainly caused by arthritic changes to the spine, an emerging literature points to a neuropathic origin. First, arthritic changes are omnipresent with progressive age, causing increasing levels of cord compression.[3,9] In many instances this does not lead to neck pain, even in the context of DCM.

A neuropathic component of chronic neck pain has long been postulated. For example, a psychophysical study measuring responses to electro-cutaneous stimulation in subjects with chronic neck pain found evidence of secondary hyperalgesia which, in turn, implies central sensitisation of nociceptive pathways.[78] The results were compatible with studies which identify potential anatomical origins of chronic neck pain but provide evidence that central sensitisation may be the relevant mechanism of pain production.

For peer review only - http://bmjopen.bmj.com/site/about/guidelines.xhtml

A single centre study investigated the relation between pain provoking cervical segments identified by diagnostic dorsal root blockades and elevation of quantitative sensory testing of the cervical dermatomes using Semmes-Weinstein monofilaments in patients suffering from neck pain but not radiculopathy. This revealed a systematic elevation of detection thresholds, an adaptation in contrast with, but not contradictory to, central sensitization of high threshold neurons in chronic pain.[79]

More recently, a study of non-specific neck pain investigating neuropathic components, and in particular neck pain-associated functional abnormalities related to sensory and sympathetic innervation demonstrated signs of functional impairment of innervation. These were reflected in changes in tactile sensitivity and vasoactive sympathetic function and may be based on both central and peripheral mechanisms.[80] Of note, osteoarthritic pain does not change sensory or pain thresholds in individuals with neck pain.[81]

Another striking piece of evidence in support of a neuropathic component underlying neck pain are the findings of the CSM-Protect trial, the first adequately powered double blind randomised controlled drug trial for DCM.[58] Riluzole is an approved neuroprotective drug in clinical use for amyotrophic lateral sclerosis. It has been linked to reducing glutamatergic excitotoxicity in neurons via a number of mechanisms.[82] Although Riluzole treatment did not alter functional outcome in DCM, significant improvements in neck pain were detected.[58]

A neuropathic pain component in DCM is further supported by recent preclinical findings which echoed the findings of the clinical trial.[59] Finally, it must not be overlooked that DCM is a form of spinal cord injury. The importance of neuropathic pain in SCI is well established.[83]

Outcome assessment in DCM is a challenge for translational research and will be further evaluated.

As outlined, the selection of VAS neck pain, and the mJOA is based on the current best available assessments. Whilst the mJOA is a robust, and fully validated measure, this scale does not capture pain and has a reduced sensitivity to change in milder disease.[59] Presently, there is no combined assessment tool of function and pain validated for DCM,[84] with pain typically captured using visual analogue scales.[61,,77] RECODE-DCM, a parallel international

For peer review only

consensus initiative is underway to determine the most suitable outcome measurements for DCM. [51]

This has led to two important considerations in the design of this trial: the selection of the inclusion criteria and of the trial endpoints.

The eligibility criteria were designed to ensure the most cost-efficient design and likelihood of success.[85] The surgical treatment of mild DCM is controversial,[16] and therefore risks underrepresentation in this trial if included. Additionally, surgical treatment alone is likely to return a maximum mJOA score in mild disease.[85] Alongside the recognised plateau effect of higher mJOA scores, this therefore risks masking a treatment effect. To prevent these effects, only moderate/severe scores in the mJOA are included in the trial. Similarly, this is the concern for neurological comorbidities or previously treated myelopathy. The mJOA is a measure of functional disability and therefore neurological comorbidities may instead be measured.[84] This is why other neurological comorbidities that could mask the symptoms of DCM are excluded from the trial. Based on experience from traumatic spinal cord injury,[86] it is anticipated that the biological recovery capacity is altered in patients with previously treated myelopathy. Additionally, this subgroup has received relatively little research,[76] and the data informing the surgical response and MCID is based on series which excluded repeat surgery.[57,87] Previously treated myelopathy is under-researched, but the pre-clinical regenerative capacity is anticipated to be different, as are the surgical response and appropriate MCIDs. Patients who underwent surgery for DCM in the past are thus excluded.

In addition, a broad range of secondary endpoints have been included. These assessments have been selected to capture the far-ranging disability experienced by people with DCM. It includes the evaluation of promising objective, quantitative measures, such as microstructural MRI,[88] respiratory physiology,[73,89] GRASSP-Myelopathy (adapted from GRASSP[90]) and gait-laboratory analysis.[91,92] It also includes an assessment of carer quality of life for the first time in a DCM trial.[14] It is recognised that these additional assessments increase the time requirements on participants and investigators, and therefore only a fraction are defined as per protocol. The identification and establishment of improved assessment measures would be of value to future trials and clinical practice.

For peer review only - http://bmjopen.bmj.com/site/about/guidelines.xhtml

Summary

RECEDE-Myelopathy will evaluate the efficacy of Ibudilast, as adjuvant treatment, to improve recovery after surgical decompression in DCM. It is the first regenerative medicine trial in DCM, and the first DCM trial to directly target all the recovery priorities identified by sufferers.

Ethical approval and dissemination.

The RECEDE-Myelopathy trial protocol version 2, 11 March 2020, informed consent forms and all other relevant trial documents have been approved by Central London Research and Ethics Committee (REC), reference 20/LO/0185. HRA approval from HRACW was received on 01/07/2020.. Annual reports will be submitted to the REC in accordance with local national requirements. Trial will be performed following GCP from the ICH guidelines and the letter of the Declaration of Helsinki, as well as any other local regulatory requirements and laws.

All enrolled subjects will have the capacity to consent for the trial and can withdraw from the study at any point. Consent will be obtained by the research team and confirmation of consent to continue partaking in the study will be done on every trial visit.

Dissemination of outcomes and findings from the study with patient involvement

We intend to involve Patients with DCM in the dissemination of research output, both in the production of scientific and lay material, and its communication. Finally, we are currently evaluating the use of PPI representatives to communicate findings to professional audiences. The results of the study will also be presented at international scientific conferences and in peer-reviewed journals regardless of the trial outcome.

Ownership of the data arising from this trial resides with the trial team. On completion of the trial the data will be analysed and tabulated and a Final Trial Report prepared.

We intend to disseminate the findings via peer-reviewed journals and presentations at national and international meetings. In addition to meetings orientated around neurosurgery, we will target conferences organised for the different health professionals who care for patients with DCM, including Neurology, Primary Care, Geriatrics and Rehabilitation medicine. We will publish the results of the trial on the EudraCT website.

Research findings will be disseminated to relevant service user groups and charities (including Myelopathy.org) through newsletters, website posts and public presentations. The dedicated trial website will also include dedicated pages for members of the public. We will

For peer review only

present the trial in open days organised by hospitals participating in the trial where members of the public are invited to find out about on-going research.

Participants will be able to view global trial results on the trial website.

The trial partners, funders and sponsor will be acknowledged in the publication. Any scientific paper, presentation or communication concerning the trial shall be submitted to each relevant party following their guidelines.

We do not intend to distribute deidentified patient data at this point of time.

For peer review only

Footnotes:

Contributors:
The idea and project were conceived by MRK and BD. The initial draft of the manuscript was created by BD, OM, and circulated amongst other authors for critical revisions. Critical revision and protocol manuscript amendments were collated and executed by SY. BD,OM,SY,DA,SB,MN,PK,LW,JB,SC,SL,MB,MCP,MS,IS,LS,SKR,AC,RT,MW,DC,IW,MGF,PJ,MRK have approved the final version of the manuscript and have been involved in the critical revision of the manuscript. The Chief investigator for RECEDE is MRK and co-investigator is BD. As guarantors are acting MRK, BD and SY.

Competing Interest:
MRK holds a research grant from clinical scientist award and has support for the study from Medicinova. MCP holds research grant award with the NIHR. BD holds research grants with NIHR HTA POLYFIX-DCM, Evelyn Trust (DCM – COINs) and award from National Lottary UK for developing a peer-to-peer support community for Degenerative Cervical myelopathy.BD is also a founder of MoveMed LTD. PJAH holds NIHR research grants.

Funding
Clinician Scientist Award, CS-2015-15-023, supported by the National Institute for Health Research

Ethics Approval:
IRAS No:213009 Ethics Committee : London Central Research Ethics Committee.

For peer review only - http://bmjopen.bmj.com/site/about/guidelines.xhtml

References

1. Davies BM, Mowforth OD, Smith EK, Kotter MR. Degenerative cervical myelopathy. *BMJ*. 2018;360:k186. doi:10.1136/bmj.k186

2. Kadanka Z, Adamova B, Kerkovsky M, et al. Predictors of symptomatic myelopathy in degenerative cervical spinal cord compression. *Brain Behav*. 2017;7(9). doi:10.1002/brb3.797

3. Kovalova I, Kerkovsky M, Kadanka Z, et al. Prevalence and Imaging Characteristics of Nonmyelopathic and Myelopathic Spondylotic Cervical Cord Compression. *Spine*. 2016;41(24):1908-1916. doi:10.1097/BRS.0000000000001842

4. Smith SS, Stewart ME, Davies BM, Kotter MRN. The Prevalence of Asymptomatic and Symptomatic Spinal Cord Compression on Magnetic Resonance Imaging: A Systematic Review and Meta-analysis. *Global Spine J*. 2021;11(4):597-607. doi:10.1177/2192568220934496

5. Akter F, Kotter M. Pathobiology of Degenerative Cervical Myelopathy. *Neurosurg Clin N Am*. 2018;29(1):13-19. doi:10.1016/j.nec.2017.09.015

6. Nouri A, Tetreault L, Singh A, Karadimas SK, Fehlings MG. Degenerative Cervical Myelopathy: Epidemiology, Genetics, and Pathogenesis. *Spine*. 2015;40(12):E675-693. doi:10.1097/BRS.0000000000000913

7. Grodzinski B, Durham R, Mowforth O, Stubbs D, Kotter MRN, Davies BM. The effect of ageing on presentation, management and outcomes in degenerative cervical myelopathy: a systematic review. *Age Ageing*. Published online November 19, 2020. doi:10.1093/ageing/afaa236

8. Davies BM, Munro CF, Kotter MR. A Novel Insight Into the Challenges of Diagnosing Degenerative Cervical Myelopathy Using Web-Based Symptom Checkers. *J Med Internet Res*. 2019;21(1):e10868. doi:10.2196/10868

9. Boerger T, Alsouhibani A, Mowforth O, et al. Moving Beyond the Neck and Arm: The Pain Experience of People With Degenerative Cervical Myelopathy Who Have Pain. *Global Spine J*. Published online February 25, 2021:2192568220986143. doi:10.1177/2192568220986143

10. Davies B, Mowforth O, Sadler I, et al. Recovery priorities in degenerative cervical myelopathy: a cross-sectional survey of an international, online community of patients. *BMJ Open*. 2019;9(10):e031486. doi:10.1136/bmjopen-2019-031486

11. Mowforth OD, Davies BM, Kotter MR. “I am not delusional!” Sensory dysaesthesia secondary to degenerative cervical myelopathy. *BMJ Case Rep*. 2019;12(4). doi:10.1136/bcr-2018-229033

12. Oh T, Lafage R, Lafage V, et al. Comparing Quality of Life in Cervical Spondylotic Myelopathy with Other Chronic Debilitating Diseases Using the SF-36 Survey. *World Neurosurg*. Published online January 5, 2017. doi:10.1016/j.wneu.2016.12.124

For peer review only

For peer review only - http://bmjopen.bmj.com/site/about/guidelines.xhtml

13. Hirayama Y, Mowforth OD, Davies BM, Kotter MRN. Determinants of quality of life in degenerative cervical myelopathy: a systematic review. *Br J Neurosurg*. Published online November 18, 2021:1-11. doi:10.1080/02688697.2021.1999390

14. Mowforth OD, Davies BM, Kotter MR. Quality of Life Among Informal Caregivers of Patients With Degenerative Cervical Myelopathy: Cross-Sectional Questionnaire Study. *Interact J Med Res*. 2019;8(4):e12381. doi:10.2196/12381

15. Witiw CD, Tetreault LA, Smieliauskas F, Kopjar B, Massicotte EM, Fehlings MG. Surgery for degenerative cervical myelopathy: a patient-centered quality of life and health economic evaluation. *Spine J*. 2017;17(1):15-25. doi:10.1016/j.spinee.2016.10.015

16. Fehlings MG, Tetreault LA, Riew KD, et al. A Clinical Practice Guideline for the Management of Patients With Degenerative Cervical Myelopathy: Recommendations for Patients With Mild, Moderate, and Severe Disease and Nonmyelopathic Patients With Evidence of Cord Compression. *Global Spine Journal*. 2017;7(3_suppl):70S-83S. doi:10.1177/2192568217701914

17. Pope DH, Mowforth OD, Davies BM, Kotter MRN. Diagnostic Delays Lead To Greater Disability In Degenerative Cervical Myelopathy and Represent A Health-Inequality. *Spine*. Published online October 28, 2019. doi:10.1097/BRS.0000000000003305

18. Sun J, Nan G. The Mitogen-Activated Protein Kinase (MAPK) Signaling Pathway as a Discovery Target in Stroke. *J Mol Neurosci*. 2016;59(1):90-98. doi:10.1007/s12031-016-0717-8

19. Lu M, Wang Y, Zhan X. The MAPK Pathway-Based Drug Therapeutic Targets in Pituitary Adenomas. *Front Endocrinol (Lausanne)*. 2019;10:330. doi:10.3389/fendo.2019.00330

20. Nikulina E, Tidwell JL, Dai HN, Bregman BS, Filbin MT. The phosphodiesterase inhibitor rolipram delivered after a spinal cord lesion promotes axonal regeneration and functional recovery. *Proc Natl Acad Sci U S A*. 2004;101(23):8786-8790. doi:10.1073/pnas.0402595101

21. Pearse DD, Pereira FC, Marcillo AE, et al. cAMP and Schwann cells promote axonal growth and functional recovery after spinal cord injury. *Nat Med*. 2004;10(6):610-616. doi:10.1038/nm1056

22. Syed YA, Baer A, Hofer MP, et al. Inhibition of phosphodiesterase-4 promotes oligodendrocyte precursor cell differentiation and enhances CNS remyelination. *EMBO Mol Med*. 2013;5(12):1918-1934. doi:10.1002/emmm.201303123

23. Gibson LCD, Hastings SF, McPhee I, et al. The inhibitory profile of Ibudilast against the human phosphodiesterase enzyme family. *Eur J Pharmacol*. 2006;538(1-3):39-42. doi:10.1016/j.ejphar.2006.02.053

24. Smith FD, Langeberg LK, Cellurale C, et al. AKAP-Lbc enhances cyclic AMP control of the ERK1/2 cascade. *Nat Cell Biol*. 2010;12(12):1242-1249. doi:10.1038/ncb2130

25. Cuadrado A, Nebreda AR. Mechanisms and functions of p38 MAPK signalling. *Biochem J*. 2010;429(3):403-417. doi:10.1042/BJ20100323

For peer review only - http://bmjopen.bmj.com/site/about/guidelines.xhtml

26. Yamamoto S, Kurokawa R, Kim P. Cilostazol, a selective Type III phosphodiesterase inhibitor: prevention of cervical myelopathy in a rat chronic compression model. *J Neurosurg Spine*. 2014;20(1):93-101. doi:10.3171/2013.9.SPINE121136

27. Badhiwala JH, Ahuja CS, Akbar MA, et al. Degenerative cervical myelopathy - update and future directions. *Nat Rev Neurol*. 2020;16(2):108-124. doi:10.1038/s41582-019-0303-0

28. Akter F, Yu X, Qin X, et al. The Pathophysiology of Degenerative Cervical Myelopathy and the Physiology of Recovery Following Decompression. *Front Neurosci*. 2020;14:138. doi:10.3389/fnins.2020.00138

29. Dhillon RS, Parker J, Syed YA, et al. Axonal plasticity underpins the functional recovery following surgical decompression in a rat model of cervical spondylotic myelopathy. *Acta Neuropathol Commun*. 2016;4(1):89. doi:10.1186/s40478-016-0359-7

30. Ito T, Oyanagi K, Takahashi H, Takahashi HE, Ikuta F. Cervical spondylotic myelopathy. Clinicopathologic study on the progression pattern and thin myelinated fibers of the lesions of seven patients examined during complete autopsy. *Spine (Phila Pa 1976)*. 1996;21(7):827-833. doi:10.1097/00007632-199604010-00010

31. Karadimas SK, Moon ES, Yu WR, et al. A novel experimental model of cervical spondylotic myelopathy (CSM) to facilitate translational research. *Neurobiol Dis*. 2013;54:43-58. doi:10.1016/j.nbd.2013.02.013

32. Karadimas SK, Gatzounis G, Fehlings MG. Pathobiology of cervical spondylotic myelopathy. *Eur Spine J*. 2015;24 Suppl 2:132-138. doi:10.1007/s00586-014-3264-4

33. Ji RR, Berta T, Nedergaard M. Glia and pain: is chronic pain a gliopathy? *Pain*. 2013;154 Suppl 1:S10-S28. doi:10.1016/j.pain.2013.06.022

34. Kim HK, Hwang SH, Oh E, Abdi S. Rolipram, a Selective Phosphodiesterase 4 Inhibitor, Ameliorates Mechanical Hyperalgesia in a Rat Model of Chemotherapy-Induced Neuropathic Pain through Inhibition of Inflammatory Cytokines in the Dorsal Root Ganglion. *Front Pharmacol*. 2017;8:885. doi:10.3389/fphar.2017.00885

35. Kim HK, Kwon JY, Yoo C, Abdi S. The Analgesic Effect of Rolipram, a Phosphodiesterase 4 Inhibitor, on Chemotherapy-Induced Neuropathic Pain in Rats. *Anesth Analg*. 2015;121(3):822-828. doi:10.1213/ANE.0000000000000853

36. Kostich W, Hamman BD, Li YW, et al. Inhibition of AAK1 Kinase as a Novel Therapeutic Approach to Treat Neuropathic Pain. *J Pharmacol Exp Ther*. 2016;358(3):371-386. doi:10.1124/jpet.116.235333

37. Espírito-Santo RF, Meira CS, Costa RDS, et al. The anti-inflammatory and immunomodulatory potential of braylin: Pharmacological properties and mechanisms by in silico, in vitro and in vivo approaches. *PLoS One*. 2017;12(6):e0179174. doi:10.1371/journal.pone.0179174

38. Bao F, Fleming JC, Golshani R, et al. A selective phosphodiesterase-4 inhibitor reduces leukocyte infiltration, oxidative processes, and tissue damage after spinal cord injury. *J Neurotrauma*. 2011;28(6):1035-1049. doi:10.1089/neu.2010.1575

For peer review only

39. Francischi JN, Yokoro CM, Poole S, Tafuri WL, Cunha FQ, Teixeira MM. Anti-inflammatory and analgesic effects of the phosphodiesterase 4 inhibitor rolipram in a rat model of arthritis. *Eur J Pharmacol*. 2000;399(2-3):243-249. doi:10.1016/s0014-2999(00)00330-7

40. Bielekova B, Richert N, Howard T, et al. Treatment with the phosphodiesterase type-4 inhibitor rolipram fails to inhibit blood--brain barrier disruption in multiple sclerosis. *Mult Scler*. 2009;15(10):1206-1214. doi:10.1177/1352458509345903

41. Rolan P, Hutchinson M, Johnson K. Ibudilast: a review of its pharmacology, efficacy and safety in respiratory and neurological disease. *Expert Opin Pharmacother*. 2009;10(17):2897-2904. doi:10.1517/14656560903426189

42. Brooks B, Bravver E, Sanjak M, et al. A Single-Center, Randomized, Double-Blind, Placebo-Controlled, Six-Month Clinical Trial Followed by an Open-Label Extension to Evaluate the Safety, Tolerability and Clinical Endpoint Responsiveness of the Phosphodiesterase Type 4 (PDE4) Inhibitor Ibudilast (MN-166) in Subjects with Amyotrophic Lateral Sclerosis (ALS) - STEP-IBUDILAST-ALS-DB-OLE-1 (I8-1B). *Neurology*. 2015;84(14 Supplement). Accessed December 2, 2021. https://n.neurology.org/content/84/14_Supplement/I8-1B

43. Fox RJ, Coffey CS, Cudkowicz ME, et al. Design, rationale, and baseline characteristics of the randomized double-blind phase II clinical trial of ibudilast in progressive multiple sclerosis. *Contemp Clin Trials*. 2016;50:166-177. doi:10.1016/j.cct.2016.08.009

44. Brooks B, Bravver EK, Sanjak M, et al. Ibudilast - Phosphodiesterase Type 4 Inhibitor - Bi-Modal Therapy with Riluzole in Early [Not Requiring Non-Invasive Ventilation (NIV)] Cohort (EC) and Advanced [Requiring NIV] (ANC) Amyotrophic Lateral Sclerosis (ALS) Patients - Single-Center Adaptive Design Six-Month Double-Blind (DB) - Placebo-Controlled Phase 1b/2a Epoch Followed by Six-Month Open Label Extension (OLE) Epoch, Washout (WO) and Post-Washout Epoch (PWO) – Final Report and Future Directions (P6.465). *Neurology*. 2018;90(15 Supplement). Accessed December 2, 2021. https://n.neurology.org/content/90/15_Supplement/P6.465

45. Brooks B, Bravver E, Sanjak M, et al. Adaptive Design Single Center Phosphodiesterase Type 4 (PDE4) Inhibitor - Ibudilast (MN-166-ALS-1201) Phase 1b/2a Clinical Trial Double-Blind (DB) with Open Label Extensions (OLE) [NCT02238626] for Amyotrophic Lateral Sclerosis (ALS) Patients [1] Not Requiring Non-Invasive Ventilation (no-NIV) up to 5 Years (Early Cohort-EC) and [2] Requiring Non-Invasive Ventilation (NIV) up to 10 Years(Advanced NIV Cohort-ANC) from Disease Onset - Behavior of Creatinine as a Biomarker in Short Clinical Trials (P5.003). *Neurology*. 2016;86(16 Supplement). Accessed December 2, 2021. https://n.neurology.org/content/86/16_Supplement/P5.003

46. Fox RJ, Coffey CS, Conwit R, et al. Phase 2 Trial of Ibudilast in Progressive Multiple Sclerosis. *New England Journal of Medicine*. 2018;379(9):846-855. doi:10.1056/NEJMoa1803583

57. Fehlings MG, Ibrahim A, Tetreault L, et al. A global perspective on the outcomes of surgical decompression in patients with cervical spondylotic myelopathy: results from the

For peer review only

prospective multicenter AOSpine international study on 479 patients. *Spine*. 2015;40(17):1322-1328. doi:10.1097/BRS.0000000000000988

58. Fehlings MG, Wilson JR, Karadimas SK, Arnold PM, Kopjar B. Clinical evaluation of a neuroprotective drug in patients with cervical spondylotic myelopathy undergoing surgical treatment: design and rationale for the CSM-Protect trial. *Spine*. 2013;38(22 Suppl 1):S68-75. doi:10.1097/BRS.0b013e3182a7e9b0

59. Kopjar B, Tetreault L, Kalsi-Ryan S, Fehlings M. Psychometric properties of the modified Japanese Orthopaedic Association scale in patients with cervical spondylotic myelopathy. *Spine (Phila Pa 1976)*. 2015;40(1):E23-28. doi:10.1097/BRS.0000000000000648

60. Fehlings MG, Badhiwala JH, Ahn H, et al. Safety and efficacy of riluzole in patients undergoing decompressive surgery for degenerative cervical myelopathy (CSM-Protect): a multicentre, double-blind, placebo-controlled, randomised, phase 3 trial. *Lancet Neurol*. 2021;20(2):98-106. doi:10.1016/S1474-4422(20)30407-5

61. Moon ES, Karadimas SK, Yu WR, Austin JW, Fehlings MG. Riluzole attenuates neuropathic pain and enhances functional recovery in a rodent model of cervical spondylotic myelopathy. *Neurobiol Dis*. 2014;62:394-406. doi:10.1016/j.nbd.2013.10.020

62. Segerdahl M. Pain outcome variables--a never ending story? *Pain*. 2011;152(5):961-962. doi:10.1016/j.pain.2011.03.008

63. Davies BM, McHugh M, Elgheriani A, et al. Reported Outcome Measures in Degenerative Cervical Myelopathy: A Systematic Review. *PLoS One*. 2016;11(8). doi:10.1371/journal.pone.0157263

64. MacDowall A, Skeppholm M, Robinson Y, Olerud C. Validation of the visual analog scale in the cervical spine. *J Neurosurg Spine*. 2018;28(3):227-235. doi:10.3171/2017.5.SPINE1732

65. Parker SL, Godil SS, Shau DN, Mendenhall SK, McGirt MJ. Assessment of the minimum clinically important difference in pain, disability, and quality of life after anterior cervical discectomy and fusion: clinical article. *J Neurosurg Spine*. 2013;18(2):154-160. doi:10.3171/2012.10.SPINE12312

51. Davies BM, Khan DZ, Mowforth OD, et al. RE-CODE DCM (REsearch Objectives and Common Data Elements for Degenerative Cervical Myelopathy): A Consensus Process to Improve Research Efficiency in DCM, Through Establishment of a Standardized Dataset for Clinical Research and the Definition of the Research Priorities. *Global Spine J*. 2019;9(1 Suppl):65S-76S. doi:10.1177/2192568219832855

65. Brouwer WBF, van Exel NJA, van Gorp B, Redekop WK. The CarerQol instrument: a new instrument to measure care-related quality of life of informal caregivers for use in economic evaluations. *Qual Life Res*. 2006;15(6):1005-1021. doi:10.1007/s11136-005-5994-6

66. Tetreault L, Nouri A, Kopjar B, Côté P, Fehlings MG. The Minimum Clinically Important Difference of the Modified Japanese Orthopaedic Association Scale in Patients

For peer review only

For peer review only - http://bmjopen.bmj.com/site/about/guidelines.xhtml

with Degenerative Cervical Myelopathy. *Spine (Phila Pa 1976)*. 2015;40(21):1653-1659. doi:10.1097/BRS.0000000000001127

67. Tetreault L, Wilson JR, Kotter MRN, et al. Predicting the minimum clinically important difference in patients undergoing surgery for the treatment of degenerative cervical myelopathy. *Neurosurg Focus*. 2016;40(6):E14. doi:10.3171/2016.3.FOCUS1665

68. Tetreault L, Lange SF, Chotai S, et al. A Systematic Review of Definitions for Neurological Complications and Disease Progression in Patients Treated Surgically for Degenerative Cervical Myelopathy. *Spine (Phila Pa 1976)*. 2019;44(18):1318-1331. doi:10.1097/BRS.0000000000003066

69. Bretz F, Maurer W, Brannath W, Posch M. A graphical approach to sequentially rejective multiple test procedures. *Statistics in Medicine*. 2009;28(4):586-604. doi:10.1002/sim.3495

70. Benjamini Y, Hochberg Y. Controlling the False Discovery Rate: A Practical and Powerful Approach to Multiple Testing. *Journal of the Royal Statistical Society: Series B (Methodological)*. 1995;57(1):289-300. doi:10.1111/j.2517-6161.1995.tb02031.x

47. Chalmers I, Bracken MB, Djulbegovic B, et al. How to increase value and reduce waste when research priorities are set. *Lancet*. 2014;383(9912):156-165. doi:10.1016/S0140-6736(13)62229-1

48. Ergina PL, Cook JA, Blazeby JM, et al. Challenges in evaluating surgical innovation. *Lancet*. 2009;374(9695):1097-1104. doi:10.1016/S0140-6736(09)61086-2

49. Davies BM, Munro C, Khan DZ, et al. Outcomes of Degenerative Cervical Myelopathy From The Perspective of Persons Living With the Condition: Findings of a Semistructured Interview Process With Partnered Internet Survey. *Global Spine J*. Published online November 18, 2020:2192568220953811. doi:10.1177/2192568220953811

50. Davies BM, Mowforth OD, Khan DZ, et al. The development of lived experience-centered word clouds to support research uncertainty gathering in degenerative cervical myelopathy: results from an engagement process and protocol for their evaluation, via a nested randomized controlled trial. *Trials*. 2021;22(1):415. doi:10.1186/s13063-021-05349-8

52. Mowforth OD, Davies BM, Goh S, O'Neill CP, Kotter MRN. Research Inefficiency in Degenerative Cervical Myelopathy: Findings of a Systematic Review on Research Activity Over the Past 20 Years. *Global Spine J*. 2020;10(4):476-485. doi:10.1177/2192568219847439

53. Grodzinski B, Bestwick H, Bhatti F, et al. Research activity amongst DCM research priorities. *Acta Neurochir (Wien)*. Published online February 24, 2021. doi:10.1007/s00701-021-04767-6

54. Neurology TL. A focus on patient outcomes in cervical myelopathy. *The Lancet Neurology*. 2019;18(7):615. doi:10.1016/S1474-4422(19)30168-1

For peer review only

For peer review only - http://bmjopen.bmj.com/site/about/guidelines.xhtml

55. Mowforth OD, Davies BM, Kotter MR. The Use of Smart Technology in an Online Community of Patients With Degenerative Cervical Myelopathy. *JMIR Form Res*. 2019;3(2):e11364. doi:10.2196/11364

56. Davies B, Kotter M. Lessons From Recruitment to an Internet-Based Survey for Degenerative Cervical Myelopathy: Comparison of Free and Fee-Based Methods. *JMIR Res Protoc*. 2018;7(2):e18. doi:10.2196/resprot.6567

71. Waqar M, Wilcock J, Garner J, Davies B, Kotter M. Quantitative analysis of medical students' and physicians' knowledge of degenerative cervical myelopathy. *BMJ Open*. 2020;10(1):e028455. doi:10.1136/bmjopen-2018-028455

72. Kalsi-Ryan S, Rienmueller AC, Riehm L, et al. Quantitative Assessment of Gait Characteristics in Degenerative Cervical Myelopathy: A Prospective Clinical Study. *J Clin Med*. 2020;9(3):E752. doi:10.3390/jcm9030752

73. Bhagavatula ID, Bhat DI, Sasidharan GM, et al. Subclinical respiratory dysfunction in chronic cervical cord compression: a pulmonary function test correlation. *Neurosurg Focus*. 2016;40(6):E3. doi:10.3171/2016.3.FOCUS1647

74. Pope DH, Davies BM, Mowforth OD, Bowden AR, Kotter MRN. Genetics of Degenerative Cervical Myelopathy: A Systematic Review and Meta-Analysis of Candidate Gene Studies. *J Clin Med*. 2020;9(1). doi:10.3390/jcm9010282

75. Badran A, Davies BM, Bailey HM, Kalsi-Ryan S, Kotter MR. Is there a role for postoperative physiotherapy in degenerative cervical myelopathy? A systematic review. *Clin Rehabil*. Published online April 1, 2018:269215518766229. doi:10.1177/0269215518766229

76. Davies BM, McHugh M, Elgheriani A, et al. The reporting of study and population characteristics in degenerative cervical myelopathy: A systematic review. *PLOS ONE*. 2017;12(3):e0172564. doi:10.1371/journal.pone.0172564

77. Bogduk N, Govind J. Cervicogenic headache: an assessment of the evidence on clinical diagnosis, invasive tests, and treatment. *Lancet Neurol*. 2009;8(10):959-968. doi:10.1016/S1474-4422(09)70209-1

78. Sheather-Reid RB, Cohen ML. Psychophysical evidence for a neuropathic component of chronic neck pain. *Pain*. 1998;75(2-3):341-347. doi:10.1016/s0304-3959(98)00013-x

79. Voerman VF, van Egmond J, Crul BJ. Elevated detection thresholds for mechanical stimuli in chronic pain patients: support for a central mechanism. *Arch Phys Med Rehabil*. 2000;81(4):430-435. doi:10.1053/mr.2000.3777

80. Zaproudina N, Ming† Z, Närhi M. Sensory and sympathetic disorders in chronic non-specific neck pain. *Funct Neurol*. 2015;30(3):165-171. doi:10.11138/FNeur/2015.30.3.165

81. Westermann A, Rönnau AK, Krumova E, et al. Pain-associated mild sensory deficits without hyperalgesia in chronic non-neuropathic pain. *Clin J Pain*. 2011;27(9):782-789. doi:10.1097/AJP.0b013e31821d8fce

For peer review only - http://bmjopen.bmj.com/site/about/guidelines.xhtml

82. Blyufer A, Lhamo S, Tam C, Tariq I, Thavornwatanayong T, Mahajan SS. Riluzole: A neuroprotective drug with potential as a novel anti-cancer agent (Review). *Int J Oncol*. 2021;59(5):95. doi:10.3892/ijo.2021.5275

83. Lee S, Zhao X, Hatch M, Chun S, Chang E. Central Neuropathic Pain in Spinal Cord Injury. *Crit Rev Phys Rehabil Med*. 2013;25(3-4):159-172. doi:10.1615/CritRevPhysRehabilMed.2013007944

84. Kalsi-Ryan S, Singh A, Massicotte EM, et al. Ancillary outcome measures for assessment of individuals with cervical spondylotic myelopathy. *Spine (Phila Pa 1976)*. 2013;38(22 Suppl 1):S111-122. doi:10.1097/BRS.0b013e3182a7f499

85. Tetreault LA, Côté P, Kopjar B, Arnold P, Fehlings MG, AOSpine North America and International Clinical Trial Research Network. A clinical prediction model to assess surgical outcome in patients with cervical spondylotic myelopathy: internal and external validations using the prospective multicenter AOSpine North American and international datasets of 743 patients. *Spine J*. 2015;15(3):388-397. doi:10.1016/j.spinee.2014.12.145

86. Ahuja CS, Wilson JR, Nori S, et al. Traumatic spinal cord injury. *Nat Rev Dis Primers*. 2017;3(1):1-21. doi:10.1038/nrdp.2017.18

87. Fehlings MG, Wilson JR, Kopjar B, et al. Efficacy and safety of surgical decompression in patients with cervical spondylotic myelopathy: results of the AOSpine North America prospective multi-center study. *J Bone Joint Surg Am*. 2013;95(18):1651-1658. doi:10.2106/JBJS.L.00589

88. Martin AR, Leener BD, Cohen-Adad J, et al. Can microstructural MRI detect subclinical tissue injury in subjects with asymptomatic cervical spinal cord compression? A prospective cohort study. *BMJ Open*. 2018;8(4):e019809. doi:10.1136/bmjopen-2017-019809

89. Toyoda H, Nakamura H, Konishi S, Terai H, Takaoka K. Does chronic cervical myelopathy affect respiratory function? *J Neurosurg Spine*. 2004;1(2):175-178. doi:10.3171/spi.2004.1.2.0175

90. Kalsi-Ryan S, Beaton D, Ahn H, et al. Responsiveness, Sensitivity, and Minimally Detectable Difference of the Graded and Redefined Assessment of Strength, Sensibility, and Prehension, Version 1.0. *J Neurotrauma*. 2016;33(3):307-314. doi:10.1089/neu.2015.4217

91. McDermott A, Bolger C, Keating L, McEvoy L, Meldrum D. Reliability of three-dimensional gait analysis in cervical spondylotic myelopathy. *Gait Posture*. 2010;32(4):552-558. doi:10.1016/j.gaitpost.2010.07.019

92. Nagai T, Takahashi Y, Endo K, Ikegami R, Ueno R, Yamamoto K. Analysis of spastic gait in cervical myelopathy: Linking compression ratio to spatiotemporal and pedobarographic parameters. *Gait Posture*. 2018;59:152-156. doi:10.1016/j.gaitpost.2017.10.013

For peer review only - http://bmjopen.bmj.com/site/about/guidelines.xhtml

Figure Legend.

Figure 1: Trial Flow Chart.
Eligible and consenting participants will be randomised to an intervention or control arm and followed up for 12 months after surgery.

For peer review only

For peer review only - http://bmjopen.bmj.com/site/about/guidelines.xhtml

Figure 1. Trial Flow Chart.

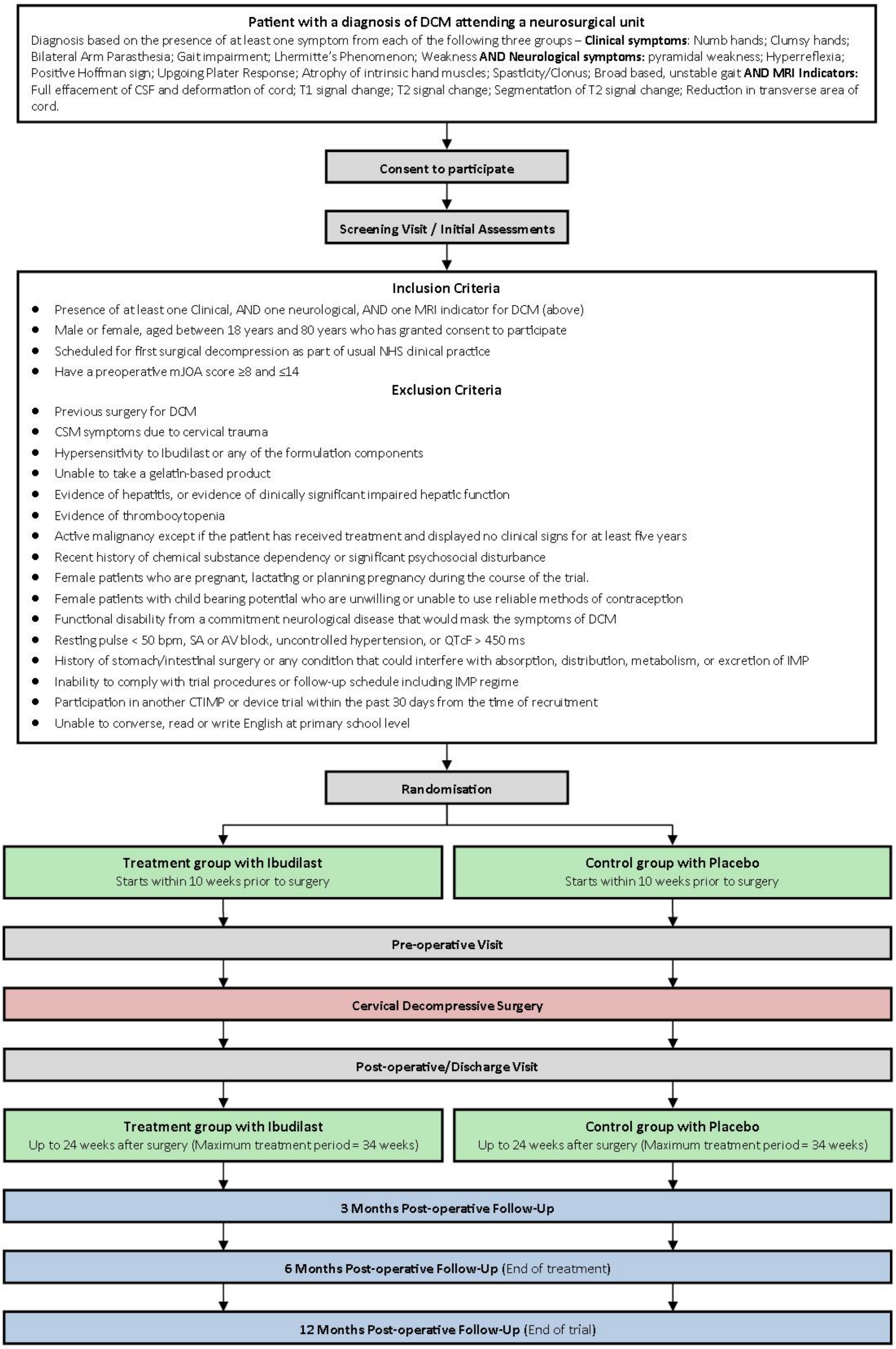

SPIRIT
STANDARD PROTOCOL ITEMS: RECOMMENDATIONS FOR INTERVENTIONAL TRIALS

SPIRIT 2013 Checklist: Recommended items to address in a clinical trial protocol and related documents*

Section/item	Item No	Description
**Administrative information**		
Title	1	Descriptive title identifying the study design, population, interventions, and, if applicable, trial acronym – Present, Page 1
Trial registration	2a	Trial identifier and registry name. If not yet registered, name of intended registry – Present, Page 3 in Ethics and Dissemination Section.
	2b	All items from the World Health Organization Trial Registration Data Set – Present throughout the Manuscript.
Protocol version	3	Date and version identifier – Present, Page 22, Footnotes
Funding	4	Sources and types of financial, material, and other support - Present
Roles and responsibilities	5a	Names, affiliations, and roles of protocol contributors -Present, Page 1
	5b	Name and contact information for the trial sponsor – Present, Present Page 1,2
	5c	Role of study sponsor and funders, if any, in study design; collection, management, analysis, and interpretation of data; writing of the report; and the decision to submit the report for publication, including whether they will have ultimate authority over any of these activities – N/a
	5d	Composition, roles, and responsibilities of the coordinating centre, steering committee, endpoint adjudication committee, data management team, and other individuals or groups overseeing the trial, if applicable (see Item 21a for data monitoring committee) – Present, Page 15 and 16
**Introduction**		
Background and rationale	6a	Description of research question and justification for undertaking the trial, including summary of relevant studies (published and unpublished) examining benefits and harms for each intervention – Present, pages 4-7
	6b	Explanation for choice of comparators – Present, page 12-14
Objectives	7	Specific objectives or hypotheses -Present, Page 12

For peer review only - http://bmjopen.bmj.com/site/about/guidelines.xhtml

Trial design	8	Description of trial design including type of trial (eg, parallel group, crossover, factorial, single group), allocation ratio, and framework (eg, superiority, equivalence, noninferiority, exploratory) – Present. Page 8

Methods: Participants, interventions, and outcomes

Study setting	9	Description of study settings (eg, community clinic, academic hospital) and list of countries where data will be collected. Reference to where list of study sites can be obtained – Present, page 8
Eligibility criteria	10	Inclusion and exclusion criteria for participants. If applicable, eligibility criteria for study centres and individuals who will perform the interventions (eg, surgeons, psychotherapists) – Present, page 9/10
Interventions	11a	Interventions for each group with sufficient detail to allow replication, including how and when they will be administered – Present, page 11
	11b	Criteria for discontinuing or modifying allocated interventions for a given trial participant (eg, drug dose change in response to harms, participant request, or improving/worsening disease) – Present, page 11
	11c	Strategies to improve adherence to intervention protocols, and any procedures for monitoring adherence (eg, drug tablet return, laboratory tests) – n/a
	11d	Relevant concomitant care and interventions that are permitted or prohibited during the trial – n/a
Outcomes	12	Primary, secondary, and other outcomes, including the specific measurement variable (eg, systolic blood pressure), analysis metric (eg, change from baseline, final value, time to event), method of aggregation (eg, median, proportion), and time point for each outcome. Explanation of the clinical relevance of chosen efficacy and harm outcomes is strongly recommended – Present , Page 12-14
Participant timeline	13	Time schedule of enrolment, interventions (including any run-ins and washouts), assessments, and visits for participants. A schematic diagram is highly recommended (see Figure) – Present, page 11
Sample size	14	Estimated number of participants needed to achieve study objectives and how it was determined, including clinical and statistical assumptions supporting any sample size calculations – Present, page 14
Recruitment	15	Strategies for achieving adequate participant enrolment to reach target sample size – N/a

Methods: Assignment of interventions (for controlled trials)

Allocation:

For peer review only - http://bmjopen.bmj.com/site/about/guidelines.xhtml

Sequence generation	16a	Method of generating the allocation sequence (eg, computer-generated random numbers), and list of any factors for stratification. To reduce predictability of a random sequence, details of any planned restriction (eg, blocking) should be provided in a separate document that is unavailable to those who enrol participants or assign interventions – Present, page 11, Enrolment and Randomisation Section
Allocation concealment mechanism	16b	Mechanism of implementing the allocation sequence (eg, central telephone; sequentially numbered, opaque, sealed envelopes), describing any steps to conceal the sequence until interventions are assigned – Present, page 11, Enrolment and Randomisation Section
Implementation	16c	Who will generate the allocation sequence, who will enrol participants, and who will assign participants to interventions – Present
Blinding (masking)	17a	Who will be blinded after assignment to interventions (eg, trial participants, care providers, outcome assessors, data analysts), and how – Present, Page 11
	17b	If blinded, circumstances under which unblinding is permissible, and procedure for revealing a participant's allocated intervention during the trial – Present, Page 11
**Methods: Data collection, management, and analysis**		
Data collection methods	18a	Plans for assessment and collection of outcome, baseline, and other trial data, including any related processes to promote data quality (eg, duplicate measurements, training of assessors) and a description of study instruments (eg, questionnaires, laboratory tests) along with their reliability and validity, if known. Reference to where data collection forms can be found, if not in the protocol – Present, 14-17
	18b	Plans to promote participant retention and complete follow-up, including list of any outcome data to be collected for participants who discontinue or deviate from intervention protocols – N/a
Data management	19	Plans for data entry, coding, security, and storage, including any related processes to promote data quality (eg, double data entry; range checks for data values). Reference to where details of data management procedures can be found, if not in the protocol – page 15
Statistical methods	20a	Statistical methods for analysing primary and secondary outcomes. Reference to where other details of the statistical analysis plan can be found, if not in the protocol – 16-17
	20b	Methods for any additional analyses (eg, subgroup and adjusted analyses) – Present, 16-17

For peer review only - http://bmjopen.bmj.com/site/about/guidelines.xhtml

	20c	Definition of analysis population relating to protocol non-adherence (eg, as randomised analysis), and any statistical methods to handle missing data (eg, multiple imputation) – Present , page 16-17
**Methods: Monitoring**		
Data monitoring	21a	Composition of data monitoring committee (DMC); summary of its role and reporting structure; statement of whether it is independent from the sponsor and competing interests; and reference to where further details about its charter can be found, if not in the protocol. Alternatively, an explanation of why a DMC is not needed – Present, page 15-16
	21b	Description of any interim analyses and stopping guidelines, including who will have access to these interim results and make the final decision to terminate the trial – Present, Page 16-17
Harms	22	Plans for collecting, assessing, reporting, and managing solicited and spontaneously reported adverse events and other unintended effects of trial interventions or trial conduct – Present, page 16-17
Auditing	23	Frequency and procedures for auditing trial conduct, if any, and whether the process will be independent from investigators and the sponsor – Present, page 16-17
**Ethics and dissemination**		
Research ethics approval	24	Plans for seeking research ethics committee/institutional review board (REC/IRB) approval – REC Approval gained, page 21
Protocol amendments	25	Plans for communicating important protocol modifications (eg, changes to eligibility criteria, outcomes, analyses) to relevant parties (eg, investigators, REC/IRBs, trial participants, trial registries, journals, regulators) – Present, page 21
Consent or assent	26a	Who will obtain informed consent or assent from potential trial participants or authorised surrogates, and how (see Item 32) – present, page 21
	26b	Additional consent provisions for collection and use of participant data and biological specimens in ancillary studies, if applicable – Present , page 21
Confidentiality	27	How personal information about potential and enrolled participants will be collected, shared, and maintained in order to protect confidentiality before, during, and after the trial – Present, page 21
Declaration of interests	28	Financial and other competing interests for principal investigators for the overall trial and each study site – Present. Page 22

review only

For peer review only - http://bmjopen.bmj.com/site/about/guidelines.xhtml

Access to data	29	Statement of who will have access to the final trial dataset, and disclosure of contractual agreements that limit such access for investigators – Present, page 15
Ancillary and post-trial care	30	Provisions, if any, for ancillary and post-trial care, and for compensation to those who suffer harm from trial participation – n/a
Dissemination policy	31a	Plans for investigators and sponsor to communicate trial results to participants, healthcare professionals, the public, and other relevant groups (eg, via publication, reporting in results databases, or other data sharing arrangements), including any publication restrictions – Present, page 21
	31b	Authorship eligibility guidelines and any intended use of professional writers – Included in the submission
	31c	Plans, if any, for granting public access to the full protocol, participant-level dataset, and statistical code(n/a)
**Appendices**		
Informed consent materials	32	Model consent form and other related documentation given to participants and authorised surrogates – (attached)
Biological specimens	33	Plans for collection, laboratory evaluation, and storage of biological specimens for genetic or molecular analysis in the current trial and for future use in ancillary studies, if applicable – N/a

*It is strongly recommended that this checklist be read in conjunction with the SPIRIT 2013 Explanation & Elaboration for important clarification on the items. Amendments to the protocol should be tracked and dated. The SPIRIT checklist is copyrighted by the SPIRIT Group under the Creative Commons "Attribution-NonCommercial-NoDerivs 3.0 Unported" license.

For peer review only
